# Supplementary material for: Evaluating quality in adolescent mental health services: a systematic review
Source: BMJ Open. 2021 May 9;11(5):e044929. doi: 10.1136/bmjopen-2020-044929 (PMC8112446; doi:10.1136/bmjopen-2020-044929)
Supplement: Supplementary data [file bmjopen-2020-044929supp002.pdf]

Supplemental Material 2. Process and Output Criteria used for WHO Quality Standards to Select Studies[1]

For *adolescent mental health literacy*, this involved identifying and assessing studies using the following criteria:

	**Adolescent mental health literacy: Selection criteria for studies**
**PROCESS**	the provision of mental health education and counselling to adolescents by healthcare providers and services that are age and developmentally appropriate
	the provision of information about the availability of mental health, social, and other services to adolescents by healthcare providers and services
	outreach activities by the healthcare facility to promote mental health and increase the use of adolescent mental health services
**OUTPUT**	adolescents increased knowledge about mental health and awareness about mental health services

For *appropriate package of services*, we identified and assessed studies according to the following:

	**Appropriate package of services: Selection criteria for studies**
**PROCESS**	within healthcare services, the provision of a package of mental health information, counselling, diagnostic, treatment and care services to adolescents that meets their needs
	referral to appropriate services
**OUTPUT**	adolescents receive a package of services that meets their needs, within healthcare or referral services

For *provider competencies*, this involved identifying and assessing studies using the following criteria:

	**Provider competencies: Selection criteria for studies**
**PROCESS**	healthcare providers adhere to and implement adolescent mental health evidence-based guidelines and protocols
	healthcare providers provide friendly services to adolescents and respect their rights to information, privacy, confidentiality, non-discrimination, non-judgement attitude, and respectful care
**OUTPUT**	adolescents receive effective mental health services
	adolescents obtain mental health services that are friendly, supportive, respectful, non-discriminatory and non-judgemental manner, and know their rights in healthcare
	adolescents receive from the healthcare facility accurate, age-appropriate and clear information to facilitate informed choice

References

1. WHO. Global standards for quality health care services for adolescents. Geneva: World Health Organization, 2015.
