# Supplementary material for: Evaluating quality in adolescent mental health services: a systematic review
Source: BMJ Open. 2021 May 9;11(5):e044929. doi: 10.1136/bmjopen-2020-044929 (PMC8112446; doi:10.1136/bmjopen-2020-044929)
Supplement: Supplementary data [file bmjopen-2020-044929supp004.pdf]

Supplemental Material 4. Table of Included Studies with Additional Information

Author	Country	Service, Intervention, Study description
Davidson et al (2019)	USA	Tablet trauma-focussed cognitive behavioural therapy (TF-CBT) included 11 activities that focussed on breathing retraining, muscle relaxation, affective regulation and cognitive coping; other activities are specific to the TF-CBT model, including psychoeducation relating to traumatic events, safety education, and gradual exposure.
Jager et al (2017)	The Netherlands	Patient-centred communication through Take Care study
Ougrin et a (2018)	UK	Supported Discharge Service Versus Inpatient Care Evaluation (SITE) project comprised of intensive community-based treatment, which involved customised care plans of intensive case management, community/home treatment, day care in hospital
Dion et al (2010)	Canada	Crisis Intervention Program that investigated the capability of emergency department staff to provide emergency assessments to adolescents with mental health issues presenting to the emergency department
Spenser et al (2009)	Canada	CHAT project which aimed to increase collaboration between community paediatricians and mental health clinicians: hired and integrated paediatrician within mental health team; surveyed clinicians about perceived needs; and provided continuing education based on identified needs
Ayton et al (2013)	UK	Audit Care Programme Approach (CPA) care plans within Child and adolescent mental health services (CAMHS) and identify service user involvement in all aspects of mental healthcare<br><br>This includes involving them in a needs assessment, developing and reviewing the care plan; and sharing responsibilities between the patient, carers, and relevant professional agencies.
Aupont et al (2013)	USA	Targeted Child Psychiatric Services that implemented a care model that facilitates adolescent's access to specialised mental health services, investigating whether the adolescent remains in specialised mental health services or returns to paediatric primary care
Simmons et al (2016)	Australia	*headspace* implemented an online decision aid for adolescent and youth depression was developed. The aim of the intervention was help young people make evidence-informed decisions about their treatment, make them feel involved, adhere to treatment and reduce symptoms, and increase satisfaction
Irvine (2020)	Ireland	Cross-sectional survey of child and adolescents' experiences with mental health care, using quality indicator clusters focussing on (i) information and access; (ii) facilities and services; and (iii) quality of care.

Jager et al (2014)	The Netherlands	In the Take Care study, Client-centred communication (measured using the Consumer Quality Index) affects adolescents participation and learning in their mental healthcare
Stevens et al (2009)	USA	Less intensive Telephone support services (TSS) intervention focussed on services and not symptoms
Anderson et al (2012)	Australia	Body signs, Relaxation, Active helpful thoughts, Victory you're your fears, Enjoy! Reward yourself (BRAVE) for Teenagers-Online project:<br><br>Develop working alliance during online cognitive behaviour therapy (CBT) treatment for youth anxiety.
Kapp et al (2017)	Switzerland	Identifying what influenced perceived quality of care (therapeutic alliance) in CAMHS
Cairns et al (2019)	Australia	In *headspace* services, influence of goal setting frequency and quality on patient retention
Ringle et al (2019)	USA	The Resiliency and Disease Management initiative, or an evidence-based practice that includes Child and Adolescent Texas Recommended Assessment Guidelines, a comprehensive list of service packages that indicates level of service intensity and evidence-based practice (e.g., CBT)
Sattler et al (2019)	USA	Non-profit provider of medical and mental health services<br><br>evidence-based assessments
Sattler et al (2016)	USA	Non-profit provider of medical and mental health services<br><br>use of evidence-based assessment techniques, including structured interviews, rating scales, DSM-IV-TR and agreement at primary care facilities, general mental health clinics, and specialty clinics for anxiety disorders
Higa-McMilan et al 2017)	USA	Use of Practices derived from the evidence base (PDEB)
Rukundo et al (2020)	Uganda	Two-year diploma programme that focusses on basic training of health care providers (psychiatrists, medical officers, psychologists, psychiatric clinical officers, occupational therapists, psychiatric nurses, general nurses, public health officers, social workers, human resource officers) towards assessing, recognising and managing child and adolescent mental health.
Bardach et al (2020)	USA	Using the Children's Core Set of quality measures for children and adolescents that have used mental health services

Acronyms: Trauma focussed cognitive behavioural therapy (TF-CBT); randomized controlled trial (RCT); Supported Discharge Service Versus Inpatient Care Evaluation (SITE); emergency department (ED); Child and adolescent mental health services (CAMHS); Care programme approach (CPA); Telephone support service (TSS); Body signs, Relaxation, Active helpful thoughts, Victory over your fears, Enjoy! Reward yourself (BRAVE); The Choice and Partnership Approach (CAPA); Practices derived from the evidence base (PDEB); Managing and Adapting Practice (MAP); Practices derived from the evidence base (PDEB)
