# Supplementary material for: Evaluating quality in adolescent mental health services: a systematic review
Source: BMJ Open. 2021 May 9;11(5):e044929. doi: 10.1136/bmjopen-2020-044929 (PMC8112446; doi:10.1136/bmjopen-2020-044929)
Supplement: Supplementary data [file bmjopen-2020-044929supp001.pdf]

NIHR | National Institute for Health Research

PROSPERO
International prospective register of systematic reviews

Evaluating quality in adolescent mental health service delivery: a systematic review

Meaghen Quinlan-Davidson, Kathryn Roberts, Delan Devakumar, Susan Sawyer, Ligia Kiss

Supplemental Material 1. PROSPERO Registration

Citation

Meaghen Quinlan-Davidson, Kathryn Roberts, Delan Devakumar, Susan Sawyer, Ligia Kiss. Evaluating quality in adolescent mental health service delivery: a systematic review. PROSPERO 2020 CRD42020161318 Available from:
https://www.crd.york.ac.uk/prospero/display_record.php?ID=CRD42020161318

Review question

What is the global evidence on the aspects of quality of adolescent mental health services (based on the WHO Global standards for quality healthcare services for adolescents) including adolescent mental health literacy, provider competencies, and appropriate packages of services for adolescents with depressive, anxiety, and post-traumatic stress disorders?

Searches

-2008-2018

-Sources include (but are not limited to) PubMed, PscyINFO, MEDLINE, EMBASE, LILACS

-Reference checking of articles generated from electronic database

-Hand searching of references of identified manuscripts

-No restriction on language

Types of study to be included

Evaluations or assessments of RCTs, case-control, cohort, cross-sectional, longitudinal, mixed-methods (quantitative data only).

Condition or domain being studied

Quality in adolescent mental health services.

Participants/population

Study participants include individual patients (10-19 years of age) using mental healthcare services, or adolescents in the community asked where they would seek services for depression, anxiety, or PTSD for themselves; and providers of mental health services, including general practitioners, psychologists, and psychiatrists.

Where study participants include children and adolescents together or adolescents and youth together, the study will be included when the results are sufficiently disaggregated that they can be interpreted for at least part of the 10-19 year old age span.

Intervention(s), exposure(s)

Studies that evaluate or assess aspects of quality measures (as conforms to the WHO Global standards for quality healthcare services for adolescents) applied to existing adolescent mental health services, who have used, are currently using, or could potentially seek to use mental health services, or are exposed to interventions or strategies within established mental health services, regardless of health facility level.

Comparator(s)/control

For intervention studies only: Intervention vs. control arm (randomized trial), quasi-experimental (open trial with pre- and post-intervention design or matched control group).

Main outcome(s)

Studies that include quality, as conforms to the WHO WHO Global standards for quality healthcare services for adolescents, including:

-Adolescents' health literacy (The health facility implements systems to ensure that adolescents are knowledgeable about their own health, and they know where and when to obtain health services);

-Appropriate package of services (The health facility provides a package of information, counselling, diagnostic, treatment and care services that fulfils the needs of all adolescents. Services are provided in the facility and through referral linkages and outreach);

-Providers' competencies (Health care providers demonstrate the technical competence required to provide effective health services to adolescents. Both health-care providers and support staff respect, protect and fulfil adolescents' rights to information, privacy, confidentiality, non-discrimination, non-judgmental attitude and respect)

* Measures of effect

Not applicable.

Additional outcome(s)

None.

* Measures of effect

Not applicable.

Data extraction (selection and coding)

All results from the above mentioned search strategy will be exported to Endnote, an electronic reference manager; duplicates will be removed. Independently, two authors (MQD and KR) will screen the titles and abstracts of all studies identified through the search strategy that potentially meet the inclusion criteria mentioned above. The full text of these potentially eligible studies will be retrieved and independently assessed for eligibility by the two team members. The main data to be extracted will include study design, publication type, area of quality, reason (if any) for exclusion), type of service and mental health issue, purpose of study, conceptualisation of quality or quality in relation to or effect of quality on, intervention/measured used, sample, outcomes, and location. Disagreement over the eligibility of particular studies will be resolved through discussion. Discussion will be held to resolve any discrepancies, with additional authors when needed.

Risk of bias (quality) assessment

NIH Quality Assessment Tool

Strategy for data synthesis

Random effects meta-analyses will be conducted to determine intervention effects, if sufficient data exist and levels of heterogeneity are appropriate. A minimum of three studies will be required per meta-analysis. If data cannot be pooled between intervention studies, findings will be reported in narrative synthesis. Results will be organised and interpreted according to the WHO Global Standards for Quality Healthcare Services for Adolescents. This will include the number of studies that address each element of quality, as well as a summary of the results of each study. Also, results on the conceptualisation of quality or how quality was mentioned in the article will be summarised, geographic location of the studies, and a search and selection strategy will be presented. A table, summarising the characteristics of the study, will be included that provides information on the service, intervention description, study/evaluation design, target population, element of quality addressed, results, and quality assessment will be included.

Analysis of subgroups or subsets

Not applicable.

Contact details for further information

Meaghen Quinlan-Davidson
meaghen.quinlan-davidson.17@ucl.ac.uk

Organisational affiliation of the review
Institute for Global Health, University College London

Review team members and their organisational affiliations
Miss Meaghen Quinlan-Davidson. Institute for Global Health, University College London
Miss Kathryn Roberts. Institute for Global Health, University College London
Dr Delan Devakumar. Institute for Global Health, University College London
Dr Susan Sawyer. Centre for Adolescent Health, Royal Children's Hospital, Melbourne, Australia
Dr Ligia Kiss. Institute for Global Health, University College London

Type and method of review
Service delivery, Systematic review

Anticipated or actual start date
12 November 2019

Anticipated completion date
31 January 2020

Funding sources/sponsors
None.

Conflicts of interest

Language
English

Country
England

Stage of review
Review Ongoing

Subject index terms status
Subject indexing assigned by CRD

Subject index terms
Adolescent; Adolescent Health Services; Delivery of Health Care; Humans; Mental Health Services

Date of registration in PROSPERO
27 January 2020

Date of first submission
05 December 2019

Details of any existing review of the same topic by the same authors

Stage of review at time of this submission

Stage	Started	Completed
Preliminary searches	Yes	No
Piloting of the study selection process	Yes	No
Formal screening of search results against eligibility criteria	Yes	No
Data extraction	No	No
Risk of bias (quality) assessment	No	No
Data analysis	No	No

The record owner confirms that the information they have supplied for this submission is accurate and complete and they understand that deliberate provision of inaccurate information or omission of data may be construed as scientific misconduct.

The record owner confirms that they will update the status of the review when it is completed and will add publication details in due course.

Versions

27 January 2020

PROSPERO
This information has been provided by the named contact for this review. CRD has accepted this information in good faith and registered the review in PROSPERO. The registrant confirms that the information supplied for this submission is accurate and complete. CRD bears no responsibility or liability for the content of this registration record, any associated files or external websites.
