# Supplementary material for: Evaluating quality in adolescent mental health services: a systematic review
Source: BMJ Open. 2021 May 9;11(5):e044929. doi: 10.1136/bmjopen-2020-044929 (PMC8112446; doi:10.1136/bmjopen-2020-044929)
Supplement: Author's manuscript [file bmjopen-2020-044929.draft_revisions.pdf]

BMJ Open

BMJ Open is committed to open peer review. As part of this commitment we make the peer review history of every article we publish publicly available.

When an article is published we post the peer reviewers' comments and the authors' responses online. We also post the versions of the paper that were used during peer review. These are the versions that the peer review comments apply to.

The versions of the paper that follow are the versions that were submitted during the peer review process. They are not the versions of record or the final published versions. They should not be cited or distributed as the published version of this manuscript.

BMJ Open is an open access journal and the full, final, typeset and author-corrected version of record of the manuscript is available on our site with no access controls, subscription charges or pay-per-view fees (http://bmjopen.bmj.com).

If you have any questions on BMJ Open's open peer review process please email info.bmjopen@bmj.com

BMJ Open

Evaluating Quality in Adolescent Mental Health Services: A Systematic Review

Journal:	*BMJ Open*
Manuscript ID	bmjopen-2020-044929
Article Type:	Original research
Date Submitted by the Author:	18-Sep-2020
Complete List of Authors:	Quinlan-Davidson, Meaghen; University College London Institute for Global Health,<br>Roberts, Kathryn; University College London Institute for Global Health<br>Devakumar, Delan; University College London Medical School,<br>Sawyer, Susan; The Royal Children's Hospital Melbourne, Centre for Adolescent Health; The University of Melbourne Faculty of Medicine Dentistry and Health Sciences, Department of Paediatrics<br>Cortez, Rafael; World Bank Group<br>Kiss, Lígia; University College London, Institute for Global Health
Keywords:	Quality in health care < HEALTH SERVICES ADMINISTRATION & MANAGEMENT, Child & adolescent psychiatry < PSYCHIATRY, MENTAL HEALTH, International health services < HEALTH SERVICES ADMINISTRATION & MANAGEMENT

SCHOLARONE™
Manuscripts

I, the Submitting Author has the right to grant and does grant on behalf of all authors of the Work (as defined in the below author licence), an exclusive licence and/or a non-exclusive licence for contributions from authors who are: i) UK Crown employees; ii) where BMJ has agreed a CC-BY licence shall apply, and/or iii) in accordance with the terms applicable for US Federal Government officers or employees acting as part of their official duties; on a worldwide, perpetual, irrevocable, royalty-free basis to BMJ Publishing Group Ltd ("BMJ") its licensees and where the relevant Journal is co-owned by BMJ to the co-owners of the Journal, to publish the Work in this journal and any other BMJ products and to exploit all rights, as set out in our licence.

The Submitting Author accepts and understands that any supply made under these terms is made by BMJ to the Submitting Author unless you are acting as an employee on behalf of your employer or a postgraduate student of an affiliated institution which is paying any applicable article publishing charge ("APC") for Open Access articles. Where the Submitting Author wishes to make the Work available on an Open Access basis (and intends to pay the relevant APC), the terms of reuse of such Open Access shall be governed by a Creative Commons licence – details of these licences and which Creative Commons licence will apply to this Work are set out in our licence referred to above.

Other than as permitted in any relevant BMJ Author's Self Archiving Policies, I confirm this Work has not been accepted for publication elsewhere, is not being considered for publication elsewhere and does not duplicate material already published. I confirm all authors consent to publication of this Work and authorise the granting of this licence.

For peer review only

Title: Evaluating Quality in Adolescent Mental Health Services: A Systematic Review

Meaghen Quinlan-Davidson[1], Kathryn J. Roberts[1], Delan Devakumar[1], Susan M. Sawyer[2], Rafael Cortez[3], Ligia Kiss[1]

[1] Institute for Global Health, University College London, 30 Guilford St, London WC1N 1DP, United Kingdom;
[2] Royal Children's Hospital, Flemington Road, Parkville, Victoria 2052 Australia;
[3] The World Bank, 1818 H Street, NW Washington DC 20433 USA

Correspondence to:
Meaghen Quinlan-Davidson
Institute for Global Health
University College London
London
WC1N 1DP
United Kingdom
Email: meaghen.quinlan-davidson.17@ucl.ac.uk
Phone: +44 (0)20 7679 2000

Word count: 4,527 words

For peer review only

ABSTRACT

Objectives. To evaluate the quality of adolescent mental health service provision globally, according to the WHO Global Standards of adolescent mental health literacy, appropriate package of services, and provider competencies.

Design and data sources. Systematic review of 5 databases, and screening of eligible articles, from 1 January 2008 to 31 December 2019.

Study eligibility criteria. We focussed on quantitative and mixed-method studies that evaluated adolescent mental health literacy, appropriate package of services, and provider competencies in mental health services targeted depression, anxiety, and post-traumatic stress disorder among adolescents (10-19 years). This included adolescents who have used, currently using, or are exposed to interventions or strategies within established mental health services.

Study appraisal and synthesis methods. Study quality was assessed using the National Institutes for Health Study Quality Assessment Tools. Data was extracted and grouped based on WHO quality standards.

Results. Of the 19,586 studies identified, 17 articles were included. All of the studies came from high-income countries. Most of the studies did not conceptualise quality. Results found that an online decision aid was evaluated to increase adolescent mental health literacy. Studies that targeted an appropriate package of services evaluated the quality of engagement between the therapist and adolescent, patient-centred communication, mental health service use, linkages to mental health services, health facility culture, and intensive community treatment. Provider competencies focussed on studies that evaluated confidence in managing and referring adolescents, collaboration between health facility levels, evidence-based practices, and technology use. Study quality was fair.

Conclusions and Implications. There is limited evidence on quality measures (as conforms to the WHO Global Standards) in adolescent mental health services, pointing to a critical evidence gap for adolescent mental health services. There are several challenges to overcome, including a need to develop a standard consensus on quality and methods to measure quality in these settings.

PROSPERO registration number. CRD42020161318

For peer review only - http://bmjopen.bmj.com/site/about/guidelines.xhtml

Strength and limitations of this study

- This is the first review to investigate quality measures for adolescent mental health services globally.
- The review highlights a critical gap in evidence on quality in adolescent mental health services.
- This review was limited to mental health services to adolescents in health facilities, although we acknowledge there are various sources outside of health services, within community and schools, that provide information and services to adolescents on mental health.
- The review investigated adolescents with suspected or diagnosed cases of depression, anxiety, and PTSD and not those with other mental health conditions.

For peer review only

For peer review only - http://bmjopen.bmj.com/site/about/guidelines.xhtml

INTRODUCTION

Globally, a significant burden of disease is due to mental health disorders.[1-3] Symptoms largely emerge during childhood, adolescence, and young adulthood,[1-4] with estimates suggesting that by 24 years of age, 75% of adult mental health disorders have appeared.[5, 6] Current global estimates indicate that 14.8% of young people aged 10-24 years have a mental health disorder,[7] with the most common being unipolar depressive disorders, anxiety disorders, and self-harm.[5] Depressive disorders rank as the fourth highest contributor to disability adjusted life years (DALYs) in 10-14 year olds (4.3%) and the third highest among 15-19 year olds (5.6%). Meanwhile, depressive and anxiety disorders rank among the top six contributors to years lived with disability (YLDs) for 10-14 year olds (7.4% and 5.0%, respectively) and among the top five for 15-19 year olds (11.2% and 5.8%, respectively).[3] In addition, a meta-analysis found that 16% of children and adolescents (2-18 years of age) develop post-traumatic stress disorder (PTSD) after exposure to a traumatic event.[8] Mental health conditions have short- and long-term health and social economic impacts including school dropout, social isolation, substance misuse, chronic unemployment, and high economic costs for families, communities and health systems.[1-4] Yet, despite the increased attention on adolescent mental health in recent years, adolescents with mental health conditions experience worse quality of care in comparison to other age groups.[9] Poor quality healthcare is associated with worse health outcomes, comorbid conditions, and a lack of trust in the health system. It also places the adolescent at an increased risk of death due to inadequate healthcare management, treatment and follow-up.[9]

Quality in mental healthcare is an important component of service delivery that contributes to adolescents seeking, receiving, and continuing care. The Institute of Medicine (IOM) and World Health Organization (WHO) define quality healthcare as “the degree to which health services for individuals and populations increase the likelihood of desired health outcomes and are consistent with current professional knowledge”.[10] Adolescents benefit from mental health services that are adolescent-friendly, defined as accessible, acceptable, equitable, appropriate, and effective.[11,12]

There are several multifaceted barriers that influence quality in mental healthcare services (table 1).

For peer review only - http://bmjopen.bmj.com/site/about/guidelines.xhtml

Table 1. Health Service Barriers to Quality Adolescent Mental Health Services

**Health Service Barriers**
• Limited financial resources
• Limited literacy on quality in mental healthcare, with limited or lack of quality criteria or measures
• Lack of routine assessment of quality in services
• Limited availability of specialised mental health care providers or those appropriately trained in mental health to engage and respond to adolescent's mental health needs
• Healthcare provider stigma, as a behavioural barrier to quality mental healthcare
• Lack of information, education, and engagement, from the health system to the community about adolescent mental health services and promotion of adolescent mental health literacy
• Limited adolescent patient involvement in making decisions about their treatment and care plans, ensuring patient-centred care
• Lack of youth-friendly, or patient-centred, services that ensure privacy, confidentiality (including confidentiality from parents and other service providers), respect, high-quality communication, and services that are judgement- and embarrassment free
• Lack of an equitable (geographically and socioeconomically) distribution of mental health services
• Overburdened primary care systems which make it hard to integrate adolescent mental health services
• Lack of mental health leadership from policy-and decision-makers at local and national government levels to champion quality in adolescent mental health services

Sources: Knaak S, Patten S, Ungar T. Mental illness stigma as a quality-of-care problem. *Lancet Psychiatry* 2015;2(10):863-4; Saraceno B, van Ommeren M, Batniji R, et al. Barriers to improvement of mental health services in low-income and middle-income countries. *Lancet* 2007;370(9593):1164-74; McPherson A. Adolescents in primary care. *Bmj* 2005;330(7489):465-7; Svirydzenka N, Ronzoni P, Dogra N. Meaning and barriers to quality care service provision in Child and Adolescent Mental Health Services: Qualitative study of stakeholder perspectives. *BMC Health Serv Res* 2017;17(1):151.

For adolescents and their families, barriers to quality mental healthcare start with access to these services. The stigma and discrimination associated with mental health disorders, as well as how adolescents, families, communities, and health services conceptualise emotional suffering and distress are significant barriers to access and use of quality mental health services [13]. Even in well-resourced contexts where mental health services are available, social and cultural conceptualisations of mental health influence the uptake and subsequent coverage of adolescent mental health services [13]. Adolescents are often less-experienced users of mental health services, with inadequate mental health literacy, including literacy about quality of care. They value privacy, confidentiality, and patient-centred care, which includes respect, high-quality communication, and a therapist who is responsive to their needs. They seek to avoid judgement or embarrassment, and fear that their parents will be informed, influencing their uptake of health services [4].

There have been several global initiatives focussing on quality of healthcare in recent years (box 1). In 2015, the WHO developed *Global standards for quality health care services for adolescents* to support health service delivery and quality improvements for adolescents in

For peer review only - http://bmjopen.bmj.com/site/about/guidelines.xhtml

primary and referral level facilities.[14] The aim of the standards is to increase adolescents' use of health services, improve health outcomes, ensure a minimal level of quality, and fulfil their rights' to health care.[14] There are eight standards, all of which are important to ensuring quality in healthcare services. This includes adolescents' health literacy, community support, appropriate package of services, provider competencies, facility characteristics, equity and non-discrimination, data and quality improvement, and adolescent participation[14]. We used *the Lancet Global Health Commission for High Quality Health Systems*[9] conceptual framework to choose three of the WHO quality standards. We focussed on foundations of high-quality health systems, as adolescent knowledge helps define how health services respond to adolescents. We also focussed on competent care and systems within processes of care, or evidence-based, patient-centred care.[9]

i. *Adolescents' health literacy* whereby adolescents know about mental health and their own mental health (through the health facility), as well as knowing where health services are located and when to go.[14]
ii. *An appropriate package of services* in which the health facility meets the needs of the adolescent by providing evidence-based information, counselling, correct diagnoses, treatment, and care services.[14]
iii. *Providers' competencies*, whereby healthcare providers are competent and provide effective care to adolescents (including respecting, protecting, and fulfilling the rights of adolescents).[14]

For peer review only

Box 1. Definitions of Quality by Organisation and Criteria

Organisations	Definition	Criteria
Lancet Global Health Commission for High Quality Health Systems (2018)	Quality health systems ensure that healthcare is optimised. This occurs by responding to population needs, providing care that maintains or improves health outcomes, and ensuring that all individuals feel valued	Quality framework components<br>• *Quality impacts*: better health, confidence in system, and economic benefit<br>• *Processes of care*: competent care and systems, positive user experience<br>• *Foundations*: population, governance, platforms, workforce, tools
WHO, OECD, World Bank (2018)	How health services ensure desired health outcomes for the population, using evidence-based knowledge	Foundations critical to high quality health services:<br>• *Healthcare workers*: motivated and supported<br>• *Healthcare facilities*: accessible and well-equipped<br>• *Medicines/Devices/Technologies*: safe in design and use<br>• *Information Systems*: continual monitoring<br>• *Financing mechanisms*: enable and encourage quality
National Academies of Sciences (2018)		Six dimensions of quality<br>• Safety<br>• Effectiveness<br>• Person-centeredness<br>• Accessibility, Timeliness, Affordability<br>• Efficiency<br>• Equity

Sources: Kruk ME, Gage AD, Arsenault C, et al. High-quality health systems in the Sustainable Development Goals ear: time for a revolution. *Lancet Glob Health* 2018;6(11):e1196-e252; WHO, OECD, WB. Delivering Quality Health Services: A Global Imperative for Universal Health Coverage. Geneva: World Health Organization 2018; IOM. Crossing the Quality Chasm: A New Health System for the 21st Century. Washington(DC): National Academies Press(US).

Despite a growing interest and investment in adolescent mental healthcare services, and the quality of such services, evidence on care quality and its effectiveness remain limited.[9] There is a paucity of information on how quality mental health services for adolescents should be developed and organised, clinicians trained, and health facility interventions implemented to improve adolescents' mental health outcomes and meet their needs.[15-17] The objective of this systematic review is to evaluate the quality of adolescent mental health service provision globally, according to the WHO Global Standards of adolescent mental health literacy, provider competencies, and appropriate package of services.[14] This review

For peer review only - http://bmjopen.bmj.com/site/about/guidelines.xhtml

focusses on quantitative and mixed-method evaluations of mental health services for adolescents (10-19 years) with suspected or diagnosed cases of depression, anxiety, and PTSD.

METHODS

The protocol was registered with PROSPERO (CRD42020161318) and is available online (https://www.crd.york.ac.uk/prospero/display_record.php?RecordID=161318). For this systematic review, we used the WHO Global standards for quality health care services for adolescents, focussing on standards for mental health services.[14]

Selection criteria

Inclusion criteria were quantitative and mixed-method studies that evaluated or assessed quality measures as defined by the WHO Global Standards (adolescent mental health literacy, provider competencies, and appropriate package of services) applied to existing mental health services targeted to adolescents (10-19 years) for depression, anxiety, and PTSD. We chose to focus on depression, anxiety, and PTSD as these are some of the most common mental health outcomes associated with living in challenging environments. [18-20, 21-23] Also, there is little evidence on the quality of services delivered to adolescents experiencing these mental health conditions. Articles that focussed on adolescent mental health literacy, appropriate package of services, and provider competencies were identified and classified according to the process and output criteria in the WHO report found in supplemental material 1.[14] Adolescents included were those who have used or are currently using mental health services, or are exposed to interventions or strategies within established mental health services. Mental health services were defined as health services delivered at the primary, secondary, or higher health facility level that offered prevention and treatment for anxiety, depression, and PTSD, and community-based initiatives originating from these health service levels. Healthcare providers delivering mental health services to adolescents were included. No exclusions were made on country. In cases where studies included individuals aged 18-24 years or 0-10 years, inclusion criteria were met as long as adolescents 10-19 years were the primary population of the study.

School-based health services or linkages to school-based health services and interventions were excluded as we focussed on services delivered in healthcare facilities. Transition services for adolescents to adult services were excluded as we strictly focussed on the adolescent age group. Clinical interventions that focussed solely on improving health outcomes were also excluded if they did not detail ways that quality measures were used.

For peer review only - http://bmjopen.bmj.com/site/about/guidelines.xhtml

Mental health services linked to a comorbid physical health condition or learning disability were excluded as we focussed specifically on depression, anxiety, and PTSD as primary outcomes. Private healthcare services and family therapy were also excluded. Given the launch of the *Mental health Gap Action Programme (mhGAP)* guidelines by the WHO in 2008[24] which highlights the large treatment gap globally, studies prior to 2008 were excluded.[25] No language restrictions were applied.

Search Strategy

The Preferred Reporting Items for Systematic Reviews and Meta-Analyses (PRISMA) methodology was used to select the articles.[26] Peer-reviewed literature was searched through the following databases: Pubmed, PsycINFO, MEDLINE, EMBASE, and LILACS from 1 January 2008 to 31 December 2019. Articles were also found by screening references in selected scientific articles that matched eligibility for inclusion. The search strategy is provided in Table 2.

Table 2. Search strategy of electronic databases

Evaluation terms	Quality terms	Population	Setting
Evaluation	Quality	Adolescent	Mental health service
Assessment	Health literacy	Youth	Primary mental health service
	Appropriate package of services	Teen	Mental health counselling
	Provider competencies	Young People	GP service

Data Collection and Extraction

Titles and abstracts were exported to Endnote[27] and scanned for relevance. Articles were removed if they did not meet inclusion criteria or were duplicates. The full text of included articles was obtained and studies were classified in: (i) mental health literacy; (ii) appropriate package of services; or (iii) provider competencies. Two authors (MQD and KJR) assessed the studies to ensure that they met the inclusion criteria for this review.

Data Synthesis and Quality Assessment

A narrative synthesis of the included studies was undertaken as the lack of homogeneity precluded a quantitative synthesis of findings. The methodological quality of the studies was assessed using the National Institutes for Health (NIH) Study Quality Assessment Tools.[28]

For peer review only - http://bmjopen.bmj.com/site/about/guidelines.xhtml

Studies were assessed for sources of bias (e.g., patient selection, performance, attrition, and detection), confounding, study power, and strength of causality in the association between interventions and outcomes.[28] Two reviewers rated each study independently (MQD and KJR). The quality assessments were compared and disagreements resolved through discussion with a third reviewer. Based on the ratings of each component, each study received an overall rating of good, fair, poor. Extracted data were entered into a table of study characteristics, including the quality assessment ratings for each study (Table 3).

Patient and public involvement

Patients and the public were not involved.

RESULTS

Study Selection

Figure 1 shows the results of the search and selection strategy. Of 19,586 references identified, 433 full-text articles met inclusion criteria from which a total of 17 articles were included in the study.

Figure 1. Flow diagram of studies

For peer review only

For peer review only - http://bmjopen.bmj.com/site/about/guidelines.xhtml

Table 3. Characteristics of included studies

Author	Type of mental health service	Country	Service, Intervention, Study description	Study/Evaluation design	Target population	Element(s) of quality addressed	Results	Quality assessment
Davidson et al (2019)	Secondary level services	USA	Tablet trauma-focussed cognitive behavioural therapy (TF-CBT) included 11 activities that focussed on breathing retraining, muscle relaxation, affective regulation and cognitive coping; other activities are specific to the TF-CBT model, including psychoeducation relating to traumatic events, safety education, and gradual exposure.	Pilot randomized controlled trial (RCT). Participants were randomised to Tablet TF-CBT and usual care. The Working Alliance Inventory was used to measure therapeutic alliance and patient satisfaction was measured using the Child/Adolescent Satisfaction Questionnaire	n=18 providers<br><br>n=32 children and adolescents (5-16 years, mean=11.5 years)	Appropriate package of services<br><br>Provider competency	Strong alliance between adolescents and providers, d=0.11<br><br>Assisted in skills-based learning, d= 0.47<br><br>Adolescent satisfaction with treatment (Child/Adolescent Satisfaction Questionnaire), d=0.53	Fair

Jager et al (2017)	Secondary level services	The Netherlands	Patient-centred communication through Take Care study	Longitudinal prospective cohort over the course of a year at three different time points using the Strengths and Difficulties Questionnaire	n=315 12-18 year olds	Appropriate package of services<br><br>Provider competencies	Lower reduction of psychosocial problems when adolescents valued communication but didn't experience it during the therapy sessions (total difficulties score at T1 an average of 15.6 and at T2 13.9)<br><br>Adolescents whose communication needs and experiences matched, had a greater reduction in mental health issues (total difficulties score at T1 15.7 and at T2 12.8)	Fair
Ougrin et a (2018)	Tertiary level services	UK	Supported Discharge Service Versus Inpatient Care Evaluation (SITE) project comprised of intensive	RCT Participants were randomised to SITE and usual care	n=53 in intervention arm (0-17 years; mean=16 years); n=53 in control arm (0-17 years;	Appropriate package of services	In unadjusted analysis, lower occupied bed days between intervention and control arm (median 34 days, p=0.04)	Good

For peer review only - http://bmjopen.bmj.com/site/about/guidelines.xhtml

			community-based treatment, which involved customised care plans of intensive case management, community/home treatment, day care in hospital		mean=16 years)		Intervention arm more effective and financially reasonable in comparison to control arm (the incremental cost effectiveness ratio was -£991)	
Dion et al (2010)	Emergency department (ED) services	Canada	Crisis Intervention Program that investigated the capability of emergency department staff to provide emergency assessments to adolescents with mental health issues presenting to the emergency department	Cross-sectional surveyed 87 emergency department medical staff (nurses, residents, physicians) about their experiences with the Crisis Intervention Program, their confidence in treating patients (managing, triaging, and referring) with mental health issues, and the benefits and challenges to the program.	n=87 medical staff (nurses, residents, and physicians)	Provider competency	ED staff reported greater confidence in managing and referring patients based on years of employment ($r=0.35$, $p<0.01$)<br><br>Physicians (83%) were more confident than nurses (8%) or residents (8%) ($p=0.05$)<br><br>The majority of emergency department staff (67% of nurses, 64% of residents, and 83% of physicians) were satisfied with the	Fair

For peer review only

							program	
Spenser et al (2009)	Tertiary level services	Canada	CHAT project which aimed to increase collaboration between community paediatricians and mental health clinicians: hired and integrated paediatrician within mental health team; surveyed clinicians about perceived needs; and provided continuing education based on identified needs	Cohort study	n=27 community paediatrician s; n=16 outpatient mental health clinicians	Provider competency	5 mental health clinicians stated that having paediatricians on mental health team was positive<br><br>Over 20% of clinical activity for almost 12 paediatricians deals with mental health issues<br><br>7 mental health clinicians stated that the educational sessions led to increased knowledge about mental health of children and adolescents	Poor
Ayton et al (2013)	Secondary level services	UK	Audit Care Programme Approach (CPA) care plans within Child and adolescent mental health services (CAMHS) and	Mixed methods including survey with consultants and review of case notes.	n=23 CAMHS consultants surveyed; 33 case notes reviewed	Appropriate package of services<br><br>Provider competencie s	CPA care plans audited assessed 96.4% of mental health and needs and 71.4% of family needs; other needs (housing: 33.9%, sexual health: 7.1%, and sign of	Fair

			identify service user involvement in all aspects of mental healthcare<br><br>This includes involving them in a needs assessment, developing and reviewing the care plan; and sharing responsibilities between the patient, carers, and relevant professional agencies.				relapse or crisis: 28.6%) minimally assessed in CPA plans	
Aupont et al (2013)	Primary level services	USA	Targeted Child Psychiatric Services that implemented a care model that facilitates adolescent's access to specialised mental health services, investigating whether the adolescent	Prospective cohort with adolescent service users	n=329 (m=12.3 years)	Appropriate package of services	The relative risks of staying in mental healthcare (instead of going back to paediatrics) was 7.5 for those with depression and 5.1 for those with anxiety disorders<br><br>the return rates to the referring paediatrician were	Fair

For peer review only

			remains in specialised mental health services or returns to paediatric primary care				27.9% and 5.9% for adolescents with anxiety and major depressive disorders	
Simmons et al (2016)	Enhanced primary level services	Australia	*headspace* implemented an online decision aid for adolescent and youth depression was developed. The aim of the intervention was help young people make evidence-informed decisions about their treatment, make them feel involved, adhere to treatment and reduce symptoms, and increase satisfaction	prospective cohort study with pre-decision, immediate post-decision, and follow-up measurements	n=57 12-25 year olds used the decision aid and completed the post-decision assessment; n=48 completed the follow-up assessment	Mental health literacy<br><br>Provider competencies	97% reported increased confidence in deciding about their own healthcare after using the decision aid (p=0.022)<br><br>On the Shared Decision Making Questionnaire, adolescents scored average of 37.4 (range 29-44); indicating high level of perceived involvement in the treatment process	Fair

For peer review only - http://bmjopen.bmj.com/site/about/guidelines.xhtml

Jager et al (2014)	Secondary level services	The Netherlands	In the Take Care study, Client-centred communication (measured using the Consumer Quality Index) affects adolescents participation and learning in their mental healthcare	Longitudinal, prospective cohort study	n=211 12-18 year olds	Appropriate package of services<br><br>Provider competencies	Adolescents who valued patient centred communication but did not have their communications needs met were less likely to adhere to their treatment (OR: 2.8; CI: 1.1-6.8)	Fair
Stevens et al (2009)	Tertiary level services	USA	Less intensive Telephone support services (TSS) intervention focussed on services and not symptoms	RCT to measure whether there's an increase service utilisation with TSS (defined as three brief telephone calls of case management and motivational interviewing) versus usual care plus (defined as three brief calls to ensure the researchers had the most recent contact information for participants)	n=179 11-20 year olds	Appropriate package of services	No significant differences found between treatment and control arms	Good

For peer review only - http://bmjopen.bmj.com/site/about/guidelines.xhtml

Anderson et al (2012)	Secondary level services	Australia	Body signs, Relaxation, Active helpful thoughts, Victory you're your fears, Enjoy! Reward yourself (BRAVE) for Teenagers-Online project:<br><br>Develop working alliance during online cognitive behaviour therapy (CBT) treatment for youth anxiety.	RCT comparing online versus face-to-face (clinic) delivery of CBT.<br><br>The researchers measured therapeutic alliance using the Working Alliance Inventory-Short Form.	n=73 12-18 year olds	Appropriate package of services	Adolescents in intervention (mean 5.77, SD 1.2) and control (mean 5.58, SD 1.34) reported strong working alliance<br><br>Adolescent working alliance was positively associated with compliance at 6 month follow-up (r=0.30, p<0.001)	Poor
Kapp et al (2017)	Secondary level services	Switzerland	Identifying what influenced perceived quality of care (therapeutic alliance) in CAMHS	Cross-sectional Therapeutic alliance was measured using an adapted version of the Helping Alliance Questionnaire, while satisfaction was measured using the Consumer Satisfaction Questionnaire	n=663 patients 10+years	Appropriate package of services	Patients who had time to formulate and ask questions had better alliance (p<0.001)<br><br>Easy accessibility to CAMHs by phone had higher alliance scores (p=0.037)	Fair

For peer review only - http://bmjopen.bmj.com/site/about/guidelines.xhtml

Cairns et al (2019)	Primary level services	Australia	In *headspace* services, influence of goal setting frequency and quality on patient retention	Cohort	n=283 clinical charts of 12-25 year olds	Appropriate package of services<br><br>Provider competencies	Emotional management and well-being goals were most frequently recorded<br><br>None of the analysed goals met criteria for being specific, measurable, and timed; 57% were specific while 14% were measurable; none had a timeframe<br><br>Goal quality was not associated with service retention	Fair
Ringle et al (2019)	Secondary level services	USA	The Resiliency and Disease Management initiative, or an evidence-based practice that includes Child and Adolescent Texas Recommended Assessment Guidelines, a	Medical record audit to investigate clinicians compliance with the Child and Adolescent Texas Recommended Assessment Guidelines	n=727 medical records of 8-18 year olds	Provider competencies	46% of children and adolescents received care that was guideline concordant<br><br>The most common service packages were those that focussed on skills training, therapy, and skills training	Fair

For peer review only

			comprehensive list of service packages that indicates level of service intensity and evidence-based practice (e.g., CBT)				+ wrap around services for internalising and externalising disorders; wrap around services and medication management for treatment foster care and aftercare services were the least recommended<br><br>Less intensive services were significantly more likely to be recommended than more intensive services ($p<0.001$)<br><br>Clients with worse functioning (*OR* = 0.985, p < .001), higher problem severity (*OR* = 1.02, p = .015), higher risk of harm to others (*OR* = 1.61, p < .001), more school problems (*OR* = 1.48, p <	

For peer review only

							.001), and who had a diagnosis of depression (*OR* = 1.37, p < .05) or a conduct-related disorder (*OR* = 1.37, p < .05) at intake were more likely to receive less intensive services than those re-commended by the Resiliency and Disease Management guidelines.	
Sattler et al (2019)	Mix of primary and secondary level	USA	Non-profit provider of medical and mental health services<br><br>evidence-based assessments	Medical record audit to investigate the frequency of clinical use of evidence-based assessments	n=694 medical records 7-17 year olds	Appropriate package of services<br><br>Provider competencie s	Patients received 1.48 evaluations on average for psychiatric symptoms<br><br>45.7% of all facilities used self-report measures and 5.2% used diagnostic interview<br><br>23.2% of psychologists documented the use of diagnostic	Fair

For peer review only - http://bmjopen.bmj.com/site/about/guidelines.xhtml

							interviews, compared to 2% and 0% of psychiatrists and primary care physicians (p<0.01)<br><br>43% psychologists, 43.3% psychiatrists, and 39.3% of social workers more likely to document specific diagnoses compared to primary care (22.6%) (p<0.01)<br><br>Self-report measures and diagnostic interviews more likely to result in specific diagnoses in comparison to those without (p<0.05)	
Sattler et al (2016)	Primary and secondary	USA	Non-profit provider of medical and	Medical record audit	n=801 7-17 year olds	Appropriate package of services	58.1% of patients did not receive a *specific* anxiety disorder diagnosis	Fair

For peer review only

	level services		mental health services<br><br>use of evidence-based assessment techniques, including structured interviews, rating scales, DSM-IV-TR and agreement at primary care facilities, general mental health clinics, and specialty clinics for anxiety disorders			Provider competencies	40.1% received care from primary care clinic, 44.7% had at least one visit with a mental health clinic, and 15.2% had at least one specialty visit<br><br>5.3% of anxiety disorder speciality clinics used structured diagnostic interviews; 21% of all health facilities used rating scales (28.9% of speciality clinics, 19.6% of general mental health clinics, and 15% primary care)<br><br>Evaluations in specialised clinics resulted in specific diagnosis (p<0.001); rating scales were associated with specific diagnosis (p=0.04)	

For peer review only

Higa-McMilan et al 2017)	Secondary level services	USA	Practices derived from the evidence base (PDEB)	Medical record audit with data extracted from the Child and Adolescent Mental Health Information System, using the Monthly Treatment and Progress Summary	n=2,485 3-19 year olds	Appropriate package of services<br><br>Provider competencies	55-93% of cases use the following PDEB for adolescents: cognitive, psychoeducational, relaxation, modelling; 99.7% of youth had at least one PDEB over their treatment course	Fair

Acronyms: Trauma focussed cognitive behavioural therapy (TF-CBT); randomized controlled trial (RCT); Supported Discharge Service Versus Inpatient Care Evaluation (SITE); emergency department (ED); Child and adolescent mental health services (CAMHS); Care programme approach (CPA); Telephone support service (TSS); Body signs, Relaxation, Active helpful thoughts, Victory over your fears, Enjoy! Reward yourself (BRAVE); The Choice and Partnership Approach (CAPA); Practices derived from the evidence base (PDEB); Managing and Adapting Practice (MAP); Practices derived from the evidence base (PDEB)

Sources: Davidson TM, Bunnell BE, Saunders BE, et al. Pilot evaluation of a tablet-based application to improve quality of care in child mental health treatment. Behavior Therapy 2018:No Pagination Specified; Jager M, Reijneveld SA, Almansa J, et al. Less reduction of psychosocial problems among adolescents with unmet communication needs. European Child & Adolescent Psychiatry 2017;26(4):403-12. doi: 10.1007/s00787-016-0901-y; Ougrin D, Corrigall R, Poole J, et al. Comparison of effectiveness and cost-effectiveness of an intensive community supported discharge service versus treatment as usual for adolescents with psychiatric emergencies: A randomised controlled trial. The Lancet Psychiatry 2018;5(6):477-85; Dion J, Kennedy A, Cloutier P, et al. Evaluating crisis intervention services for youth within an emergency department: A view from within. Child Care in Practice 2010;16(3):241-56; Spenser HR, Gillies A, Maysenhoelder H. The CHAT project: Paediatricians and mental health clinicians: Working together for the sake of the children. Journal of the Canadian Academy of Child and Adolescent Psychiatry 2009;18(2):110-16; Ayton A, Meads G. The implementation of the care programme approach in the west midlands CAMHS: The impact of organisational diversity on patient safety - an extended audit. International Journal of Clinical Leadership 2013;17(4):185-200; Aupont O, Doerfler L, Connor DF, et al. A collaborative care model to improve access to pediatric mental health services. Administration and policy in mental health 2013;40(4):264-73. doi: 10.1007/s10488-012-0413-0 [published Online First: 2012/04/25]; Simmons MB, Elmes A, McKenzie JE, et al. Right choice, right time: Evaluation of an online decision aid for youth depression. Health Expect 2017;20(4):714-23. doi: 10.1111/hex.12510 [published Online First: 2016/10/18]; Jager M, Reijneveld SA, Metselaar J, et al. Discrepancies between adolescents' attributed relevance and experiences regarding communication are associated with poorer client participation and learning processes in psychosocial care. Patient education and counseling 2014;97(3):332-8. doi: 10.1016/j.pec.2014.08.020 [published Online First: 2014/09/17]; Stevens J, Klima J, Chisolm D, et al. A trial of telephone services to increase adolescent utilization of health care for psychosocial problems. J Adolesc Health 2009;45(6):564-70. doi: 10.1016/j.jadohealth.2009.04.003 [published Online First: 2009/11/26]; Anderson RE, Spence SH, Donovan CL, et al. Working alliance in online cognitive behavior therapy for anxiety disorders in youth: comparison with clinic delivery and its role in predicting outcome. J Med Internet Res 2012;14(3):e88. doi: 10.2196/jmir.1848 [published Online First: 2012/07/14]; Kapp C, Perlini T, Jeanneret T, et al. Identifying the determinants of perceived quality in outpatient child and adolescent mental health services from the perspectives of parents and patients. European Child & Adolescent Psychiatry 2017;26(10):1269-77. doi: 10.1007/s00787-017-0985-z; Cairns AJ,

For peer review only - http://bmjopen.bmj.com/site/about/guidelines.xhtml

Kavanagh DJ, Dark F, et al. Goal setting improves retention in youth mental health: a cross-sectional analysis. Child & Adolescent Psychiatry & Mental Health [Electronic Resource] 2019;13:31; Mora Ringle VA, Hickey JS, Jensen-Doss A. Patterns and predictors of compliance with utilization management guidelines supporting a state policy to improve the quality of youth mental health services. Child Youth Serv Rev 2019;96:194-203. doi: 10.1016/j.childyouth.2018.11.035 [published Online First: 11/16]; Sattler AF, Leffler JM, Harrison NL, et al. The quality of assessments for childhood psychopathology within a regional medical center. Psychological Services 2019;16(4):596-604; Sattler AF, Ale CM, Nguyen K, et al. Use of evidence-based assessments for childhood anxiety disorders within a regional medical system. Psychol Serv 2016;13(4):411-18. doi: 10.1037/ser0000091 [published Online First: 2016/11/01]; Higa-McMillan C, Kotte A, Jackson D, et al. Overlapping and Non-overlapping Practices in Usual and Evidence-Based Care for Youth Anxiety. The Journal of Behavioral Health Services & Research 2017;44(4):684-94. doi: 10.1007/s11414-016-9502-2.

For peer review only

Study Characteristics and Settings

All 17 studies were conducted in high-income countries: in the USA (7 studies),[29-35] Australia (3 studies),[36-38] United Kingdom (2 studies),[39, 40] Canada (2 studies),[41, 42] the Netherlands (2 studies),[43, 44] and Switzerland (1 study).[45] Most of the studies focussed on evaluating or assessing the provision of services that are appropriate for adolescents (13 studies), followed by enhancing provider competencies (12 studies), and increasing adolescents' mental health literacy (1 study).

The services ranged from emergency,[41] to primary level;[30, 32, 33, 36, 38] secondary level mental health services;[29, 31-33, 35, 37, 39, 43-45] and tertiary level services.[34, 40, 42]

Quality Assessment

The majority of the studies were assessed as 'fair' quality (n= 13, 76.5%); 2 (11.8%) studies were assed as 'good' quality, and 2 (11.8%) were assessed as 'poor quality'. Studies rated as 'poor' mainly had higher bias, lack of clarity on methods and outcomes analysed, and confounding. Ougrin and colleagues (2018) and Stevens and colleagues (2009) implemented studies that were rated as good quality.[34, 40] Both conducted randomised controlled-trials in tertiary level facilities. They also experienced low drop-out rates, high adherence to the interventions, and consistently used valid and reliable measures.[34, 40]

Conceptualising Quality

The majority of studies did not conceptualise quality.[30, 34, 36, 37, 39-44] Where it was conceptualised, it was in reference to high quality care, defined as healthcare provider fidelity to evidence-based treatment models and adolescents' engagement in the treatment process (satisfaction and quality of engagement with therapists and adolescents);[29] or as quality indicators in child and adolescent mental health services, specifically around patient satisfaction and quality engagement between the therapist and adolescent.[45] Common themes within these studies were a focus on the processes of care and quality impacts (improved mental health and greater confidence of the health service and system).[9]

Other studies mentioned quality in relation to patient-centred communication or how providers adapt their communication style to meet the needs and preferences of their patients.[44] Quality was also considered in relation to goal setting between the therapist and adolescent patient and whether these goals were specific, measurable, achievable, realistic/relevant, and timed.[38] The use of evidence-based assessments, practices, and

For peer review only

policies was another aspect of quality mentioned in some studies[32, 33, 35]. Other aspects of quality linked to communication were: coordination of care between an in-patient mental health unit and a community service;[39] mental health care delivered through the emergency department;[41] and collaboration between family physicians and psychiatrists.[42] Jager and colleagues (2017) measured affective quality as a key component of patient-centred communication, however, it was not defined.[43] The common theme that emerged from these studies is that they focussed on foundations of quality care.[9] Findings from the review are presented below by WHO quality standards.[14]

Quality Standards

Adolescent Mental Health Literacy

One study reported outcomes relevant to adolescent mental health literacy, which focussed on an online decision aid for mental health services.[36] 97% of adolescents reported increased confidence and awareness in deciding about their own healthcare and involvement in the treatment process when exposed to an online decision aid (p=0.022). The quality of evidence from this study was fair.

Appropriate Package of Services

Thirteen of the 17 studies evaluated services that met the WHO standard for appropriate packages of services.[14] Interventions that involved aspects of an appropriate package of mental health services were diverse and targeted the quality of engagement between the therapist and adolescent [29, 37, 38, 45] patient-centred communication,[43, 44] mental health service use,[34] linkages to mental health services,[30] health facility culture and patient safety,[39] clinician's assessment of diagnostic and treatment services,[32, 33] and intensive community treatment.[40] The quality of the evidence for the 13 studies was poor to good.

Four of the 13 studies[29, 37, 38, 45] reported improvements in the quality of engagement between therapist and adolescent patient, including the interaction, collaboration, and bond[37]. These included the use of a tablet-based application on trauma-focused cognitive behavioural therapy (TF-CBT) in the US,[29] an online cognitive behaviour therapy intervention in Australia,[37] an outpatient child and adolescent mental health clinic in Switzerland,[45] and the use of goal setting in Australia.[38] Davidson and colleagues (2018) evaluated the tablet-based application on TF-CBT finding a small to medium effect size on

For peer review only - http://bmjopen.bmj.com/site/about/guidelines.xhtml

developing therapeutic tasks (d=0.47 and a small effect size on therapeutic bond (d=0.11), with most adolescents satisfied with the intervention (d=0.53).[29] Anderson and colleagues (2012) evaluated an online cognitive behaviour therapy intervention with minimal therapist contact finding that greater therapeutic alliance led to greater adolescent adherence with treatment at 6 month follow-up (r=0.30, p<0.001). Results also found that adolescents in both the intervention and control arm reported strong therapeutic alliance.[37]

Two of the 13 studies[43, 44] focussed on patient-centred communication in the Netherlands, finding that adolescents who did not experience patient-centred communication were less likely to adhere (OR: 2.8, CI: 1.1-6.8) and have confidence (OR: 4.5, CI: 1.8-11.6) in their course of treatment.[44] They also were less likely to experience a significant reduction in their mental health problems, compared to those who experienced patient-centred communication.[43]

One study evaluated mental health service use through a telephone support service (TSS) intervention in the US.[34] The study found no difference in mental healthcare utilisation (p=0.65) between adolescents in the intervention and those in usual care.[34] Another study evaluated referrals between paediatric care to mental health services through the Targeted Child Psychiatric Service (TCPS) program in the US. The program enables access to specialised mental health services for adolescents with mental health conditions from paediatric primary care, ensuring long-term management in the most appropriate healthcare setting. Results showed that adolescents with depression and anxiety required continued access to specialised mental healthcare.[30]

One study[39] evaluated health facility culture and patient safety with the Care Programme Approach (CPA), which ensures that children and adolescent patients are involved in all aspects of their mental health care in the UK. Patient safety was found to be an issue. In fact, unplanned discharge (i.e., self-discharge, tribunal discharge, or commissioning pressure to discharge) was the most common problem due to siloed rather than collaborative, decision-making. Other challenges were limited collaboration between early intervention, education, and Child and Adolescent Mental Health Services (CAMHS) teams; and a lack of joint protocols on the CPA and discharge between organisations.[39]

Two studies in the US focussed on the use of structured interviews, symptom rating scales, and Diagnostic and Statistical Manual of Mental Disorders (DSM-IV-TR) diagnostic criteria in primary, secondary, and tertiary level services.[32, 33] Results showed that structured

For peer review only - http://bmjopen.bmj.com/site/about/guidelines.xhtml

For peer review only

diagnostic interviews were more likely to be used by psychiatrists and psychologists compared to general physicians ($p<0.01$).[33]

One study examined whether a Supported Discharge Service (SDS), or intensive community treatment, would be more beneficial and cost-effective than usual care among adolescents.[40] A significant difference in overall number of occupied bed-days from the SDS arm was found at 6 months (median 34 days, $p=0.04$) compared to usual care. The SDS was found to have at least 50% probability of being cost-effective in comparison to usual care and willingness to pay for outcome improvements (the incremental cost effectiveness ratio was -£991), or the cost per life year gained.[40]

Providers' Competencies

Twelve studies included outcomes relevant to healthcare provider competency. They focussed on confidence in managing and referring adolescents with mental health issues,[41] greater collaboration between paediatricians and mental health clinicians,[42] use of evidence-based practices,[31-33, 35] use of a tablet to facilitate adolescent patient engagement in therapy,[29] provision of information,[36, 43, 44] and implementation of care models and plans.[30, 38, 39] The quality of evidence for the twelve included studies ranged from poor to fair.

In evaluating confidence of referring adolescents with mental health patients, Dion and colleagues (2010) found that training through a Crisis Intervention Program increased emergency department's staff confidence in managing and triaging patients ($r=0.35$, $p<0.01$) in Canada.[41] Another study evaluated collaboration between paediatricians and outpatient mental health clinicians in Canada. Results showed a positive effect on patient care with a paediatrician on the mental health team.[42]

Four studies evaluated the use of evidence-based practices and guidelines in adolescent mental health services.[31-33, 35] Higa-McMillan and colleagues (2017) found that the most commonly used evidence-based practices for adolescents with anxiety disorders included cognitive, psychoeducational, relaxation, and modelling.[31] In an US study, the authors found that patients were more likely to receive less intensive services if they had poorer functioning; greater problem severity; greater risk of harm to others, greater school problems, with a diagnosis of depression or conduct disorder than guideline recommended.[35]

For peer review only - http://bmjopen.bmj.com/site/about/guidelines.xhtml

Two studies in the Netherlands found that adolescents who did not experience patient-centred communication were less likely to understand (OR: 3.7, CI: 1.5-9.0) [43 44](OR: 3.1, 1.1-8.5) [44] their course of treatment. Similarly, through the use of an online decision aid, 93% of participants were more likely to make a healthcare decision that was guideline concordant (p=0.004) and one that they preferred.[36]

DISCUSSION

To our knowledge, this is the first review to evaluate quality of adolescent mental health services.[14] A total of 17 studies were identified, all from high-income countries: 13 studies focussed on packages of services; twelve on healthcare provider competency; and one on mental health literacy. There was some evidence that the interventions improved mental health, however, we cannot conclusively state they were effective. Most studies did not conceptualise quality.

Despite the large contribution of mental health conditions to adolescents' global burden of disease and the need for quality mental healthcare services, we found that most studies lacked conceptualisation of quality, and did not have a clear framework or definition of quality. There were a variety of instruments used to measure quality and its indicators. Our understanding of quality in mental health services, as well as the generalisability of our findings, therefore, is limited. Our findings also indicate a large service gap and suggests that there is a need to not only develop and standardise a definition of what constitutes quality adolescent mental healthcare, but also develop and standardise methods that measure quality in adolescent mental healthcare. We recognise that the WHO Global Standards quality framework is one way of categorising quality and we may have missed other frameworks. Leslie and colleagues (2018) warn that despite the recent initiatives and focus on quality in healthcare, the various concepts and frameworks used to define and measure quality leads to "inconsistent assessments and incomparable investments, leaving researchers and policymakers without direction".[46]

There are several challenges to implementing and providing quality within adolescent mental health services, including stigma, the lack of adolescent mental health capacity and professional expertise, and the current disease-based model of medicine. Also, quality in mental health services has received little attention in relationship to adolescents.

Stigma is a significant barrier to the availability and delivery of quality mental health services within communities.[13] It has been posited that stigma occurs at the structural

For peer review only - http://bmjopen.bmj.com/site/about/guidelines.xhtml

(organisation, resources, quality standards), interpersonal (the quality of engagement between the healthcare provider and adolescent, patient safety), and intraindividual levels (healthcare providers unwilling to assess adolescent mental health conditions, adolescents unwilling to seek mental healthcare services).[47, 48] This failure in quality prevents adolescents from seeking and continuing care for mental health conditions due to perceived stigma. [47, 49, 50, 51, 52, 53] It points to a need for mental health literacy among healthcare providers. Quality mental health services cannot be achieved without healthcare providers having a reasonable understanding of adolescent mental health. This needs to start with healthcare provider training and preservice education.[49]

All of the studies were from high-income countries, illustrating an important gap in the literature on quality of adolescent mental healthcare in low- and middle- income countries (LMICs). This could be due to the conceptualisation of mental health in LMICs at the national and local levels[13] as well as barriers to service demand. It has been posited that this could be attributed to the way heath systems are set up in LMICs, focussing on acute medical conditions rather than complex conditions that require long-term care, such as mental health conditions.[9, 13] Patel et al (2019) argue that the multiple aspects of mental health conditions do not follow the typical disease-based model of medicine, and that a ‘one size fits all’ approach does not work.[13] Particularly when some mental health conditions do not easily fit within diagnostic-led services and adolescents have subsyndromal conditions.[53] This affects quality in adolescent mental health services as treatment and care plans should be developed with the adolescent patient, tailored to meet their mental health needs.

There have been efforts to overcome these challenges, as identified in table 4.

For peer review only

For peer review only - http://bmjopen.bmj.com/site/about/guidelines.xhtml

Table 4. Frameworks to Address Quality in Adolescent Health Services

Reference	Framework	Components of Framework
Sawyer et al (2014)	Conceptual framework for adolescent-friendly healthcare based on experience of care and evidence-informed care, including a set of 14 Indicators of quality healthcare for adolescents in hospitals	*Experience of care:*
		Felt welcome in hospital
		Age appropriate environment
		Respected by clinicians
		Trust in clinicians
		Understanding of health information
		Involvement in decisions about care or treatment
		Comfort asking questions about health and wellbeing
		Evidence informed care:
		Psychosocial assessment
		Confidentiality discussions
		Time alone in consultations
		Self-management
		Transfer to adult services
		Supported to continue education
		Connection to external supports
Ambresin et al (2013)	Domains for youth-friendly care to assess how well services are engaging young people	Accessibility of healthcare
		Staff attitude
		Communication
		Medical competency
		Guideline-driven care
		Age-appropriate environment
		Involvement in healthcare
		Health outcomes
UK NHS (2007)	'You're Welcome' quality criteria to ensure that health services (primary, community, specialist and acute)are young people-friendly	Accessibility
		Publicity
		Confidentiality and consent
		The environment
		Staff training, skills, attitudes and values
		Joined-up working
		Monitoring and evaluation and involvement of young people
		Health issues for adolescents
		Sexual and reproductive health service
		Child and adolescent mental health services
Sayal et al (2012)	10 quality standards for children and adolescents in primary mental healthcare	Confidentiality
		Knowledge
		Awareness
		Communication
		Continuity of care
		Access and referral

Sources: Sawyer SM, Ambresin AE, Bennett KE, et al. A measurement framework for quality health care for adolescents in hospital. J Adolesc Health 2014;55(4):484-90.; Ambresin A-E, Bennett K, Patton GC, et al. Assessment of youth-friendly health care: a systematic review of indicators drawn from young people's perspectives. The Journal of adolescent health: official publication of the Society for Adolescent Medicine 2013;52(6):670-81; NHS. You're Welcome Quality Criteria: Making health services young people friendly. London: NHS, 2007; Sayal K, Amarasinghe M, Robotham S, et al. Quality standards for child and adolescent mental health in primary care. *BMC Fam Pract* 2012;13:51-51.

For peer review only - http://bmjopen.bmj.com/site/about/guidelines.xhtml

In reviewing the above frameworks, a good starting point for measuring quality in adolescent mental healthcare services would be a more scalable combination of the youth-friendly guideline driven care developed by Ambresin and colleagues (2013) and the quality standards developed by Sayal and colleagues (2012). The same investments that promote quality in other age groups will be valuable for adolescents, but greater specificity and focus is required around the health service aspects of engagement, communication, and confidentiality. The studies identified in our review examined aspects of engagement and communication, but did not explore confidentiality.

Findings from our review should be taken in light of the broader challenges to quality in adolescent mental health services, as described above. With adolescent mental health literacy, we found that a youth decision aid helped young people make evidence-informed decisions about their treatment, feel engaged in the process, and increase treatment adherence.[36] Previous literature has found similar results, with patients reporting increased involvement in treatment decision-making, increased knowledge about the treatment options and outcomes, and comfortability with making decisions.[54] As a foundation for quality, adolescents' knowledge shapes the way mental health services respond to them, helping adolescents hold these services to account.[9] For packages of services, the quality of engagement between the adolescent and therapist was found to lead to positive outcomes.[55, 56] The quality of the patient-therapist relationship has led to greater treatment efficacy,[57] increased autonomy, patient alliance and engagement, and greater favourable outcomes.[37, 58] It ensures that the adolescent's perspective is included, that they consent and assent to their treatment plan,[59] and that they can address problems throughout the treatment process.[60] Evidence from the UK has shown that current services are not adequate for young people's mental health needs,[61-63] with youth reporting that they should be more engaged in the design of mental health services. In an evaluation of 19 CAMHS services in the UK, almost half of these services did not offer age-appropriate mental healthcare and interventions.[64, 65] From the studies on provider competency, we found that training healthcare providers on adolescent mental health conditions helped build their confidence and knowledge when treating adolescents.[41, 42] This aligns with previous literature as healthcare providers reported confidence, knowledge, and a lack of specialised providers as barriers to care.[66, 67] Provider competency is a foundation and process of care.[9] Adolescent mental healthcare providers require adequate clinical education and training on adolescent mental health. They also should provide evidence-based treatment, communicate clearly, ensure confidentiality and autonomy, promote timely and effective care and instil confidence in their adolescent patients that their conditions are being correctly detected and managed.[9]

For peer review only - http://bmjopen.bmj.com/site/about/guidelines.xhtml

We assessed the evidence using the NIH Quality Assessment Tool. The quality of the evidence ranged from poor to good in relation to the quality standards set out by this review. It should be noted that upon assessment of the studies included in the review, there were various study design limitations, which could bias the results of the review. Furthermore, evaluations and the content of the service varied, as well as the sample size and tools used to evaluate the services. Some studies did not mention the type of evaluation they used. Also, it is not possible from the current review to identify the most effective standard of quality or service delivery method, as conforms to the WHO global standards.[14]

For peer review only

For peer review only - http://bmjopen.bmj.com/site/about/guidelines.xhtml

Box 2. Recommendations

We have several recommendations to further research on quality in adolescent mental health services and mental health outcomes. First, it is important to develop and standardise a definition of quality and the methods that can be used to measure quality in adolescent mental health services. Methods for measuring quality should be feasible and adaptable to various contexts (urban, rural). Achieving this would ensure comparability across studies, a greater understanding of quality in adolescent mental healthcare services, and the identification of gaps in knowledge.

Second, coordinated and collaborative efforts are needed to strengthen the evidence on quality in adolescent mental health services. This includes research on adolescent mental health needs, the services that are best able to respond to their needs, as well as effective strategies to improve the quality of mental health services, including training and continued education for healthcare providers on adolescent mental health.[68] Adolescents should be actively engaged in this process. At the same time, we need evidence on the sustainability and effectiveness of quality interventions in adolescent mental healthcare services to scale up through the health system. This requires regular data collection to monitor the quality of mental healthcare services (for example, implementation of evidence-based guidelines, quality of life) to provide feedback on mental health service performance and identify areas for improvement.

Furthermore, research needs to be conducted on how quality in mental healthcare services can respond to adolescents that are exposed to daily adversity, such as community violence, and how quality in mental healthcare services can be implemented and respond more effectively to their needs.

Third, health services need to proactively engage adolescents about mental health and the services available to them in order to identify and respond to the adolescent's mental health needs. This is particularly important for adolescents in vulnerable situations, who are less likely to access services because they do not have a supportive environment or transportation to attend those services. Similarly, a lack of communication between healthcare providers, adolescents, and referral services can lead to confusion about treatment with the adolescent falling through the health service gap when referred to another level.[53] In addition, confidentiality is key for adolescents as mental health services should provide a safe, supportive and empowering space for adolescents to seek and receive care, without the worrying that information will be shared with parents or guardians.[69]

Fourth, within the aims of this review, there needs to be greater investment in integrating adolescent mental health services into primary healthcare services. Our findings indicate that primary healthcare services were often used by adolescents for mental health services.

Promoting the integration of mental healthcare services into primary care could help reduce stigma and discrimination among adolescents, families, trained healthcare providers and the community. It could also help close the treatment gap between the prevalence of adolescent

For peer review only - http://bmjopen.bmj.com/site/about/guidelines.xhtml

Box 2. Recommendations (continued)

mental health conditions and the number of them receiving care.[70, 71] Mental health services offered through primary health care could respond to the unique needs of adolescents and youth, which include among others, increasing their mental health knowledge and addressing risky behaviours that arise during this stage of life.[20]

In fact, integrating mental health services into primary care can also help address the limited mental health resources in LMICs.[72] Our review found that task-shifting, or training non-specialised healthcare providers on adolescent mental health, was effective in building their capacity on adolescent mental health services. As recommended by the *Lancet Commission on global mental health and sustainable development*, integrating mental health services into primary care could also help address the limited mental health resources in LMICs.[72] At the same time, cost-effectiveness analyses and greater research on the financial savings associated with good adolescent mental health should be conducted at different health facility levels. This information can then be used to garner greater governmental investment in this area. At the policy level, we need to identify champions of adolescent mental health and quality of mental health services for this age group, so that they can push for policies and legislation.

Fifth, in looking at the broader challenges of quality and the extent that stigma negatively influences quality of adolescent mental health services, we recommend conducting research on adolescents', families, and the communities' social and cultural conceptualisations of mental health and mental health stigma, to address the limited knowledge available on cultural concepts, practices, and stigma. This should also extend to healthcare providers given the stigmatising attitudes and lack of respect experienced by adolescents with mental health conditions. This information could be used to inform anti-stigma interventions targeting healthcare providers, adolescents, families, communities about mental health. Within health services, we recommend incorporating stigma reduction indicators into quality of care measures, as put forth by Knaak and colleagues (2015).[73]

Study Limitations

This review should be interpreted within the context of a number of limitations. We recognise that there are various sources outside of health services within the community and schools, that provide information on adolescent mental health and services addressing mental health conditions. The scope of the review was limited to mental health services to adolescents 10-19 years of age, although children were sometimes included due to the nature of service delivery. We recognise that adolescent mental health transition services to adult services represent an important aspect of quality of care, with adolescents transitioning to adult services at different ages in both high-income and LMICs. We also investigated mental

For peer review only - http://bmjopen.bmj.com/site/about/guidelines.xhtml

health services for adolescents with suspected or diagnosed cases of depression, anxiety, and PTSD, not investigating cases with other mental health conditions. The quality criteria we used is open to subjective interpretation. The many studies and services that use an extended definition of adolescence[74] as up to 24 years; these were not included. The review did not look at parenting interventions, which have been found to improve the mental health of adolescents[75] or school-based mental health interventions, which have also been found to contribute to health services.[16] We tried to limit the services to public mental health services, however, it was hard to determine what public and private healthcare in certain settings was.

Conclusions

The review indicates that there was no standard consensus on quality in mental health services, and most of the identified studies did not conceptualise quality – highlighting a critical evidence gap regarding adolescent mental health services. There are several challenges that must be addressed as a way to begin to improve quality mental health care. Promising strategies include improving the quality of engagement between adolescent patients and therapists, as well as greater training among healthcare providers on adolescent mental health. Despite this, improving the quality of adolescent mental healthcare services requires further research and investment.

Funding statement. This research received no specific grant from any funding agency in the public, commercial or not-for-profit sectors. Kathryn J Roberts is supported by an Economic Social Research Council (ESRC) PhD studentship through the UBEL DTP (kr0001).

Competing Interests. The authors have nothing to disclose.

Contributors. MQD led the study design, data searches, data extraction, quality appraisal, synthesis and drafted the manuscript. KJR contributed to the design, data extraction, quality appraisal and writing of the manuscript. LK and DD provided significant and critical contribution to the conceptualisation, design and interpretation of findings. SS critically reviewed the manuscript and offered key intellectual input in the synthesis and interpretation of findings. RC critically reviewed the manuscript and offered important intellectual input.

For peer review only

References

1. Kessler RC, Amminger GP, Aguilar-Gaxiola S, et al. Age of onset of mental disorders: a review of recent literature. *Current Opinion in Psychiatry* 2007;20(4):359-64. doi: 10.1097/YCO.0b013e32816ebc8c
2. Jones PB. Adult mental health disorders and their age at onset. *Br J Psychiatry Suppl* 2013;54:s5-10. doi: 10.1192/bjp.bp.112.119164 [published Online First: 2013/01/11]
3. Mokdad AH, Forouzanfar MH, Daoud F, et al. Global burden of diseases, injuries, and risk factors for young people's health during 1990–2013: a systematic analysis for the Global Burden of Disease Study 2013. *The Lancet* 2016;387(10036):2383-401. doi: 10.1016/S0140-6736(16)00648-6
4. Patton GC, Sawyer SM, Santelli JS, et al. Our future: a Lancet commission on adolescent health and wellbeing. *The Lancet* 2016;387(10036):2423-78. doi: 10.1016/S0140-6736(16)00579-1
5. WHO. Health for the world's adolescents: A second chance in the second decade. Geneva: World Health Organization 2014.
6. Kessler RC, Berglund P, Demler O, et al. Lifetime prevalence and age-of-onset distributions of DSM-IV disorders in the National Comorbidity Survey Replication. *Arch Gen Psychiatry* 2005;62(6):593-602. doi: 10.1001/archpsyc.62.6.593 [published Online First: 2005/06/09]
7. IHME. Global Burden of Disease Compare: 2016 Prevalence Seattle: Institute for Health Metrics and Evaluation; 2016 [Available from: https://vizhub.healthdata.org/gbd-compare/.
8. Alisic E, Zalta AK, van Wesel F, et al. Rates of post-traumatic stress disorder in trauma-exposed children and adolescents: meta-analysis. *Br J Psychiatry* 2014;204:335-40. doi: 10.1192/bjp.bp.113.131227 [published Online First: 2014/05/03]
9. Kruk ME, Gage AD, Arsenault C, et al. High-quality health systems in the Sustainable Development Goals era: time for a revolution. *Lancet Glob Health* 2018;6(11):e1196-e252. doi: 10.1016/s2214-109x(18)30386-3 [published Online First: 2018/09/10]
10. IOM. Crossing the Quality Chasm: A New Health System for the 21st Century. Washington (DC): National Academies Press (US)
Copyright 2001 by the National Academy of Sciences. All rights reserved. 2001.
11. WHO. Quality of care: a process of making strategic choices in health systems. Geneva: World Health Organization 2006.
12. WHO. Making health services adolescent friendly: Developing national quality standards for adolescent friendly health services. Geneva: World Health Organization 2012.
13. Patel V, Saxena S. Achieving universal health coverage for mental disorders. *BMJ (Clinical research ed)* 2019;366:l4516-l16. doi: 10.1136/bmj.l4516
14. WHO. Global standards for quality health care services for adolescents. Geneva: World Health Organization, 2015.
15. Chisholm D, Flisher AJ, Lund C, et al. Scale up services for mental disorders: a call for action. *Lancet* 2007;370(9594):1241-52. doi: 10.1016/s0140-6736(07)61242-2 [published Online First: 2007/09/07]
16. Barry MM, Clarke AM, Jenkins R, et al. A systematic review of the effectiveness of mental health promotion interventions for young people in low and middle income

For peer review only - http://bmjopen.bmj.com/site/about/guidelines.xhtml

countries. *BMC Public Health* 2013;13:835. doi: 10.1186/1471-2458-13-835 [published Online First: 2013/09/13]

17. Sayal K, Amarasinghe M, Robotham S, et al. Quality standards for child and adolescent mental health in primary care. *BMC Fam Pract* 2012;13:51-51. doi: 10.1186/1471-2296-13-51
18. McDonald CC, Deatrick JA, Kassam-Adams N, et al. Community violence exposure and positive youth development in urban youth. *J Community Health* 2011;36(6):925-32. doi: 10.1007/s10900-011-9391-5
19. Slopen N, Fitzmaurice GM, Williams DR, et al. Common patterns of violence experiences and depression and anxiety among adolescents. *Soc Psychiatry Psychiatr Epidemiol* 2012;47(10):1591-605. doi: 10.1007/s00127-011-0466-5 [published Online First: 2012/01/14]
20. Finkelhor D, Ormrod RK, Turner HA. Poly-victimization: a neglected component in child victimization. *Child Abuse Negl* 2007;31(1):7-26. doi: 10.1016/j.chiabu.2006.06.008 [published Online First: 2007/01/17]
21. Cooley-Quille M, Boyd RC, Frantz E, et al. Emotional and behavioral impact of exposure to community violence in inner-city adolescents. *J Clin Child Psychol* 2001;30(2):199-206. doi: 10.1207/s15374424jccp3002_7 [published Online First: 2001/06/08]
22. Buka SL, Stichick TL, Birdthistle I, et al. Youth exposure to violence: prevalence, risks, and consequences. *Am J Orthopsychiatry* 2001;71(3):298-310. doi: 10.1037/0002-9432.71.3.298 [published Online First: 2001/08/10]
23. Hammack PL, Richards MH, Luo Z, et al. Social support factors as moderators of community violence exposure among inner-city African American young adolescents. *J Clin Child Adolesc Psychol* 2004;33(3):450-62. doi: 10.1207/s15374424jccp3303_3 [published Online First: 2004/07/24]
24. WHO. mhGAP: Mental Health Gap Action Programme: scaling up care for mental, neurological and substance use disorders Geneva: World Health Organization, 2008.
25. Nair M, Baltag V, Bose K, et al. Improving the Quality of Health Care Services for Adolescents, Globally: A Standards-Driven Approach. *The Journal of adolescent health : official publication of the Society for Adolescent Medicine* 2015;57(3):288-98. doi: 10.1016/j.jadohealth.2015.05.011
26. Moher D, Liberati A, Tetzlaff J, et al. Preferred reporting items for systematic reviews and meta-analyses: the PRISMA statement. *BMJ* 2009;339:b2535. doi: 10.1136/bmj.b2535
27. Endnote X9 [program]. London: Clarivate Analytics, 2019.
28. NIH. Quality assessment tool for observational cohort and cross-sectional studies Washington, DC: National Institutes of Health; 2014 [Available from: https://www.nhlbi.nih.gov/health-topics/study-quality-assessment-tools accessed 9 November 2019.
29. Davidson TM, Bunnell BE, Saunders BE, et al. Pilot evaluation of a tablet-based application to improve quality of care in child mental health treatment. *Behavior Therapy* 2018:No Pagination Specified.
30. Aupont O, Doerfler L, Connor DF, et al. A collaborative care model to improve access to pediatric mental health services. *Administration and policy in mental health* 2013;40(4):264-73. doi: 10.1007/s10488-012-0413-0 [published Online First: 2012/04/25]

For peer review only - http://bmjopen.bmj.com/site/about/guidelines.xhtml

31. Higa-McMillan C, Kotte A, Jackson D, et al. Overlapping and Non-overlapping Practices in Usual and Evidence-Based Care for Youth Anxiety. *The Journal of Behavioral Health Services & Research* 2017;44(4):684-94. doi: 10.1007/s11414-016-9502-2
32. Sattler AF, Ale CM, Nguyen K, et al. Use of evidence-based assessments for childhood anxiety disorders within a regional medical system. *Psychol Serv* 2016;13(4):411-18. doi: 10.1037/ser0000091 [published Online First: 2016/11/01]
33. Sattler AF, Leffler JM, Harrison NL, et al. The quality of assessments for childhood psychopathology within a regional medical center. *Psychological Services* 2019;16(4):596-604.
34. Stevens J, Klima J, Chisolm D, et al. A trial of telephone services to increase adolescent utilization of health care for psychosocial problems. *J Adolesc Health* 2009;45(6):564-70. doi: 10.1016/j.jadohealth.2009.04.003 [published Online First: 2009/11/26]
35. Mora Ringle VA, Hickey JS, Jensen-Doss A. Patterns and predictors of compliance with utilization management guidelines supporting a state policy to improve the quality of youth mental health services. *Child Youth Serv Rev* 2019;96:194-203. doi: 10.1016/j.childyouth.2018.11.035 [published Online First: 11/16]
36. Simmons MB, Elmes A, McKenzie JE, et al. Right choice, right time: Evaluation of an online decision aid for youth depression. *Health Expect* 2017;20(4):714-23. doi: 10.1111/hex.12510 [published Online First: 2016/10/18]
37. Anderson RE, Spence SH, Donovan CL, et al. Working alliance in online cognitive behavior therapy for anxiety disorders in youth: comparison with clinic delivery and its role in predicting outcome. *J Med Internet Res* 2012;14(3):e88. doi: 10.2196/jmir.1848 [published Online First: 2012/07/14]
38. Cairns AJ, Kavanagh DJ, Dark F, et al. Goal setting improves retention in youth mental health: a cross-sectional analysis. *Child & Adolescent Psychiatry & Mental Health [Electronic Resource]* 2019;13:31.
39. Ayton A, Meads G. The implementation of the care programme approach in the west midlands CAMHS: The impact of organisational diversity on patient safety - an extended audit. *International Journal of Clinical Leadership* 2013;17(4):185-200.
40. Ougrin D, Corrigall R, Poole J, et al. Comparison of effectiveness and cost-effectiveness of an intensive community supported discharge service versus treatment as usual for adolescents with psychiatric emergencies: A randomised controlled trial. *The Lancet Psychiatry* 2018;5(6):477-85.
41. Dion J, Kennedy A, Cloutier P, et al. Evaluating crisis intervention services for youth within an emergency department: A view from within. *Child Care in Practice* 2010;16(3):241-56.
42. Spenser HR, Gillies A, Maysenhoelder H. The CHAT project: Paediatricians and mental health clinicians: Working together for the sake of the children. *Journal of the Canadian Academy of Child and Adolescent Psychiatry* 2009;18(2):110-16.
43. Jager M, Reijneveld SA, Almansa J, et al. Less reduction of psychosocial problems among adolescents with unmet communication needs. *European Child & Adolescent Psychiatry* 2017;26(4):403-12. doi: 10.1007/s00787-016-0901-y
44. Jager M, Reijneveld SA, Metselaar J, et al. Discrepancies between adolescents' attributed relevance and experiences regarding communication are associated with poorer client participation and learning processes in psychosocial care. *Patient education and counseling* 2014;97(3):332-8. doi: 10.1016/j.pec.2014.08.020 [published Online First: 2014/09/17]

For peer review only - http://bmjopen.bmj.com/site/about/guidelines.xhtml

45. Kapp C, Perlini T, Jeanneret T, et al. Identifying the determinants of perceived quality in outpatient child and adolescent mental health services from the perspectives of parents and patients. *European Child & Adolescent Psychiatry* 2017;26(10):1269-77. doi: 10.1007/s00787-017-0985-z
46. Leslie HH, Hirschhorn LR, Marchant T, et al. Health systems thinking: A new generation of research to improve healthcare quality. *PLoS Med* 2018;15(10):e1002682-e82. doi: 10.1371/journal.pmed.1002682
47. Corrigan P. How stigma interferes with mental health care. *Am Psychol* 2004;59(7):614-25. doi: 10.1037/0003-066x.59.7.614 [published Online First: 2004/10/20]
48. JD L. Mental illness-related structural stigma: the downward spiral of systemic exclusion. Alberta, Canada: Mental Health Commission of Canada, 2013.
49. DuPont-Reyes MJ, Villatoro AP, Phelan JC, et al. Adolescent views of mental illness stigma: An intersectional lens. *Am J Orthopsychiatry* 2020;90(2):201-11. doi: 10.1037/ort0000425 [published Online First: 2019/08/06]
50. Mukolo A, Heflinger CA, Wallston KA. The stigma of childhood mental disorders: a conceptual framework. *J Am Acad Child Adolesc Psychiatry* 2010;49(2):92-198. doi: 10.1097/00004583-201002000-00003
51. Gulliver A, Griffiths KM, Christensen H. Perceived barriers and facilitators to mental health help-seeking in young people: a systematic review. *BMC Psychiatry* 2010;10(1):113. doi: 10.1186/1471-244X-10-113
52. Radez J, Reardon T, Creswell C, et al. Why do children and adolescents (not) seek and access professional help for their mental health problems? A systematic review of quantitative and qualitative studies. *European Child & Adolescent Psychiatry* 2020 doi: 10.1007/s00787-019-01469-4
53. Commission CQ. Review of children and young people's mental health services: Phase One supporting documentation: Summary of recent policy and literature. London: Care Quality Commission, 2017.
54. O'Connor AM, Légaré F, Stacey D. Risk communication in practice: the contribution of decision aids. *Bmj* 2003;327(7417):736-40. doi: 10.1136/bmj.327.7417.736 [published Online First: 2003/09/27]
55. Hawley KM, Garland AF. Working Alliance in Adolescent Outpatient Therapy: Youth, Parent and Therapist Reports and Associations with Therapy Outcomes. *Child & Youth Care Forum* 2008;37(2):59-74. doi: 10.1007/s10566-008-9050-x
56. Marker CD, Comer JS, Abramova V, et al. The reciprocal relationship between alliance and symptom improvement across the treatment of childhood anxiety. *J Clin Child Adolesc Psychol* 2013;42(1):22-33. doi: 10.1080/15374416.2012.723261 [published Online First: 2012/09/27]
57. Anstiss D, Davies A. 'Reach Out, Rise Up': The efficacy of text messaging in an intervention package for anxiety and depression severity in young people. *Child Youth Serv Rev* 2015;58:99-103. doi: https://doi.org/10.1016/j.childyouth.2015.09.011
58. Gondek D, Edbrooke-Childs J, Velikonja T, et al. Facilitators and Barriers to Person-centred Care in Child and Young People Mental Health Services: A Systematic Review. *Clin Psychol Psychother* 2017;24(4):870-86. doi: 10.1002/cpp.2052 [published Online First: 2016/12/03]

For peer review only

59. Weithorn LA, Campbell SB. The Competency of Children and Adolescents to Make Informed Treatment Decisions. *Child Development* 1982;53(6):1589-98. doi: 10.2307/1130087
60. Langer DA, Jensen-Doss A. Shared Decision-Making in Youth Mental Health Care: Using the Evidence to Plan Treatments Collaboratively. *J Clin Child Adolesc Psychol* 2018;47(5):821-31. doi: 10.1080/15374416.2016.1247358 [published Online First: 2016/12/03]
61. Psychiatry FoCaAPaFoGA. Good mental health services for young people. London: Royal College of Psychiatrists, 2017.
62. C L. Working at the CAMHS/Adult Interface: Good practice guidance for the provision of psychiatric services to adolescents/young adults. London: Royal College of Psychiatrists 2008.
63. Birchwood M, Singh SP. Mental health services for young people: matching the service to the need. *Br J Psychiatry Suppl* 2013;54:s1-2. doi: 10.1192/bjp.bp.112.119149 [published Online First: 2013/01/11]
64. M F. Supporting young people's mental health: eight points for action: a policy briefing from the mental health foundation. London: Mental Health Foundation 2007.
65. Smith K, Leon L, Foundation MH, et al. Turned Upside Down: Developing Community-Based Crisis Services for 16-25-Year-Olds Experiencing a Mental Health Crisis: Mental Health Foundation 2001.
66. O'Brien D, Harvey K, Howse J, et al. Barriers to managing child and adolescent mental health problems: a systematic review of primary care practitioners' perceptions. *Br J Gen Pract* 2016;66(651):e693-707. doi: 10.3399/bjgp16X687061 [published Online First: 2016/09/14]
67. Kutcher S, Davidson S, Manion I. Child and youth mental health: Integrated health care using contemporary competency-based teams. *Paediatr Child Health* 2009;14(5):315-18. doi: 10.1093/pch/14.5.315
68. Kilbourne AM, Beck K, Spaeth-Rublee B, et al. Measuring and improving the quality of mental health care: a global perspective. *World Psychiatry* 2018;17(1):30-38. doi: 10.1002/wps.20482
69. Vallance AK. 'Shhh! Please don't tell…' Confidentiality in child and adolescent mental health. *BJPsych Advances* 2018;22(1):25-35. doi: 10.1192/apt.bp.114.013854 [published Online First: 01/02]
70. OECD. Making mental health count. Focus on health. . Paris: OECD, 2014.
71. WHO. Integrating mental health into primary care: A global perspective. Geneva: World Health Organization, 2008.
72. Patel V, Saxena S, Lund C, et al. The Lancet Commission on global mental health and sustainable development. *The Lancet* 2018;392(10157):1553-98. doi: 10.1016/S0140-6736(18)31612-X
73. Knaak S, Patten S, Ungar T. Mental illness stigma as a quality-of-care problem. *Lancet Psychiatry* 2015;2(10):863-4. doi: 10.1016/s2215-0366(15)00382-x [published Online First: 2015/10/16]
74. Sawyer SM, Azzopardi PS, Wickremarathne D, et al. The age of adolescence. *The Lancet Child & Adolescent Health* 2018;2(3):223-28. doi: https://doi.org/10.1016/S2352-4642(18)30022-1
75. WHA. Comprehensive mental health action plan 2013-2020 WHA66.8, 13.3: World Health Assembly 2013.

For peer review only

For peer review only - http://bmjopen.bmj.com/site/about/guidelines.xhtml

Table 1 References
Knaak S, Patten S, Ungar T. Mental illness stigma as a quality-of-care problem. *Lancet Psychiatry* 2015;2(10):863-4.

Saraceno B, van Ommeren M, Batniji R, et al. Barriers to improvement of mental health services in low-income and middle-income countries. *Lancet* 2007;370(9593):1164-74.
McPherson A. Adolescents in primary care. *Bmj* 2005;330(7489):465-7.

Svirydzenka N, Ronzoni P, Dogra N. Meaning and barriers to quality care service provision in Child and Adolescent Mental Health Services: Qualitative study of stakeholder perspectives. *BMC Health Serv Res* 2017;17(1):151.

Box 1 References
Kruk ME, Gage AD, Arsenault C, et al. High-quality health systems in the Sustainable Development Goals ear: time for a revolution. *Lancet Glob Health* 2018;6(11):e1196-e252.

WHO, OECD, WB. Delivering Quality Health Services: A Global Imperative for Universal Health Coverage. Geneva: World Health Organization 2018.

IOM. Crossing the Quality Chasm: A New Health System for the 21st Century. Washington(DC): National Academies Press(US).

Table 3 References
Davidson TM, Bunnell BE, Saunders BE, et al. Pilot evaluation of a tablet-based application to improve quality of care in child mental health treatment. Behavior Therapy 2018:No Pagination Specified.

Jager M, Reijneveld SA, Almansa J, et al. Less reduction of psychosocial problems among adolescents with unmet communication needs. European Child & Adolescent Psychiatry 2017;26(4):403-12. doi: 10.1007/s00787-016-0901-y

Ougrin D, Corrigall R, Poole J, et al. Comparison of effectiveness and cost-effectiveness of an intensive community supported discharge service versus treatment as usual for adolescents with psychiatric emergencies: A randomised controlled trial. The Lancet Psychiatry 2018;5(6):477-85.

Dion J, Kennedy A, Cloutier P, et al. Evaluating crisis intervention services for youth within an emergency department: A view from within. Child Care in Practice 2010;16(3):241-56.

Spenser HR, Gillies A, Maysenhoelder H. The CHAT project: Paediatricians and mental health clinicians: Working together for the sake of the children. Journal of the Canadian Academy of Child and Adolescent Psychiatry 2009;18(2):110-16.
Ayton A, Meads G. The implementation of the care programme approach in the west midlands CAMHS: The impact of organisational diversity on patient safety - an extended audit. International Journal of Clinical Leadership 2013;17(4):185-200.

Aupont O, Doerfler L, Connor DF, et al. A collaborative care model to improve access to pediatric mental health services. Administration and policy in mental health 2013;40(4):264-73. doi: 10.1007/s10488-012-0413-0 [published Online First: 2012/04/25]

For peer review only

Simmons MB, Elmes A, McKenzie JE, et al. Right choice, right time: Evaluation of an online decision aid for youth depression. Health Expect 2017;20(4):714-23. doi: 10.1111/hex.12510 [published Online First: 2016/10/18]

Jager M, Reijneveld SA, Metselaar J, et al. Discrepancies between adolescents' attributed relevance and experiences regarding communication are associated with poorer client participation and learning processes in psychosocial care. Patient education and counseling 2014;97(3):332-8. doi: 10.1016/j.pec.2014.08.020 [published Online First: 2014/09/17]

Stevens J, Klima J, Chisolm D, et al. A trial of telephone services to increase adolescent utilization of health care for psychosocial problems. J Adolesc Health 2009;45(6):564-70. doi: 10.1016/j.jadohealth.2009.04.003 [published Online First: 2009/11/26]

Anderson RE, Spence SH, Donovan CL, et al. Working alliance in online cognitive behavior therapy for anxiety disorders in youth: comparison with clinic delivery and its role in predicting outcome. J Med Internet Res 2012;14(3):e88. doi: 10.2196/jmir.1848 [published Online First: 2012/07/14]

Kapp C, Perlini T, Jeanneret T, et al. Identifying the determinants of perceived quality in outpatient child and adolescent mental health services from the perspectives of parents and patients. European Child & Adolescent Psychiatry 2017;26(10):1269-77. doi: 10.1007/s00787-017-0985-z

Cairns AJ, Kavanagh DJ, Dark F, et al. Goal setting improves retention in youth mental health: a cross-sectional analysis. Child & Adolescent Psychiatry & Mental Health [Electronic Resource] 2019;13:31.

Mora Ringle VA, Hickey JS, Jensen-Doss A. Patterns and predictors of compliance with utilization management guidelines supporting a state policy to improve the quality of youth mental health services. Child Youth Serv Rev 2019;96:194-203. doi: 10.1016/j.childyouth.2018.11.035 [published Online First: 11/16]

Sattler AF, Leffler JM, Harrison NL, et al. The quality of assessments for childhood psychopathology within a regional medical center. Psychological Services 2019;16(4):596-604.

Sattler AF, Ale CM, Nguyen K, et al. Use of evidence-based assessments for childhood anxiety disorders within a regional medical system. Psychol Serv 2016;13(4):411-18. doi: 10.1037/ser0000091 [published Online First: 2016/11/01]

Higa-McMillan C, Kotte A, Jackson D, et al. Overlapping and Non-overlapping Practices in Usual and Evidence-Based Care for Youth Anxiety. The Journal of Behavioral Health Services & Research 2017;44(4):684-94. doi: 10.1007/s11414-016-9502-2.

Table 4 References

Sawyer SM, Ambresin AE, Bennett KE, et al. A measurement framework for quality health care for adolescents in hospital. J Adolesc Health 2014;55(4):484-90. doi: 10.1016/j.jadohealth.2014.01.023 [published Online First: 2014/04/05].

Ambresin A-E, Bennett K, Patton GC, et al. Assessment of youth-friendly health care: a systematic review of indicators drawn from young people's perspectives. The Journal of adolescent health : official publication of the Society for Adolescent Medicine 2013;52(6):670-81. doi: 10.1016/j.jadohealth.2012.12.014.

For peer review only - http://bmjopen.bmj.com/site/about/guidelines.xhtml

NHS. You're Welcome Quality Criteria: Making health services young people friendly. London: NHS, 2007.

Sayal K, Amarasinghe M, Robotham S, et al. Quality standards for child and adolescent mental health in primary care. *BMC Fam Pract* 2012;13:51-51. doi: 10.1186/1471-2296-13-51

For peer review only

Figure 1. Flow diagram of studies

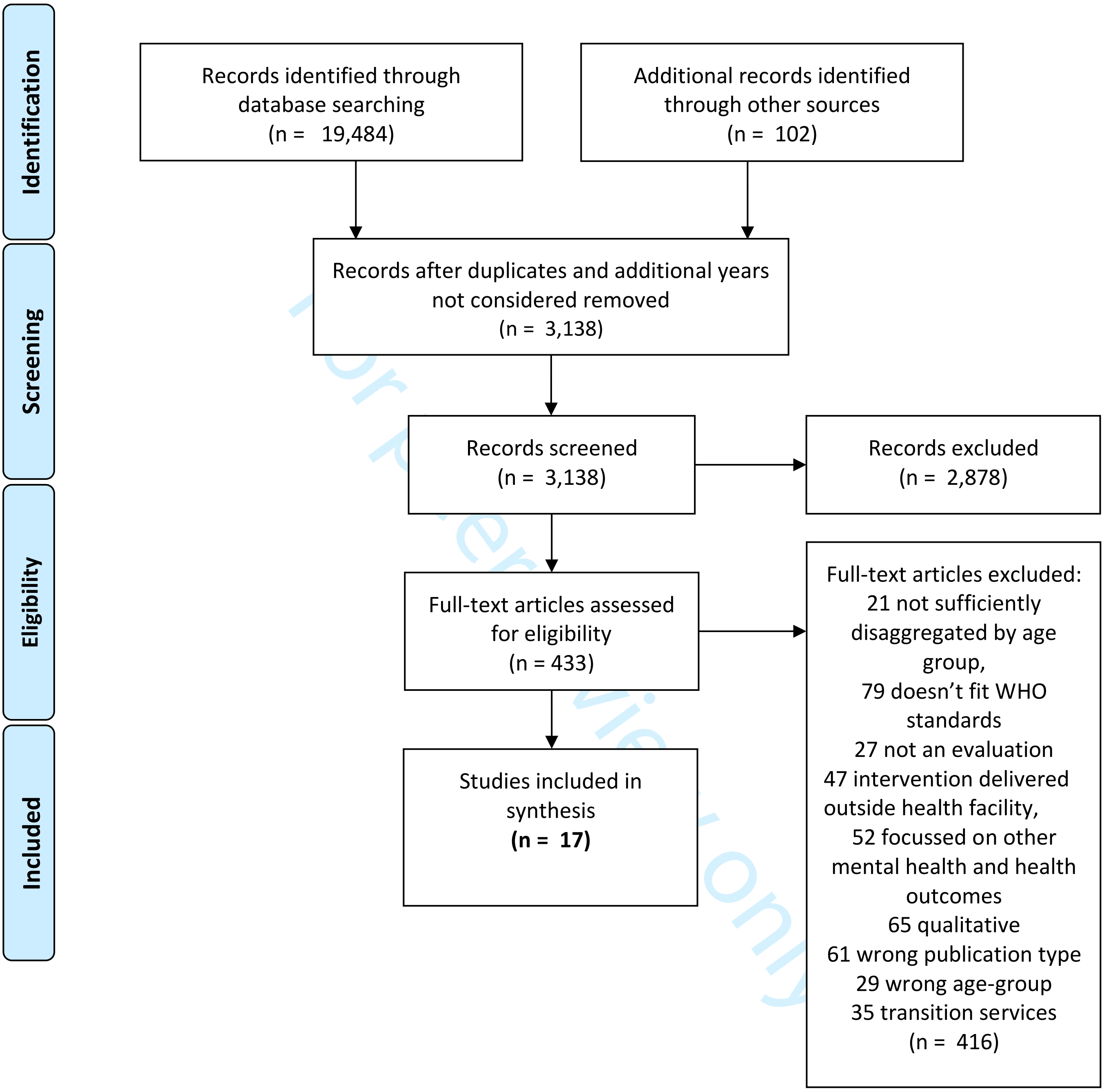

For peer review only - http://bmjopen.bmj.com/site/about/guidelines.xhtml

For peer review only

For peer review only - http://bmjopen.bmj.com/site/about/guidelines.xhtml

Supplemental Material 1: Process and Output Criteria used for WHO Quality Standards to Select Studies[1]

For *adolescent mental health literacy*, this involved identifying and assessing studies using the following criteria:

	**Adolescent mental health literacy: Selection criteria for studies**
**PROCESS**	the provision of mental health education and counselling to adolescents by healthcare providers and services that are age and developmentally appropriate
	the provision of information about the availability of mental health, social, and other services to adolescents by healthcare providers and services
	outreach activities by the healthcare facility to promote mental health and increase the use of adolescent mental health services
**OUTPUT**	adolescents increased knowledge about mental health and awareness about mental health services

For *appropriate package of services*, we identified and assessed studies according to the following:

	**Appropriate package of services: Selection criteria for studies**
**PROCESS**	within healthcare services, the provision of a package of mental health information, counselling, diagnostic, treatment and care services to adolescents that meets their needs
	referral to appropriate services
**OUTPUT**	adolescents receive a package of services that meets their needs, within healthcare or referral services

For *provider competencies*, this involved identifying and assessing studies using the following criteria:

	**Provider competencies: Selection criteria for studies**
**PROCESS**	healthcare providers adhere to and implement adolescent mental health evidence-based guidelines and protocols
	healthcare providers provide friendly services to adolescents and respect their rights to information, privacy, confidentiality, non-discrimination, non-judgement attitude, and respectful care
**OUTPUT**	adolescents receive effective mental health services
	adolescents obtain mental health services that are friendly, supportive, respectful, non-discriminatory and non-judgemental manner, and know their rights in healthcare
	adolescents receive from the healthcare facility accurate, age-appropriate and clear information to facilitate informed choice

References

1. WHO. Global standards for quality health care services for adolescents. Geneva: World Health Organization, 2015.

For peer review only

PROSPERO
International prospective register of systematic reviews

Evaluating quality in adolescent mental health service delivery: a systematic review

Meaghen Quinlan-Davidson, Kathryn Roberts, Delan Devakumar, Susan Sawyer, Ligia Kiss

Citation

Meaghen Quinlan-Davidson, Kathryn Roberts, Delan Devakumar, Susan Sawyer, Ligia Kiss. Evaluating quality in adolescent mental health service delivery: a systematic review. PROSPERO 2020 CRD42020161318 Available from: https://www.crd.york.ac.uk/prospero/display_record.php?ID=CRD42020161318

Review question

What is the global evidence on the aspects of quality of adolescent mental health services (based on the WHO Global standards for quality healthcare services for adolescents) including adolescent mental health literacy, provider competencies, and appropriate packages of services for adolescents with depressive, anxiety, and post-traumatic stress disorders?

Searches

-2008-2018

-Sources include (but are not limited to) PubMed, PscyINFO, MEDLINE, EMBASE, LILACS

-Reference checking of articles generated from electronic database

-Hand searching of references of identified manuscripts

-No restriction on language

Types of study to be included

Evaluations or assessments of RCTs, case-control, cohort, cross-sectional, longitudinal, mixed-methods (quantitative data only).

Condition or domain being studied

Quality in adolescent mental health services.

Participants/population

Study participants include individual patients (10-19 years of age) using mental healthcare services, or adolescents in the community asked where they would seek services for depression, anxiety, or PTSD for themselves; and providers of mental health services, including general practitioners, psychologists, and psychiatrists.

Where study participants include children and adolescents together or adolescents and youth together, the study will be included when the results are sufficiently disaggregated that they can be interpreted for at least part of the 10-19 year old age span.

Intervention(s), exposure(s)

Studies that evaluate or assess aspects of quality measures (as conforms to the WHO Global standards for quality healthcare services for adolescents) applied to existing adolescent mental health services, who have used, are currently using, or could potentially seek to use mental health services, or are exposed to interventions or strategies within established mental health services, regardless of health facility level.

Comparator(s)/control

For intervention studies only: Intervention vs. control arm (randomized trial), quasi-experimental (open trial with pre- and post-intervention design or matched control group).

Main outcome(s)

For peer review only

NIHR | National Institute for Health Research

PROSPERO
International prospective register of systematic reviews

Studies that include quality, as conforms to the WHO WHO Global standards for quality healthcare services for adolescents, including:

-Adolescents' health literacy (The health facility implements systems to ensure that adolescents are knowledgeable about their own health, and they know where and when to obtain health services);

-Appropriate package of services (The health facility provides a package of information, counselling, diagnostic, treatment and care services that fulfils the needs of all adolescents. Services are provided in the facility and through referral linkages and outreach);

-Providers' competencies (Health care providers demonstrate the technical competence required to provide effective health services to adolescents. Both health-care providers and support staff respect, protect and fulfil adolescents' rights to information, privacy, confidentiality, non-discrimination, non-judgmental attitude and respect)

* Measures of effect

Not applicable.

Additional outcome(s)

None.

* Measures of effect

Not applicable.

Data extraction (selection and coding)

All results from the above mentioned search strategy will be exported to Endnote, an electronic reference manager; duplicates will be removed. Independently, two authors (MQD and KR) will screen the titles and abstracts of all studies identified through the search strategy that potentially meet the inclusion criteria mentioned above. The full text of these potentially eligible studies will be retrieved and independently assessed for eligibility by the two team members. The main data to be extracted will include study design, publication type, area of quality, reason (if any) for exclusion, type of service and mental health issue, purpose of study, conceptualisation of quality or quality in relation to or effect of quality on, intervention/measured used, sample, outcomes, and location. Disagreement over the eligibility of particular studies will be resolved through discussion. Discussion will be held to resolve any discrepancies, with additional authors when needed.

Risk of bias (quality) assessment

NIH Quality Assessment Tool

Strategy for data synthesis

Random effects meta-analyses will be conducted to determine intervention effects, if sufficient data exist and levels of heterogeneity are appropriate. A minimum of three studies will be required per meta-analysis. If data cannot be pooled between intervention studies, findings will be reported in narrative synthesis. Results will be organised and interpreted according to the WHO Global Standards for Quality Healthcare Services for Adolescents. This will include the number of studies that address each element of quality, as well as a summary of the results of each study. Also, results on the conceptualisation of quality or how quality was mentioned in the article will be summarised, geographic location of the studies, and a search and selection strategy will be presented. A table, summarising the characteristics of the study, will be included that provides information on the service, intervention description, study/evaluation design, target population, element of quality addressed, results, and quality assessment will be included.

Analysis of subgroups or subsets

Not applicable.

Contact details for further information

Meaghen Quinlan-Davidson
meaghen.quinlan-davidson.17@ucl.ac.uk

For peer review only

PROSPERO
International prospective register of systematic reviews

Organisational affiliation of the review

Institute for Global Health, University College London

Review team members and their organisational affiliations

Miss Meaghen Quinlan-Davidson. Institute for Global Health, University College London
Miss Kathryn Roberts. Institute for Global Health, University College London
Dr Delan Devakumar. Institute for Global Health, University College London
Dr Susan Sawyer. Centre for Adolescent Health, Royal Children's Hospital, Melbourne, Australia
Dr Ligia Kiss. Institute for Global Health, University College London

Type and method of review

Service delivery, Systematic review

Anticipated or actual start date

12 November 2019

Anticipated completion date

31 January 2020

Funding sources/sponsors

None.

Conflicts of interest

Language

English

Country

England

Stage of review

Review Ongoing

Subject index terms status

Subject indexing assigned by CRD

Subject index terms

Adolescent; Adolescent Health Services; Delivery of Health Care; Humans; Mental Health Services

Date of registration in PROSPERO

27 January 2020

Date of first submission

05 December 2019

Details of any existing review of the same topic by the same authors

Stage of review at time of this submission

For peer review only

Stage	Started	Completed
Preliminary searches	Yes	No
Piloting of the study selection process	Yes	No
Formal screening of search results against eligibility criteria	Yes	No
Data extraction	No	No
Risk of bias (quality) assessment	No	No
Data analysis	No	No

The record owner confirms that the information they have supplied for this submission is accurate and complete and they understand that deliberate provision of inaccurate information or omission of data may be construed as scientific misconduct.

The record owner confirms that they will update the status of the review when it is completed and will add publication details in due course.

Versions

27 January 2020

PROSPERO
This information has been provided by the named contact for this review. CRD has accepted this information in good faith and registered the review in PROSPERO. The registrant confirms that the information supplied for this submission is accurate and complete. CRD bears no responsibility or liability for the content of this registration record, any associated files or external websites.

For peer review only

PRISMA 2009 Checklist

Section/topic	#	Checklist item	Reported on page #
**TITLE**			
Title	1	Identify the report as a systematic review, meta-analysis, or both.	1
**ABSTRACT**			
Structured summary	2	Provide a structured summary including, as applicable: background; objectives; data sources; study eligibility criteria, participants, and interventions; study appraisal and synthesis methods; results; limitations; conclusions and implications of key findings; systematic review registration number.	2
**INTRODUCTION**			
Rationale	3	Describe the rationale for the review in the context of what is already known.	4-7
Objectives	4	Provide an explicit statement of questions being addressed with reference to participants, interventions, comparisons, outcomes, and study design (PICOS).	7
**METHODS**			
Protocol and registration	5	Indicate if a review protocol exists, if and where it can be accessed (e.g., Web address), and, if available, provide registration information including registration number.	7
Eligibility criteria	6	Specify study characteristics (e.g., PICOS, length of follow-up) and report characteristics (e.g., years considered, language, publication status) used as criteria for eligibility, giving rationale.	7-8
Information sources	7	Describe all information sources (e.g., databases with dates of coverage, contact with study authors to identify additional studies) in the search and date last searched.	8
Search	8	Present full electronic search strategy for at least one database, including any limits used, such that it could be repeated.	9
Study selection	9	State the process for selecting studies (i.e., screening, eligibility, included in systematic review, and, if applicable, included in the meta-analysis).	7-9
Data collection process	10	Describe method of data extraction from reports (e.g., piloted forms, independently, in duplicate) and any processes for obtaining and confirming data from investigators.	9
Data items	11	List and define all variables for which data were sought (e.g., PICOS, funding sources) and any assumptions and simplifications made.	7-9
Risk of bias in individual studies	12	Describe methods used for assessing risk of bias of individual studies (including specification of whether this was done at the study or outcome level), and how this information is to be used in any data synthesis.	9
Summary measures	13	State the principal summary measures (e.g., risk ratio, difference in means).	9
Synthesis of results	14	Describe the methods of handling data and combining results of studies, if done, including measures of consistency (e.g., I^2) for each meta-analysis.	9

For peer review only - http://bmjopen.bmj.com/site/about/guidelines.xhtml

PRISMA 2009 Checklist

Section/topic	#	Checklist item	Reported on page #
Risk of bias across studies	15	Specify any assessment of risk of bias that may affect the cumulative evidence (e.g., publication bias, selective reporting within studies).	9
Additional analyses	16	Describe methods of additional analyses (e.g., sensitivity or subgroup analyses, meta-regression), if done, indicating which were pre-specified.	NA
**RESULTS**			
Study selection	17	Give numbers of studies screened, assessed for eligibility, and included in the review, with reasons for exclusions at each stage, ideally with a flow diagram.	9-10
Study characteristics	18	For each study, present characteristics for which data were extracted (e.g., study size, PICOS, follow-up period) and provide the citations.	11-24
Risk of bias within studies	19	Present data on risk of bias of each study and, if available, any outcome level assessment (see item 12).	11-24
Results of individual studies	20	For all outcomes considered (benefits or harms), present, for each study: (a) simple summary data for each intervention group (b) effect estimates and confidence intervals, ideally with a forest plot.	11-28
Synthesis of results	21	Present results of each meta-analysis done, including confidence intervals and measures of consistency.	NA
Risk of bias across studies	22	Present results of any assessment of risk of bias across studies (see Item 15).	25
Additional analysis	23	Give results of additional analyses, if done (e.g., sensitivity or subgroup analyses, meta-regression [see Item 16]).	NA
**DISCUSSION**			
Summary of evidence	24	Summarize the main findings including the strength of evidence for each main outcome; consider their relevance to key groups (e.g., healthcare providers, users, and policy makers).	29-35
Limitations	25	Discuss limitations at study and outcome level (e.g., risk of bias), and at review-level (e.g., incomplete retrieval of identified research, reporting bias).	35
Conclusions	26	Provide a general interpretation of the results in the context of other evidence, and implications for future research.	35
**FUNDING**			
Funding	27	Describe sources of funding for the systematic review and other support (e.g., supply of data); role of funders for the systematic review.	36

From: Moher D, Liberati A, Tetzlaff J, Altman DG, The PRISMA Group (2009). Preferred Reporting Items for Systematic Reviews and Meta-Analyses: The PRISMA Statement. PLoS Med 6(7): e1000097. doi:10.1371/journal.pmed1000097

For more information, visit: www.prisma-statement.org.

For peer review only - http://bmjopen.bmj.com/site/about/guidelines.xhtml

BMJ Open

Evaluating Quality in Adolescent Mental Health Services: A Systematic Review

Journal:	*BMJ Open*
Manuscript ID	bmjopen-2020-044929.R1
Article Type:	Original research
Date Submitted by the Author:	22-Mar-2021
Complete List of Authors:	Quinlan-Davidson, Meaghen; University College London Institute for Global Health,<br>Roberts, Kathryn; University College London Institute for Global Health<br>Devakumar, Delan; University College London Medical School,<br>Sawyer, Susan; The Royal Children's Hospital Melbourne, Centre for Adolescent Health<br>Cortez, Rafael; World Bank Group<br>Kiss, Lígia; University College London, Institute for Global Health
**Primary Subject Heading**:	Mental health
Secondary Subject Heading:	Health services research, Global health
Keywords:	Quality in health care < HEALTH SERVICES ADMINISTRATION & MANAGEMENT, Child & adolescent psychiatry < PSYCHIATRY, MENTAL HEALTH, International health services < HEALTH SERVICES ADMINISTRATION & MANAGEMENT

SCHOLARONE™
Manuscripts

BMJ

I, the Submitting Author has the right to grant and does grant on behalf of all authors of the Work (as defined in the below author licence), an exclusive licence and/or a non-exclusive licence for contributions from authors who are: i) UK Crown employees; ii) where BMJ has agreed a CC-BY licence shall apply, and/or iii) in accordance with the terms applicable for US Federal Government officers or employees acting as part of their official duties; on a worldwide, perpetual, irrevocable, royalty-free basis to BMJ Publishing Group Ltd ("BMJ") its licensees and where the relevant Journal is co-owned by BMJ to the co-owners of the Journal, to publish the Work in this journal and any other BMJ products and to exploit all rights, as set out in our licence.

The Submitting Author accepts and understands that any supply made under these terms is made by BMJ to the Submitting Author unless you are acting as an employee on behalf of your employer or a postgraduate student of an affiliated institution which is paying any applicable article publishing charge ("APC") for Open Access articles. Where the Submitting Author wishes to make the Work available on an Open Access basis (and intends to pay the relevant APC), the terms of reuse of such Open Access shall be governed by a Creative Commons licence – details of these licences and which Creative Commons licence will apply to this Work are set out in our licence referred to above.

Other than as permitted in any relevant BMJ Author's Self Archiving Policies, I confirm this Work has not been accepted for publication elsewhere, is not being considered for publication elsewhere and does not duplicate material already published. I confirm all authors consent to publication of this Work and authorise the granting of this licence.

For peer review only

Title: Evaluating Quality in Adolescent Mental Health Services: A Systematic Review

Meaghen Quinlan-Davidson[1], Kathryn J. Roberts[1], Delan Devakumar[1], Susan M. Sawyer[2], Rafael Cortez[3], Ligia Kiss[1]

[1] Institute for Global Health, University College London, 30 Guilford St, London WC1N 1DP, United Kingdom;
[2] Centre for Adolescent Health, Royal Children's Hospital; Murdoch Children's Research Institute; and Department of Paediatrics, The University of Melbourne, 50 Flemington Road, Parkville, Victoria 3052 Australia;
[3] The World Bank, 1818 H Street, NW Washington DC 20433 USA

Correspondence to:
Meaghen Quinlan-Davidson
Institute for Global Health
University College London
London
WC1N 1DP
United Kingdom
Email: meaghen.quinlan-davidson.17@ucl.ac.uk
Phone: +44 (0)20 7679 2000

Word count: 6,058 words

For peer review only

ABSTRACT

Objectives. To evaluate the quality of adolescent mental health service provision globally, according to the WHO Global Standards of adolescent mental health literacy, appropriate package of services, and provider competencies.

Design and data sources. Systematic review of 5 databases, and screening of eligible articles, from 1 January 2008 to 31 December 2020.

Study eligibility criteria. We focussed on quantitative and mixed-method studies that evaluated adolescent mental health literacy, appropriate package of services, and provider competencies in mental health services, and that targeted depression, anxiety, and post-traumatic stress disorder among adolescents (10-19 years). This included adolescents exposed to interventions or strategies within mental health services.

Study appraisal and synthesis methods. Study quality was assessed using the National Institutes for Health Study Quality Assessment Tools. Data was extracted and grouped based on WHO quality Standards.

Results. Of the 20,104 studies identified, 20 articles were included. The majority of studies came from high-income countries, with one from a low-income country. Most of the studies did not conceptualise quality. Results found that an online decision aid was evaluated to increase adolescent mental health literacy. Studies that targeted an appropriate package of services evaluated the quality of engagement between the therapist and adolescent, patient-centred communication, mental health service use, linkages to mental health services, health facility culture, and intensive community treatment. Provider competencies focussed on studies that evaluated confidence in managing and referring adolescents, collaboration between health facility levels, evidence-based practices, and technology use.

Conclusions and Implications. There is limited evidence on quality measures in adolescent mental health services (as conforms to the WHO Global Standards), pointing to a global evidence gap for adolescent mental health services. There are several challenges to overcome, including a need to develop consensus on quality and methods to measure quality in mental health settings.

PROSPERO registration number. CRD42020161318

For peer review only - http://bmjopen.bmj.com/site/about/guidelines.xhtml

Strength and limitations of this study

- This is the first review to investigate quality measures for adolescent mental health services globally.
- This review highlights a critical gap in evidence on quality in adolescent mental health services.
- This review was limited to mental health services to adolescents in health facilities. It did not review the quality of mental health preventative or health promotion activities that are typically provided in other settings such as communities or schools.
- The review investigated adolescents with depression, anxiety, and PTSD and not those with other mental health conditions.

For peer review only

For peer review only - http://bmjopen.bmj.com/site/about/guidelines.xhtml

INTRODUCTION

Globally, a significant burden of disease is due to mental health disorders.[1-3] Symptoms largely emerge during childhood, adolescence, and young adulthood,[1-4] with estimates suggesting that by 24 years of age, 75% of adult mental health disorders have appeared.[5, 6] Current global estimates indicate that 14.8% of young people aged 10-24 years have a mental health disorder,[7] with the most common being unipolar depressive disorders, anxiety disorders, and self-harm.[5] Depressive disorders rank as the fourth highest contributor to disability adjusted life years (DALYs) in 10-14 year olds (4.3%) and the third highest among 15-19 year olds (5.6%). Meanwhile, depressive and anxiety disorders rank among the top six contributors to years lived with disability (YLDs) for 10-14 year olds (7.4% and 5.0%, respectively) and among the top five for 15-19 year olds (11.2% and 5.8%, respectively).[3] In addition, a meta-analysis found that 16% of children and adolescents (2-18 years of age) develop post-traumatic stress disorder (PTSD) after exposure to a traumatic event.[8] Depression, anxiety and PTSD can be influenced by internal and external stressors.[9-11] These stressors can arise from living in challenging settings, experiencing traumatic events and life transitions,[10, 12, 13] and are exacerbated by the COVID-19 pandemic.[14, 15] Despite their prevalence, the quality of mental health services, particularly within challenging environments, is limited.[9]

Mental health conditions have short- and long-term health, education, social and economic impacts including early school leaving, social isolation, substance misuse, chronic unemployment, and high economic costs for families, communities and health systems.[1-4] Yet, despite increased attention on adolescent mental health in recent years, adolescents with mental health conditions experience worse quality of care in comparison to other age groups.[16] Poor quality healthcare is associated with worse health outcomes, comorbid conditions, and a lack of trust in the health system. It also places the adolescent at an increased risk of death due to inadequate healthcare management, treatment and follow-up.[16]

Quality in mental healthcare is an important component of service delivery that contributes to adolescents seeking, receiving, and continuing care. The Institute of Medicine (IOM) and World Health Organization (WHO) define quality healthcare as “the degree to which health services for individuals and populations increase the likelihood of desired health outcomes and are consistent with current professional knowledge”.[17] Adolescents benefit from mental health services that are adolescent-friendly, defined as accessible, acceptable, equitable, appropriate, and effective.[18, 19]

For peer review only - http://bmjopen.bmj.com/site/about/guidelines.xhtml

There are many multifaceted barriers that influence quality in mental healthcare services (table 1).

Table 1. Health Service Barriers to Quality Adolescent Mental Health Services

**Health Service Barriers**
• Limited financial resources<br>• Limited literacy on quality in mental healthcare, with limited or lack of quality criteria or measures<br>• Lack of routine assessment of quality in services<br>• Limited availability of specialised mental health care providers or those appropriately trained in mental health to engage and respond to adolescents' mental health needs<br>• Healthcare provider stigma, as a behavioural barrier to quality mental healthcare<br>• Lack of information, education, and engagement from the health system to the community about adolescent mental health services and promotion of adolescent mental health literacy<br>• Limited adolescent involvement in decisions about their treatment and care plans that are required for patient-centred care<br>• Lack of youth-friendly, or patient-centred, services that ensure privacy, confidentiality (including confidentiality from parents and other service providers), respect, high-quality communication, and services that are non-judgemental and free of embarrassment<br>• Lack of equitable (geographically and socioeconomically) distribution of mental health services<br>• Overburdened primary care systems which make it hard to integrate adolescent mental health services<br>• Lack of mental health leadership from policy-and decision-makers at local and national government levels to champion quality in adolescent mental health services

Sources: Knaak S, Patten S, Ungar T. Mental illness stigma as a quality-of-care problem. *Lancet Psychiatry* 2015;2(10):863-4; Saraceno B, van Ommeren M, Batniji R, et al. Barriers to improvement of mental health services in low-income and middle-income countries. *Lancet* 2007;370(9593):1164-74; McPherson A. Adolescents in primary care. *BMJ* 2005;330(7489):465-7; Svirydzenka N, Ronzoni P, Dogra N. Meaning and barriers to quality care service provision in Child and Adolescent Mental Health Services: Qualitative study of stakeholder perspectives. *BMC Health Serv Res* 2017;17(1):151.

For adolescents and their families, barriers to quality mental healthcare start with access to these services. The stigma and discrimination associated with mental health disorders, as well as how adolescents, families, communities, and health services conceptualise emotional suffering and distress are significant barriers to access and use of quality mental health services [20]. Even in well-resourced contexts where mental health services are available, social and cultural conceptualisations of mental health influence the uptake and subsequent coverage of adolescent mental health services [20]. Adolescents are often less-experienced users of mental health services, with inadequate mental health literacy, including literacy about quality of care. They value privacy, confidentiality, and patient-centred care, which includes respect, high-quality communication, and a therapist who is

For peer review only - http://bmjopen.bmj.com/site/about/guidelines.xhtml

responsive to their needs. They seek to avoid judgement and embarrassment, and fear that their parents will be informed can limit their uptake of health services [4].

There have been several global initiatives focussing on quality of healthcare in recent years (box 1). In 2015, the WHO developed *Global standards for quality health care services for adolescents* to support health service delivery and quality improvements for adolescents in primary and referral level facilities.[21] The aim of these standards was to increase adolescents' use of health services, improve health outcomes, ensure a minimal level of quality, and fulfil their rights to health care.[21] There are eight standards, all of which are important to ensuring quality in healthcare services. This includes adolescents' health literacy, community support, appropriate package of services, provider competencies, facility characteristics, equity and non-discrimination, data and quality improvement, and adolescent participation[21]. Notwithstanding the relevance of all standards to quality health services, three standards are particularly important for help-seeking behaviour among adolescents.[22-24]

1. *Adolescents' health literacy* whereby adolescents know about mental health and their own mental health (through the health facility), as well as knowing where health services are located and when to go.[21]
2. An appropriate package of services in which the health facility meets the needs of the adolescent by providing evidence-based information, counselling, correct diagnoses, treatment, and care services.[21]
3. Providers' competencies, whereby healthcare providers are competent and provide effective care to adolescents (including respecting, protecting, and fulfilling the rights of adolescents).[21]

These standards reflect the possibilities of interactions between adolescents and health services in terms of access, communication and competency of care.[16] Yet to date, there has been little research evaluating these standards with no systematic review of the evidence. Recent literature has argued that despite mental health conditions having their first onset during adolescence and young adulthood, these conditions often go undetected.[5, 21, 25, 26] Adolescent mental health literacy empowers adolescents to recognise mental health symptoms and conditions, seek services, understand how they can improve their mental health, as well as combat stigma.[5, 21, 25] An appropriate package of services is key to overall quality of adolescent mental health care; it ensures that adolescents receive "adolescent-friendly", comprehensive (promotion, prevention, diagnosis and treatment) mental health care. Prior evidence has found that health services for adolescents have focussed on a limited range of services, such as sexual and reproductive

For peer review only

health, with the service not equipped to deliver mental services to adolescents.[5 21] At the centre of providing quality adolescent mental health care is provider competencies, which includes providers' knowledge, attitudes and skills, as well as the provision of evidence-based care.[21, 27, 28] Prior evidence has found that health care providers often do not have the technical competence to promote, prevent and manage adolescent mental health cases.[4]

Box 1. Definitions of Quality by Organisation and Criteria

**Organisations**	**Definition**	**Criteria**
Lancet Global Health Commission for High Quality Health Systems (2018)[16]	Quality health systems ensure that healthcare is optimised. This occurs by responding to population needs, providing care that maintains or improves health outcomes, and ensuring that all individuals feel valued	Quality framework components<br>• *Quality impacts*: better health, confidence in system, and economic benefit<br>• *Processes of care*: competent care and systems, positive user experience<br>• *Foundations*: population, governance, platforms, workforce, tools
WHO, Organisation for Economic Co-operation and Development (OECD), World Bank (2018)[29]	How health services ensure desired health outcomes for the population, using evidence-based knowledge	Foundations critical to high quality health services:<br>• *Healthcare workers*: motivated and supported<br>• *Healthcare facilities*: accessible and well-equipped<br>• *Medicines/Devices/Technologies*: safe in design and use<br>• *Information Systems*: continual monitoring<br>• *Financing mechanisms*: enable and encourage quality
National Academies of Sciences (2018)[17]		Six dimensions of quality<br>• Safety<br>• Effectiveness<br>• Person-centeredness<br>• Accessibility, Timeliness, Affordability<br>• Efficiency<br>• Equity

Despite a growing interest and investment in adolescent mental healthcare services, and the quality of such services, evidence on care quality and its effectiveness remain limited.[16] There is particularly poor information about how quality mental health services for adolescents should be developed and organised, clinicians trained, and health facility

For peer review only - http://bmjopen.bmj.com/site/about/guidelines.xhtml

interventions implemented to improve adolescents' mental health outcomes and that meet their needs.[30-32] This gap is particularly relevant for adolescents in adverse family and social circumstances.[9, 10] The objective of this systematic review is to evaluate the quality of adolescent mental health service provision globally, according to the WHO Global Standards of adolescent mental health literacy, provider competencies, and an appropriate package of services.[21] This review focusses on quantitative and mixed-method evaluations of mental health services for 10-to-19 year olds adolescents with suspected or diagnosed cases of depression, anxiety, and PTSD.

METHODS

The protocol was registered with PROSPERO (CRD42020161318) and is available online (https://www.crd.york.ac.uk/prospero/display_record.php?RecordID=161318) (supplemental material 1). For this systematic review, we used the WHO Global Standards for quality health care services for adolescents, focussing on standards for mental health services.[21]

Selection criteria

Inclusion criteria were quantitative and mixed-method studies that evaluated or assessed quality measures as defined by the WHO Global Standards (adolescent mental health literacy, provider competencies, and appropriate package of services) applied to existing mental health services targeted to adolescents (10-19 years) for depression, anxiety, and PTSD. We used *the Lancet Global Health Commission for High Quality Health Systems*[16] conceptual framework to choose three of the WHO quality Standards. Although all dimensions of the conceptual framework are relevant to a high-quality health system, we were most interested in focussing on the population that the health service is serving, the delivery of competent mental health care and systems and processes of providing care.[9] Depression and anxiety are the most common mental health outcomes in adolescents living in challenging environments.[33-38] PTSD is also associated with living in challenging environments. There is evidence that exposure to both interpersonal (e.g., assault, war terrorism and injury due to violence) and non-interpersonal (e.g., accidents, natural disasters, sudden death of a loved one, witnessing or hearing about death or death threats and life threatening diseases) trauma, characteristics typical of challenging environments, is associated with the development of PTSD.[8, 33-39] Furthermore, environments in which adolescents are more likely to experience adversities associated with these disorders are often in settings where quality mental health care is scarce.[9]

For peer review only

Articles that focussed on adolescent mental health literacy, appropriate package of services, and provider competencies were identified and classified according to the process and output criteria in the WHO report found in supplemental material 2.[21] Articles were included if adolescents had used or were currently using mental health services, or were exposed to interventions or strategies within established mental health services. Mental health services were defined as health services delivered at the primary, secondary, or higher health facility level that offered prevention and treatment for anxiety, depression or PTSD, as well as any community-based initiatives originating from these health service levels. Articles about healthcare providers delivering mental health services to adolescents were also included. No exclusions were made by country. In cases where studies included individuals aged 18-24 years or 0-10 years, inclusion criteria were met as long as 10-19 year olds were the primary population of the study, meaning >50% of the study population and the median being within the age range of interest.

While mental health services can be delivered through school-based health services, we restricted the search to more generic and specialised health care facilities. Transition services for adolescents to adult services were also excluded as we strictly focussed on 10-19 year olds. Clinical interventions that focussed solely on improving health outcomes were also excluded if they did not detail how quality measures were used. Mental health conditions linked to a comorbid physical health condition or learning disability were also excluded, given the more complex ways in which depression and anxiety can present in these groups, and the extent to which PTSD can reflect comorbid physical conditions in particular. We did not include schizophrenia spectrum disorders, bipolar disorders or adolescent precursors of these two severe groups of mental health conditions, as the median age of onset is older than the 10-19 year old age range and these disorders are less common than anxiety, depression and PTSD. We did not include studies that focussed on “at-risk mental states”, as evidence indicates that current risk identification approaches are limited within mental health services.[40] Our primary interest was public health services, which led to us excluding private healthcare services. Family therapy was also excluded as we wanted to focus on services that more directly targeted adolescents.[4]Given the launch of the *Mental health Gap Action Programme (mhGAP)* guidelines by the WHO in 2008[41] which highlights the large treatment gap globally, studies prior to 2008 were excluded.[42] No language restrictions were applied.

Search Strategy

The Preferred Reporting Items for Systematic Reviews and Meta-Analyses (PRISMA) methodology was used to select the articles (supplemental material 3 contains the PRISMA

For peer review only - http://bmjopen.bmj.com/site/about/guidelines.xhtml

checklist).[43] Peer-reviewed literature was searched through the following databases: Pubmed, PsycINFO, MEDLINE, EMBASE, and LILACS from 1 January 2008 to 31 December 2020. Articles were also found by screening references in selected scientific articles that matched eligibility for inclusion. The search strategy is provided in Table 2.

Table 2. Search strategy of electronic databases

Evaluation terms	Quality terms	Population	Setting
Evaluation	Quality	Adolescent	Mental health service
Assessment	Health literacy	Youth	Primary mental health service
	Appropriate package of services	Teen	Mental health counselling
	Provider competencies	Young People	General Practitioner (GP) service

Data Collection and Extraction

Titles and abstracts were exported to Endnote[44] and scanned for relevance by MQD. Articles were removed if they did not meet inclusion criteria or were duplicates. The full text of included articles was obtained and studies were classified in relation to: (i) mental health literacy; (ii) appropriate package of services; or (iii) provider competencies. Dual screening was conducted by two authors (MQD and KJR) to ensure that they met the inclusion criteria for this review.

Data Synthesis and Quality Assessment

A narrative synthesis of the included studies was undertaken as the lack of homogeneity precluded a quantitative synthesis of findings. The methodological quality of the studies was assessed using the National Institutes for Health (NIH) Study Quality Assessment Tools.[45] Studies were assessed for sources of bias (e.g., patient selection, performance, attrition, and detection), confounding, study power, and strength of causality in the association between interventions and outcomes.[45] Two reviewers (MQD and KJR) divided the studies in half (50/50) and rated each study independently. They then checked each other's coding for agreement. Disagreements were resolved through discussion with a third reviewer (LK). Based on the ratings of each component, each study received an overall rating of good, fair, poor. Extracted data were entered into a table of study characteristics, including the quality assessment ratings for each study (Table 3 and additional information found in supplemental material 4).

For peer review only

Patient and public involvement

Patients and the public were not involved.

RESULTS

Study Selection

Figure 1 shows the results of the search and selection strategy. Of 20,104 references identified, 456 full-text articles met inclusion criteria from which a total of 20 articles were included in the study.

Figure 1. Flow diagram of studies

For peer review only

Table 3. Characteristics of included studies

Author and country	Type of mental health service	Study/Evaluation design	Target population	Element(s) of quality addressed	Results	Quality assessment
Davidson et al (2019),[46] USA	Secondary level services	Pilot randomized controlled trial (RCT)	n=18 providers<br><br>n=32 children and adolescents (5-16 years, mean=11.5 years)	Appropriate package of services; Provider competency	Strong alliance between adolescents and providers, d=0.11; Assisted in skills-based learning, d=0.47; Adolescent satisfaction with treatment (Child/Adolescent Satisfaction Questionnaire), d=0.53	Fair
Jager et al (2017),[47] The Netherlands	Secondary level services	Longitudinal prospective cohort	n=315 12-18 year olds	Appropriate package of services; Provider competencies	Smaller reduction of psychosocial problems when adolescents valued communication but didn't experience it (total difficulties score at T1 an average of 15.6 and at T2 13.9)	Fair
Ougrin et a (2018),[48] UK	Tertiary level services	RCT	n=53 in intervention arm (0-17 years; mean=16 years); n=53 in control arm (0-17 years; mean=16 years)	Appropriate package of services	Lower occupied bed days between intervention and control arm (median 34 days, p=0.04); Intervention arm more effective and financially reasonable compared to control arm	Good
Dion et al (2010),[49] Canada	Emergency department (ED) services	Cross-sectional surveyed	n=87 medical staff (nurses, residents, and physicians)	Provider competency	ED staff reported greater confidence in managing and referring patients based on years of employment (r=0.35, p<0.01); Physicians (83%) were more confident than nurses (8%) or residents (8%) (p=0.05); The	Fair

For peer review only - http://bmjopen.bmj.com/site/about/guidelines.xhtml

					majority of ED staff (67% of nurses, 64% of residents, and 83% of physicians) were satisfied with the program	
Spenser et al (2009),[50] Canada	Tertiary level services	Cohort study	n=27 community paediatricians; n=16 outpatient mental health clinicians	Provider competency	5 mental health clinicians stated that having paediatricians on mental health team was positive; Over 20% of clinical activity for almost 12 paediatricians deals with mental health issues; 7 mental health clinicians stated that the educational sessions led to increased knowledge about mental health of children and adolescents	Poor
Ayton et al (2013),[51] UK	Secondary level services	Mixed methods including survey with consultants and review of case notes.	n=23 Child an Adolescent Mental Health Service (CAMHS) consultants surveyed; 33 case notes reviewed	Appropriate package of services; Provider competencies	Care Programme Approach (CPA) care plans audited assessed 96.4% of mental health and needs and 71.4% of family needs; other needs (housing: 33.9%, sexual health: 7.1%, and sign of relapse or crisis: 28.6%) minimally assessed in CPA plans	Fair
Aupont et al (2013),[52] USA	Primary level services	Prospective cohort with adolescent service users	n=329 (m=12.3 years)	Appropriate package of services	The relative risks of staying in mental healthcare (instead of going back to paediatrics) was 7.5 for those with depression and 5.1 for those with anxiety disorders; The return rates to the referring paediatrician were 27.9% and 5.9% for adolescents with anxiety and major depressive disorders	Fair
Simmons et al (2016),[53] Australia	Enhanced primary level services	Prospective cohort study	n=57 12-25 year olds used the decision aid	Mental health literacy; Provider competencies	97% reported increased confidence in deciding about their own healthcare after using the decision aid (p=0.022); on shared decision making	Fair

For peer review only - http://bmjopen.bmj.com/site/about/guidelines.xhtml

			and completed the post-decision assessment; n=48 completed the follow-up assessment		adolescents scored average of 37.4 (range 29-44); indicating high level of perceived involvement in the treatment process	
Irvine (2020),[54] Ireland	Community, primary, secondary and tertiary level services	Cross-sectional online survey	n=604 11-21 year olds	Mental health literacy, appropriate package of services, provider competencies	73% of adolescents stated that they had been spoken to in a way that they could understand; 42% and 40% stated that they were given a choice in treatment/support and felt involved in their treatment decisions; GP (General Practitioner) and ED services scored poorly across all quality indicators; community services scored the highest	Fair
Jager et al (2014),[55] the Netherlands	Secondary level services	Longitudinal, prospective cohort study	n=211 12-18 year olds	Appropriate package of services; Provider competencies	Adolescents who valued patient centred communication but did not have their communications needs met were less likely to adhere to their treatment (OR: 2.8; CI: 1.1-6.8)	Fair
Stevens et al (2009),[56] USA	Tertiary level services	RCT	n=179 11-20 year olds	Appropriate package of services	No significant differences found between treatment and control arms	Good
Anderson et al (2012),[57] Australia	Secondary level services	RCT	n=73 12-18 year olds	Appropriate package of services	Adolescents in intervention (mean 5.77, SD 1.2) and control (mean 5.58, SD 1.34) reported strong working alliance; Adolescent working alliance was positively associated with compliance at 6 month follow-up (r=0.30, p<0.001)	Poor

For peer review only - http://bmjopen.bmj.com/site/about/guidelines.xhtml

Kapp et al (2017),[58] Switzerland	Secondary level services	Cross-sectional study	n=663 patients 10+years of age	Appropriate package of services	Patients who had time to formulate and ask questions had better alliance (p<0.001); Easy accessibility to CAMHS by phone had higher alliance scores (p=0.037)	Fair
Cairns et al (2019),[59] Australia	Primary level services	Cohort	n=283 clinical charts of 12-25 year olds	Appropriate package of services; Provider competencies	Emotional management and well-being goals were most frequently recorded; None of the analysed goals met criteria for being specific, measurable, and timed; 57% were specific while 14% were measurable; none had a timeframe; Goal quality was not associated with service retention	Fair
Ringle et al (2019),[60] USA	Secondary level services	Medical record audit	n=727 medical records of 8-18 year olds	Provider competencies	46% of children and adolescents received care that was guideline concordant; Clients with worse functioning (*OR* = 0.985, p < .001), higher problem severity (*OR* = 1.02, p = .015), higher risk of harm to others (*OR* = 1.61, p < .001), more school problems (*OR* = 1.48, p < .001), and who had a diagnosis of depression (*OR* = 1.37, p < .05) or a conduct-related disorder (*OR* = 1.37, p < .05) at intake were more likely to receive less intensive services than those recommended by the guidelines.	Fair
Sattler et al (2019),[61] USA	Mix of primary and secondary level	Medical record audit	n=694 medical records 7-17 year olds	Appropriate package of services; Provider competencies	Patients received 1.48 evaluations on average for psychiatric symptoms; 45.7% of all facilities used self-report measures and 5.2% used diagnostic interview; 23.2% of psychologists documented the use of diagnostic	Fair

For peer review only - http://bmjopen.bmj.com/site/about/guidelines.xhtml

					interviews, compared to 2% and 0% of psychiatrists and primary care physicians (p<0.01); 43% psychologists, 43.3% psychiatrists, and 39.3% of social workers more likely to document specific diagnoses compared to primary care (22.6%) (p<0.01)	
Sattler et al (2016),[62] USA	Primary and secondary level services	Medical record audit	n=801 7-17 year olds	Appropriate package of services; Provider competencies	5.3% of anxiety disorder speciality clinics used structured diagnostic interviews; 21% of all health facilities used rating scales (28.9% of speciality clinics, 19.6% of general mental health clinics, and 15% primary care); Evaluations in specialised clinics resulted in specific diagnosis (p<0.001); rating scales were associated with specific diagnosis (p=0.04)	Fair
Higa-McMilan et al (2017),[63] USA	Secondary level services	Medical record audit	n=2,485 3-19 year olds	Appropriate package of services; Provider competencies	55-93% of cases use the following Practices Derived from the Evidence Base (PDEB) for adolescents: cognitive, psychoeducational, relaxation, modelling; 99.7% of youth had at least one PDEB over their treatment course	Fair
Rukundo et al (2020),[64] Uganda	Primary, secondary and tertiary level	Clinical record review	n= 50 providers	Appropriate package of services; Provider competencies	Since training: more children and adolescents had patient-centred assessments; decreased use of medication with more appropriate medication prescribed; increased use of psychological treatments; and non-CAMH professionals had greater diagnosis revisions and management of cases	Fair

For peer review only

Bardach et al (2020),[65] USA	Emergency department	Medical record review	n=22,844 children and adolescents 6-17 years of age (majority 12-17 years)	Appropriate package of services; provider competencies	62% and 82.3% of patients had follow-up within 7 days and 30 days, respectively; patients discharged from GPs and EDs were less likely to have follow-up compared to those discharged from psychiatric services; Follow-up within 7 or 30 days of discharge was associated with an increased risk of a subsequent hospitalization or ED visit for a mental health illness	Fair

Acronyms: randomized controlled trial (RCT); emergency department (ED); Child and adolescent mental health services (CAMHS); Care programme approach (CPA); Practices derived from the evidence base (PDEB); General Practitioner (GP)

For peer review only

Study Characteristics and Settings

The majority of studies were conducted in high-income countries, namely the USA (8 studies),[46, 52, 56, 60-63, 65] Australia (3 studies),[53, 57, 59] United Kingdom (2 studies),[48, 51] Canada (2 studies),[49, 50] the Netherlands (2 studies),[47, 55] Ireland,[54] and Switzerland (1 study).[58] One study was conducted in Uganda.[64] Most of the studies focussed on evaluating or assessing the provision of services that are appropriate for adolescents (15 studies) and enhancing provider competencies (15 studies), with a minority focussed on increasing adolescents' mental health literacy (2 studies).

The services ranged from emergency,[49, 65] to primary level;[52-54, 59, 61, 62, 64, 65]secondary level mental health services;[46, 47, 51, 54, 55, 57, 58, 60-65] and tertiary level services.[48, 50, 54, 56, 64, 65]

Quality Assessment

The majority of the studies were assessed as 'fair' quality (n= 16, 80%); 2 (10%) studies were assed as 'good' quality, and 2 (10%) were assessed as 'poor quality'. Studies rated as 'poor' mainly had lack of clarity about the methods and outcomes analysed, confounding and higher sources of bias. Ougrin and colleagues (2018) and Stevens and colleagues (2009) implemented studies that were rated as good quality.[48, 56] Both conducted randomised controlled-trials in tertiary level facilities. They also experienced low drop-out rates, high adherence to the interventions, and consistently used valid and reliable measures.[48, 56]

Conceptualising Quality

The majority of studies did not conceptualise quality.[47-53, 55-57, 64] Where it was conceptualised, it was in reference to high quality care, defined as healthcare provider fidelity to evidence-based treatment models and adolescents' engagement in the treatment process (satisfaction and quality of engagement with therapists and adolescents);[46] as quality indicators in terms of information and access, facilities and services and quality of care;[54] as quality indicators in child and adolescent mental health services, specifically around patient satisfaction and quality engagement between the therapist and adolescent;[58] or follow-up after hospitalisation for a mental illness.[65] Common themes within these studies were a focus on the processes of care and quality impacts (improved mental health and greater confidence of the health service and system).[16]

Other studies mentioned quality in relation to patient-centred communication or how providers adapt their communication style to meet the needs and preferences of their

For peer review only - http://bmjopen.bmj.com/site/about/guidelines.xhtml

patients.[55] Quality was also considered in relation to goal setting between the therapist and adolescent patient and whether these goals were specific, measurable, achievable, realistic/relevant, and timely.[59] The use of evidence-based assessments, practices, and policies was another aspect of quality mentioned in some studies[60-62, 64]. Other aspects of quality were linked to communication. These studies included: coordination of care between an in-patient mental health unit and a community service;[51] mental health care delivered through the emergency department;[49] and collaboration between family physicians and psychiatrists.[50] Jager and colleagues (2017) measured affective quality as a key component of patient-centred communication, however, this was not defined.[47] The common theme that emerged from these studies is that they focussed on foundations of quality care.[16] Findings from the review are presented below by WHO quality standards.[21]

Quality Standards

Adolescent Mental Health Literacy

Two studies reported outcomes relevant to adolescent mental health literacy. One focussed on an online decision aid for mental health services.[53] 97% of adolescents reported increased confidence and awareness in deciding about their own healthcare and involvement in the treatment process when exposed to an online decision aid (p=0.022).[53] Another evaluated adolescents' experience with mental health services; specifically, whether they were given useful information to understand their mental health needs and if they had a choice in their treatment and/or support.[54] EDs and GPs scored poorly on these measures (<50% of adolescents experiencing this) while more than 50% of adolescents reported that they had experienced this in community CAMHS and in-patient care.[54] The quality of evidence from these studies was fair.

Appropriate Package of Services

Fifteen of the 20 studies evaluated services that met the WHO standard for appropriate packages of services.[21] Interventions that involved aspects of an appropriate package of mental health services were diverse and targeted the quality of engagement between the therapist and adolescent [46, 54, 57-59] patient-centred communication,[47, 55] mental health service use,[52, 56, 64] linkages to mental health services,[52] health facility culture and patient safety,[51] clinician's assessment of diagnostic and treatment services,[61, 62]

For peer review only - http://bmjopen.bmj.com/site/about/guidelines.xhtml

and intensive community treatment.[48] The quality of the evidence for the 15 studies was poor to good.

Five of the 15 studies[46, 54, 57-59] reported improvements in the quality of engagement between therapist and adolescent patient, including the interaction, collaboration, and bond[57]. These included the use of a tablet-based application on trauma-focused cognitive behavioural therapy (TF-CBT) in the US,[46] an online cognitive behaviour therapy intervention in Australia,[57] an outpatient child and adolescent mental health clinic in Switzerland,[58] and the use of goal setting in Australia.[59] Davidson and colleagues (2018) evaluated a tablet-based application on TF-CBT, finding a small to medium effect size on developing therapeutic tasks (d=0.47) and a small effect size on therapeutic bond (d=0.11), with most adolescents satisfied with the intervention (d=0.53).[46] Anderson and colleagues (2012) evaluated an online cognitive behaviour therapy intervention with minimal therapist contact, finding that greater therapeutic alliance led to greater adolescent adherence with treatment at 6 month follow-up (r=0.30, p<0.001). Results also found that adolescents in both the intervention and control arm reported strong therapeutic alliance.[57] Irvine (2020) evaluated adolescents' experience at different types of health facilities; specifically, whether they felt involved in the decisions that were made about their treatment plan and if they found the support to be helpful;[54] 16%, 30%, 36% and 42% of adolescents stated that they felt involved in decisions about their care from in-patient facilities, EDs, community CAMHS and GPs, respectively, and 34%, 39%, 44% and 45% felt the support they received was helpful from EDs, in-patient care, GPs and community CAMHS respectively.[54]

Two of the 15 studies[47, 55] focussed on patient-centred communication in the Netherlands, finding that adolescents who did not experience patient-centred communication were less likely to adhere (OR: 2.8, CI: 1.1-6.8) and have confidence (OR: 4.5, CI: 1.8-11.6) in their course of treatment.[55] They also were less likely to experience a significant reduction in their mental health problems, compared to those who experienced patient-centred communication.[47]

One study evaluated mental health service use through a telephone support service (TSS) intervention in the US.[56] The study found no difference in mental healthcare utilisation (p=0.65) between adolescents in the intervention and those in usual care.[56] Another study evaluated referrals between paediatric care to mental health services through the Targeted Child Psychiatric Service (TCPS) program in the US. The program enables access to specialised mental health services for adolescents with mental health conditions from paediatric primary care, ensuring long-term management in the most appropriate healthcare

For peer review only - http://bmjopen.bmj.com/site/about/guidelines.xhtml

setting. Results showed that adolescents with depression and anxiety required continued access to specialised mental healthcare.[52]

One study[51] evaluated health facility culture and patient safety with the Care Programme Approach (CPA), which ensures that children and adolescent patients are involved in all aspects of their mental health care in the UK. Patient safety was found to be an issue. In fact, unplanned discharge (i.e., self-discharge, tribunal discharge, or commissioning pressure to discharge) was the most common problem due to siloed rather than collaborative, decision-making. Other challenges were limited collaboration between early intervention, education, and CAMHS teams; and a lack of joint protocols on the CPA and discharge between organisations.[51]

Two studies in the US focussed on the use of structured interviews, symptom rating scales, and Diagnostic and Statistical Manual of Mental Disorders (DSM-IV-TR) diagnostic criteria in primary, secondary, and tertiary level services.[61, 62] Results showed that structured diagnostic interviews were more likely to be used by psychiatrists and psychologists compared to general physicians ($p<0.01$).[61]

One study examined whether a Supported Discharge Service (SDS), or intensive community treatment, would be more beneficial and cost-effective than usual care among adolescents.[48] A significant difference in the overall number of bed-days from the SDS arm was found at 6 months (median 34 days, $p=0.04$) compared to usual care. The SDS was found to have at least 50% probability of being cost-effective in comparison to usual care and willingness to pay for outcome improvements (the incremental cost effectiveness ratio was -£991), or the cost per life year gained.[48]

Providers' Competencies

Fifteen studies included outcomes relevant to healthcare provider competency. They focussed on confidence in managing and referring adolescents with mental health issues,[49] greater collaboration between paediatricians and mental health clinicians,[50] use of evidence-based practices,[60-63 65] use of a tablet to facilitate adolescent patient engagement in therapy,[46] provision of information,[47, 53-55] and implementation of care models and plans.[51, 52, 64] The quality of evidence for the fifteen included studies ranged from poor to fair.

In evaluating confidence of referring adolescents with mental health patients, Dion and colleagues (2010) found that training through a Crisis Intervention Program increased ED

For peer review only - http://bmjopen.bmj.com/site/about/guidelines.xhtml

staff confidence in managing and triaging patients (r=0.35, p<0.01) in Canada.[49] Another study evaluated collaboration between paediatricians and outpatient mental health clinicians in Canada. Results showed a positive effect on patient care with a paediatrician on the mental health team.[50]

Five studies evaluated the use of evidence-based practices and guidelines in adolescent mental health services.[60-63] Higa-McMillan and colleagues (2017) found that the most commonly used evidence-based practices for adolescents with anxiety disorders included cognitive, psychoeducational, relaxation, and modelling.[63] In a US study, the authors found that patients were more likely to receive less intensive services if they had poorer functioning, greater problem severity, greater risk of harm to others, and greater school problems, with a diagnosis of depression or conduct disorder than guideline recommended.[60] As part of the Children's Core Set of quality measures, Bardach and colleagues (2020) evaluated the follow-up after hospitalisation for mental illness at 7 and 30 days for children and adolescents (aged 6-17 years old) in the US. Results showed that 62% and 82.3% of patients were followed-up within 7 and 30 days. Adolescent patients were more likely to be followed-up after discharge from psychiatric units and hospitals compared to those from general medical or surgical units.[65]

A two-year child and adolescent mental health training programme for health care providers (psychiatric clinical officers, psychologists, psychiatric nurses, general nurses, occupational therapists, etc) was implemented in Uganda.[64] Medical records were reviewed annually over six years, finding that a greater number of children and adolescents were receiving thorough patient-centred assessments; a reduction in medication prescription; an increase in the use of psychological treatments and greater management of cases by non-CAMHS professionals after the intervention.[64]

Two studies in the Netherlands found that adolescents who did not experience patient-centred communication were less likely to understand (OR: 3.7, CI: 1.5-9.0) [47 55](OR: 3.1, 1.1-8.5) [55] their course of treatment. Similarly, through the use of an online decision aid, 93% of participants were more likely to make a healthcare decision that was guideline concordant (p=0.004) and consistent with their preferences.[53]

DISCUSSION

To our knowledge, this is the first review that has attempted to evaluate the quality of adolescent mental health services.[21] A total of 20 studies were identified, overwhelmingly

For peer review only - http://bmjopen.bmj.com/site/about/guidelines.xhtml

from high-income countries. Fifteen studies focussed on packages of services, 15 on healthcare provider competency and two on mental health literacy. There was limited evidence[53] of an intervention improving mental health, however, we cannot conclusively state this was effective.

Despite the large contribution of mental health conditions to the global burden of disease in adolescents and the need for quality mental healthcare services, we found that most studies lacked a formal conceptualisation of quality and did not have a clear framework or definition of quality. There were a variety of instruments used to measure quality and its indicators. Our understanding of quality in mental health services, as well as the generalisability of our findings, is therefore limited. Our findings also indicate a large service gap and suggests that there is a need to not only develop and standardise a definition of what constitutes quality adolescent mental healthcare, but also develop and standardise methods that measure quality in adolescent mental healthcare.

It should be noted that the WHO Global Standards were developed through a rigorous process.[42] This involved a needs assessment, the development of the Standards, consultations with experts, assessing the usability of the Standards through regional consultations and a country field test.[42] The WHO includes an implementation guide at the national, district and facility levels that identify actions needed to implement the Standards.[21] The majority of studies were conducted by universities in health facility settings. In reviewing the implementation guide, health care provider training and use of decision support tools at the health facility were the actions most relevant.[21] However, it was unclear the level of involvement of the health facility manager in the studies and whether there was an uptake of the intervention by the health facility after study completion. At the same time, there is a lack of peer-reviewed evidence on the implementation and evaluation of these Standards, as well as a lack of specific and contextualised indicators to evaluate and monitor the Standards. This illustrates a gap between the literature and the Standards. The WHO (2015) does recognise that not all Standards will be implemented, and that the Standards were made to be evaluated and developed further once adapted and implemented at the national and regional levels.[42]

There are several challenges to providing quality care within adolescent mental health services, including stigmatising attitudes and behaviours about treatment seeking, service provision and utilisation, [66-69] the lack of professional expertise, [22-24] and the current disease-based model of medicine.[20] Also, quality in mental health services has received little attention in relationship to adolescents.[16] Indeed, stigma is a significant barrier to the

For peer review only - http://bmjopen.bmj.com/site/about/guidelines.xhtml

availability and delivery of quality mental health services within communities.[13] It has been posited that stigma occurs at the structural (organisation, resources, quality standards), interpersonal (the quality of engagement between the healthcare provider and adolescent, patient safety), and intraindividual levels (healthcare providers unwilling to assess adolescent mental health conditions, adolescents unwilling to seek mental healthcare services).[70, 71] This failure in quality prevents adolescents from seeking and continuing care for mental health conditions due to perceived stigma. [70, 72, 66-69] It points to a need for mental health literacy among healthcare providers. Quality mental health services cannot be achieved without healthcare providers having a reasonable understanding of adolescent mental health. This needs to start with healthcare provider training and preservice education.[72]

Apart from one study from Uganda,[56] all of these studies were from high-income countries, illustrating an important gap in the literature within LMICs (low- and-middle-income countries) around quality of adolescent mental health services. This could reflect service and research gaps in all aspects of mental health in LMIC,[73-75] as well as different ways in which mental health is conceptualised in LMICs at the national and local levels[20]. It may also reflect the continued orientation in LMICs to more acute health conditions rather than in response to complex conditions that require long-term care, such as mental health conditions.[16, 20] Patel et al (2019) argue that mental health conditions do not follow the typical disease-based model of medicine, and that a 'one size fits all' approach does not work.[20] This is particularly the case for subsyndromal or early onset mental health conditions which may not readily fit with diagnostically oriented services.[69]

There have been efforts to overcome these challenges, as identified in table 4. While the majority of these studies focussed on generic healthcare for adolescents, their findings are equally relevant for adolescent mental health care.

For peer review only - http://bmjopen.bmj.com/site/about/guidelines.xhtml

Table 4. Frameworks to Address Quality in Adolescent Health Services

Reference	Framework	Components of Framework
Sawyer et al (2014)[76]	Conceptual framework for adolescent-friendly healthcare based on experience of care and evidence-informed care, including a set of 14 Indicators of quality healthcare for adolescents in hospitals	*Experience of care:*
		Felt welcome in hospital
		Age appropriate environment
		Respected by clinicians
		Trust in clinicians
		Understanding of health information
		Involvement in decisions about care or treatment
		Comfort asking questions about health and wellbeing
		Evidence informed care:
		Psychosocial assessment
		Confidentiality discussions
		Time alone in consultations
		Self-management
		Transfer to adult services
		Supported to continue education
		Connection to external supports
Ambresin et al (2013)[28]	Domains for youth-friendly care to assess how well services are engaging young people	Accessibility of healthcare
		Staff attitude
		Communication
		Medical competency
		Guideline-driven care
		Age-appropriate environment
		Involvement in healthcare
		Health outcomes
UK NHS (2007)[77]	'You're Welcome' quality criteria to ensure that health services (primary, community, specialist and acute)are young people-friendly	Accessibility
		Publicity
		Confidentiality and consent
		The environment
		Staff training, skills, attitudes and values
		Joined-up working
		Monitoring and evaluation and involvement of young people
		Health issues for adolescents
		Sexual and reproductive health service
		Child and adolescent mental health services
Sayal et al (2012)[32]	10 quality standards for children and adolescents in primary mental healthcare	Confidentiality
		Knowledge
		Awareness
		Communication
		Continuity of care
		Access and referral

Arguably, a good starting point for measuring quality in adolescent mental healthcare services would be a more scalable combination of the youth-friendly guideline driven care

For peer review only - http://bmjopen.bmj.com/site/about/guidelines.xhtml

developed by Ambresin and colleagues (2013) and the quality standards developed by Sayal and colleagues (2012). The same investments that promote quality in other age groups will be similarly valuable for adolescents, but greater specificity and focus is required around the health service aspects of engagement, communication, and confidentiality. The studies identified in our review examined aspects of engagement and communication, but interestingly, did not explore confidentiality.

The findings from our review should be appreciated in light of the broader challenges to quality in adolescent mental health services as described above. Regarding adolescent mental health literacy, we found that a youth decision aid helped young people make evidence-informed decisions about their treatment, feel engaged in the process, and increased treatment adherence.[53] Previous literature has found similar results, with patients reporting increased involvement in treatment decision-making, increased knowledge about the treatment options and outcomes, and greater comfort making decisions.[78] As a foundation for quality, adolescents' knowledge shapes the way mental health services respond to them, and helps adolescents hold these services to account.[16] For packages of services, the quality of engagement between the adolescent and therapist was found to lead to positive outcomes.[79, 80] The quality of the patient-therapist relationship has led to greater treatment efficacy,[81] increased autonomy, patient alliance and engagement, and greater favourable outcomes.[57, 82] It ensures that the adolescent's perspective is included, that they consent and assent to their treatment plan,[83] and that they can address problems throughout the treatment process.[84] Evidence from the UK has shown that current services are not adequate for young people's mental health needs,[85-87] with youth reporting that they should be more engaged in the design of mental health services. From the studies on provider competency, we found that training general healthcare providers about adolescent mental health conditions helped build their confidence and knowledge when treating adolescents.[49, 50, 64] This aligns with previous literature as healthcare providers reported confidence, knowledge, and a lack of specialised providers as barriers to care.[88, 89] Provider competency is a foundation and process of care.[16] Adolescent mental healthcare providers require adequate clinical education and training on adolescent mental health. They also should provide evidence-based treatment, communicate clearly, ensure confidentiality and autonomy, promote timely and effective care and instil confidence in their adolescent patients that their conditions are being correctly detected and managed.[16]

The quality of evidence, assessed using the NIH Quality Assessment Tool, ranged from poor to good, and included various limitations in study design which could bias the results of the

For peer review only - http://bmjopen.bmj.com/site/about/guidelines.xhtml

review. Furthermore, there was variation in the approach and tools used within evaluations, the content of the service, as well as the sample size. Certainly, within the three standards we systematically reviewed, it is not possible to identify the most effective standard of quality or service delivery method, as conforms to the WHO global standards.[21]

Recommendations

We have several recommendations for further research on quality in adolescent mental health services to promote improved mental health outcomes. These recommendations are particularly important in light of the reported increase in adolescent mental health conditions associated with the COVID-19 pandemic.[90-94]

- First, to promote comparability, understanding and inform data collection, agreement around a developmentally-appropriate definition of quality would inform methods to measure quality in adolescent mental health services across different contexts.
- Second, collaborative research efforts, including the active participation of adolescents in this process, are needed to strengthen the evidence on quality in adolescent mental health services, especially in LMICs. This includes research on adolescent mental health needs (particularly those exposed to daily adversity), research that articulates and tests the types of services that are best able to respond to their needs, knowledge of effective strategies to improve the quality of mental health services, including efforts to upskill the capabilities of all healthcare providers, not just mental health professionals, around adolescent mental health,[95] and evidence on the sustainability and effectiveness of what quality interventions to scale up through the health system, including psychotropic drugs and telehealth interventions. Furthermore, future studies should focus on psychotic disorders, which are also prevalent among adolescents and for which little evidence on quality of care is available.
- Third, health services need to proactively engage adolescents about their health needs, including mental health needs, and to ensure that they are informed about confidential services that are available to them, including vulnerable and at-risk adolescents.
- Fourth, to reduce stigma and close the well described treatment gap [96-98], there needs to be greater investment in integrating adolescent mental health services into primary healthcare and training of non-specialised health care providers on adolescent mental health. This review shows that stigma negatively influences the quality of adolescent mental health services, which affirms the value of incorporating stigma reduction indicators into quality of care measures, as advocated by Knaak and colleagues (2015).[92] Cost-effectiveness analyses could help inform governments about the

For peer review only - http://bmjopen.bmj.com/site/about/guidelines.xhtml

benefits to be gained when better mental health is reflected in higher school completion and regular employment.

Study Limitations

This review should be interpreted within the context of a number of limitations. We recognise that the WHO Global Standards quality framework is but one way of categorising quality. Leslie and colleagues (2018) warn that despite recent initiatives and greater focus on quality in healthcare, the various concepts and frameworks used to define and measure quality have led to "inconsistent assessments and incomparable investments, leaving researchers and policymakers without direction".[99] We recognise that beyond mental health services, the focus of this review, that there are various resources that can address adolescent mental health. This includes parenting interventions, which have been found to improve the mental health of adolescents[94] and school-based mental health interventions, which have also been found to contribute to improved health.[22] We also appreciate that an important aspect of quality care is continuity of care. For adolescents with persisting mental health issues, the transition from child or adolescent oriented services to adult oriented mental health services is a particularly important aspect of continuity of care. While this was beyond the scope of this review, it is consistent with many adolescent services now using an extended definition of adolescence[100] up to 24 years.

None of the included studies focussed on the quality of psychotropic drug prescriptions or the use of telehealth, as this was beyond the scope of the review and a limitation. Psychotropic drug prescriptions are one component of treatment for severe adolescent mental health conditions, with evidence from the US in 2013 finding that 7% of adolescent participants were treated with psychotropic medication.[101] Despite this, high quality evidence on the long-term effectiveness and safety of these medications for adolescents is limited, varying by condition and medication class.[101-103] The use of telehealth for mental health conditions has a long history, but has been growing in recent years as an accessible, efficient, and cost-effective alternative to face-to-face consultations.[104] Evidence has found that telehealth is associated with patient satisfaction and is effective in evaluating and analysing mental health conditions.[104] In our review, we identified a number of studies that used different elements of telehealth. More recently, there has been appreciation of the particular benefits of telehealth within the context of the COVID-19 pandemic, including in low-resource settings. Evidence has found that it is associated with reduction in stigma and a higher participation rate among this age group, which may reflect adolescents ease with technology.[4, 105-107] However telehealth is also not without challenges, including around

For peer review only - http://bmjopen.bmj.com/site/about/guidelines.xhtml

privacy, confidentiality, safety and equitable service use.[105, 106] Limited access to the internet is a particular challenge to equitable telehealth. Telehealth sessions are typically easier to record which, in the context of informed consent, provides one mechanism to assess quality. Further research to identify which patients would most benefit from in-person visits or telehealth is indicated for common mental health conditions.[106, 108]

Conclusions

This review indicates the lack of consensus on quality in mental health services, with most of the identified studies failing to conceptualise quality at all. Many challenges remain around improving the quality of mental health care for adolescents.

For peer review only

Funding statement. This research received no specific grant from any funding agency in the public, commercial or not-for-profit sectors. Kathryn J Roberts is supported by an Economic Social Research Council (ESRC) PhD studentship through the UBEL DTP (kr0001).

Competing Interests. The authors have nothing to disclose.

Contributors. MQD led the study design, data searches, data extraction, quality appraisal, synthesis and drafted the manuscript. KJR contributed to the design, data extraction, quality appraisal and writing of the manuscript. LK and DD provided significant and critical contribution to the conceptualisation, design and interpretation of findings. SS critically reviewed the manuscript and offered key intellectual input to the synthesis and interpretation of findings. RC critically reviewed the manuscript and offered important intellectual input.

Data Availability Statement

No additional data available.

Ethical Approval Statement

Not applicable.

References

For peer review only - http://bmjopen.bmj.com/site/about/guidelines.xhtml

1. Kessler RC, Amminger GP, Aguilar-Gaxiola S, et al. Age of onset of mental disorders: a review of recent literature. *Current Opinion in Psychiatry* 2007;20(4):359-64. doi: 10.1097/YCO.0b013e32816ebc8c
2. Jones PB. Adult mental health disorders and their age at onset. *Br J Psychiatry Suppl* 2013;54:s5-10. doi: 10.1192/bjp.bp.112.119164 [published Online First: 2013/01/11]
3. Mokdad AH, Forouzanfar MH, Daoud F, et al. Global burden of diseases, injuries, and risk factors for young people's health during 1990–2013: a systematic analysis for the Global Burden of Disease Study 2013. *The Lancet* 2016;387(10036):2383-401. doi: 10.1016/S0140-6736(16)00648-6
4. Patton GC, Sawyer SM, Santelli JS, et al. Our future: a Lancet commission on adolescent health and wellbeing. *The Lancet* 2016;387(10036):2423-78. doi: 10.1016/S0140-6736(16)00579-1
5. WHO. Health for the world's adolescents: A second chance in the second decade. Geneva: World Health Organization 2014.
6. Kessler RC, Berglund P, Demler O, et al. Lifetime prevalence and age-of-onset distributions of DSM-IV disorders in the National Comorbidity Survey Replication. *Arch Gen Psychiatry* 2005;62(6):593-602. doi: 10.1001/archpsyc.62.6.593 [published Online First: 2005/06/09]
7. IHME. Global Burden of Disease Compare: 2016 Prevalence Seattle: Institute for Health Metrics and Evaluation; 2016 [Available from: https://vizhub.healthdata.org/gbd-compare/.
8. Alisic E, Zalta AK, van Wesel F, et al. Rates of post-traumatic stress disorder in trauma-exposed children and adolescents: meta-analysis. *Br J Psychiatry* 2014;204:335-40. doi: 10.1192/bjp.bp.113.131227 [published Online First: 2014/05/03]
9. Kalisch R, Baker DG, Basten U, et al. The resilience framework as a strategy to combat stress-related disorders. *Nature Human Behaviour* 2017;1(11):784-90. doi: 10.1038/s41562-017-0200-8
10. Global, regional, and national incidence, prevalence, and years lived with disability for 301 acute and chronic diseases and injuries in 188 countries, 1990-2013: a systematic analysis for the Global Burden of Disease Study 2013. *Lancet* 2015;386(9995):743-800. doi: 10.1016/s0140-6736(15)60692-4 [published Online First: 2015/06/13]
11. Yin X, Guven N, Dietis N. Stress-based animal models of depression: Do we actually know what we are doing? *Brain Res* 2016;1652:30-42. doi: 10.1016/j.brainres.2016.09.027 [published Online First: 2016/09/25]
12. de Vries GJ, Olff M. The lifetime prevalence of traumatic events and posttraumatic stress disorder in the Netherlands. *J Trauma Stress* 2009;22(4):259-67. doi: 10.1002/jts.20429 [published Online First: 2009/08/01]
13. Kessler RC, Aguilar-Gaxiola S, Alonso J, et al. Trauma and PTSD in the WHO World Mental Health Surveys. *Eur J Psychotraumatol* 2017;8(sup5):1353383-83. doi: 10.1080/20008198.2017.1353383
14. UNICEF. COVID-19 and children: UNICEF data hub NYC UNICEF; 2020 [Available from: https://data.unicef.org/covid-19-and-children/#
15. Horesh D, Brown AD. Traumatic stress in the age of COVID-19: A call to close critical gaps and adapt to new realities. *Psychol Trauma* 2020;12(4):331-35. doi: 10.1037/tra0000592 [published Online First: 2020/04/10]

For peer review only - http://bmjopen.bmj.com/site/about/guidelines.xhtml

16. Kruk ME, Gage AD, Arsenault C, et al. High-quality health systems in the Sustainable Development Goals era: time for a revolution. *Lancet Glob Health* 2018;6(11):e1196-e252. doi: 10.1016/s2214-109x(18)30386-3 [published Online First: 2018/09/10]
17. IOM. Crossing the Quality Chasm: A New Health System for the 21st Century. Washington (DC): National Academies Press (US)
Copyright 2001 by the National Academy of Sciences. All rights reserved. 2001.
18. WHO. Quality of care: a process of making strategic choices in health systems. Geneva: World Health Organization 2006.
19. WHO. Making health services adolescent friendly: Developing national quality standards for adolescent friendly health services. Geneva: World Health Organization 2012.
20. Patel V, Saxena S. Achieving universal health coverage for mental disorders. *BMJ (Clinical research ed)* 2019;366:l4516-l16. doi: 10.1136/bmj.l4516
21. WHO, UNAIDS. Global standards for quality health care services for adolescents: a guide to implement a standards-driven approach to improve the quality of health care services for adolescents. Geneva: World Health Organization, 2015.
22. Rickwood D, Deane FP, Wilson CJ, et al. Young people's help-seeking for mental health problems. *AeJAMH (Australian e-Journal for the Advancement of Mental Health)* 2005;4(3):No Pagination Specified.
23. Saraceno B, van Ommeren M, Batniji R, et al. Barriers to improvement of mental health services in low-income and middle-income countries. *Lancet* 2007;370(9593):1164-74. doi: 10.1016/s0140-6736(07)61263-x [published Online First: 2007/09/07]
24. Aguirre Velasco A, Cruz ISS, Billings J, et al. What are the barriers, facilitators and interventions targeting help-seeking behaviours for common mental health problems in adolescents? A systematic review. *BMC Psychiatry* 2020;20(1):293. doi: 10.1186/s12888-020-02659-0
25. Jorm AF. Mental health literacy: empowering the community to take action for better mental health. *Am Psychol* 2012;67(3):231-43. doi: 10.1037/a0025957 [published Online First: 2011/11/02]
26. WHO. Adolescent Mental Health Geneva: WHO 2020 [Available from: https://www.who.int/news-room/fact-sheets/detail/adolescent-mental-health accessed 1 July 2019 2019.
27. World Health O. Quality assessment guidebook: a guide to assessing health services for adolescent clients. Geneva: World Health Organization, 2009.
28. Ambresin A-E, Bennett K, Patton GC, et al. Assessment of youth-friendly health care: a systematic review of indicators drawn from young people's perspectives. *The Journal of adolescent health : official publication of the Society for Adolescent Medicine* 2013;52(6):670-81. doi: 10.1016/j.jadohealth.2012.12.014
29. WHO O, WB. Delivering Quality Health Services: A Global Imperative for Universal Health Coverage. Geneva: World Health Organization 2018.
30. Chisholm D, Flisher AJ, Lund C, et al. Scale up services for mental disorders: a call for action. *Lancet* 2007;370(9594):1241-52. doi: 10.1016/s0140-6736(07)61242-2 [published Online First: 2007/09/07]
31. Barry MM, Clarke AM, Jenkins R, et al. A systematic review of the effectiveness of mental health promotion interventions for young people in low and middle income countries. *BMC Public Health* 2013;13:835. doi: 10.1186/1471-2458-13-835 [published Online First: 2013/09/13]

For peer review only

32. Sayal K, Amarasinghe M, Robotham S, et al. Quality standards for child and adolescent mental health in primary care. *BMC Fam Pract* 2012;13:51-51. doi: 10.1186/1471-2296-13-51
33. McDonald CC, Deatrick JA, Kassam-Adams N, et al. Community violence exposure and positive youth development in urban youth. *J Community Health* 2011;36(6):925-32. doi: 10.1007/s10900-011-9391-5
34. Slopen N, Fitzmaurice GM, Williams DR, et al. Common patterns of violence experiences and depression and anxiety among adolescents. *Soc Psychiatry Psychiatr Epidemiol* 2012;47(10):1591-605. doi: 10.1007/s00127-011-0466-5 [published Online First: 2012/01/14]
35. Finkelhor D, Ormrod RK, Turner HA. Poly-victimization: a neglected component in child victimization. *Child Abuse Negl* 2007;31(1):7-26. doi: 10.1016/j.chiabu.2006.06.008 [published Online First: 2007/01/17]
36. Cooley-Quille M, Boyd RC, Frantz E, et al. Emotional and behavioral impact of exposure to community violence in inner-city adolescents. *J Clin Child Psychol* 2001;30(2):199-206. doi: 10.1207/s15374424jccp3002_7 [published Online First: 2001/06/08]
37. Buka SL, Stichick TL, Birdthistle I, et al. Youth exposure to violence: prevalence, risks, and consequences. *Am J Orthopsychiatry* 2001;71(3):298-310. doi: 10.1037/0002-9432.71.3.298 [published Online First: 2001/08/10]
38. Hammack PL, Richards MH, Luo Z, et al. Social support factors as moderators of community violence exposure among inner-city African American young adolescents. *J Clin Child Adolesc Psychol* 2004;33(3):450-62. doi: 10.1207/s15374424jccp3303_3 [published Online First: 2004/07/24]
39. Fitzpatrick S, Saraiya T, Lopez-Castro T, et al. The impact of trauma characteristics on post-traumatic stress disorder and substance use disorder outcomes across integrated and substance use treatments. *Journal of Substance Abuse Treatment* 2020;113:107976. doi: https://doi.org/10.1016/j.jsat.2020.01.012
40. McGorry PD, Hartmann JA, Spooner R, et al. Beyond the "at risk mental state" concept: transitioning to transdiagnostic psychiatry. *World Psychiatry* 2018;17(2):133-42. doi: 10.1002/wps.20514
41. WHO. mhGAP: Mental Health Gap Action Programme: scaling up care for mental, neurological and substance use disorders Geneva: World Health Organization, 2008.
42. Nair M, Baltag V, Bose K, et al. Improving the Quality of Health Care Services for Adolescents, Globally: A Standards-Driven Approach. *The Journal of adolescent health : official publication of the Society for Adolescent Medicine* 2015;57(3):288-98. doi: 10.1016/j.jadohealth.2015.05.011
43. Moher D, Liberati A, Tetzlaff J, et al. Preferred reporting items for systematic reviews and meta-analyses: the PRISMA statement. *BMJ* 2009;339:b2535. doi: 10.1136/bmj.b2535
44. Endnote X9 [program]. London: Clarivate Analytics, 2019.
45. NIH. Quality assessment tool for observational cohort and cross-sectional studies Washington, DC: National Institutes of Health; 2014 [Available from: https://www.nhlbi.nih.gov/health-topics/study-quality-assessment-tools accessed 9 November 2019.
46. Davidson TM, Bunnell BE, Saunders BE, et al. Pilot evaluation of a tablet-based application to improve quality of care in child mental health treatment. *Behavior Therapy* 2018:No Pagination Specified.

For peer review only - http://bmjopen.bmj.com/site/about/guidelines.xhtml

47. Jager M, Reijneveld SA, Almansa J, et al. Less reduction of psychosocial problems among adolescents with unmet communication needs. *European Child & Adolescent Psychiatry* 2017;26(4):403-12. doi: 10.1007/s00787-016-0901-y
48. Ougrin D, Corrigall R, Poole J, et al. Comparison of effectiveness and cost-effectiveness of an intensive community supported discharge service versus treatment as usual for adolescents with psychiatric emergencies: A randomised controlled trial. *The Lancet Psychiatry* 2018;5(6):477-85.
49. Dion J, Kennedy A, Cloutier P, et al. Evaluating crisis intervention services for youth within an emergency department: A view from within. *Child Care in Practice* 2010;16(3):241-56.
50. Spenser HR, Gillies A, Maysenhoelder H. The CHAT project: Paediatricians and mental health clinicians: Working together for the sake of the children. *Journal of the Canadian Academy of Child and Adolescent Psychiatry* 2009;18(2):110-16.
51. Ayton A, Meads G. The implementation of the care programme approach in the west midlands CAMHS: The impact of organisational diversity on patient safety - an extended audit. *International Journal of Clinical Leadership* 2013;17(4):185-200.
52. Aupont O, Doerfler L, Connor DF, et al. A collaborative care model to improve access to pediatric mental health services. *Administration and policy in mental health* 2013;40(4):264-73. doi: 10.1007/s10488-012-0413-0 [published Online First: 2012/04/25]
53. Simmons MB, Elmes A, McKenzie JE, et al. Right choice, right time: Evaluation of an online decision aid for youth depression. *Health Expect* 2017;20(4):714-23. doi: 10.1111/hex.12510 [published Online First: 2016/10/18]
54. Irvine C. Reviewing Mental Health Services and Support for Children and Young People in Northern Ireland: A Rights-Based Approach. *Child Care in Practice* 2020:1-11. doi: 10.1080/13575279.2020.1809347
55. Jager M, Reijneveld SA, Metselaar J, et al. Discrepancies between adolescents' attributed relevance and experiences regarding communication are associated with poorer client participation and learning processes in psychosocial care. *Patient education and counseling* 2014;97(3):332-8. doi: 10.1016/j.pec.2014.08.020 [published Online First: 2014/09/17]
56. Stevens J, Klima J, Chisolm D, et al. A trial of telephone services to increase adolescent utilization of health care for psychosocial problems. *J Adolesc Health* 2009;45(6):564-70. doi: 10.1016/j.jadohealth.2009.04.003 [published Online First: 2009/11/26]
57. Anderson RE, Spence SH, Donovan CL, et al. Working alliance in online cognitive behavior therapy for anxiety disorders in youth: comparison with clinic delivery and its role in predicting outcome. *J Med Internet Res* 2012;14(3):e88. doi: 10.2196/jmir.1848 [published Online First: 2012/07/14]
58. Kapp C, Perlini T, Jeanneret T, et al. Identifying the determinants of perceived quality in outpatient child and adolescent mental health services from the perspectives of parents and patients. *European Child & Adolescent Psychiatry* 2017;26(10):1269-77. doi: 10.1007/s00787-017-0985-z
59. Cairns AJ, Kavanagh DJ, Dark F, et al. Goal setting improves retention in youth mental health: a cross-sectional analysis. *Child & Adolescent Psychiatry & Mental Health [Electronic Resource]* 2019;13:31.
60. Mora Ringle VA, Hickey JS, Jensen-Doss A. Patterns and predictors of compliance with utilization management guidelines supporting a state policy to improve the quality of

For peer review only - http://bmjopen.bmj.com/site/about/guidelines.xhtml

youth mental health services. *Child Youth Serv Rev* 2019;96:194-203. doi: 10.1016/j.childyouth.2018.11.035 [published Online First: 11/16]
61. Sattler AF, Leffler JM, Harrison NL, et al. The quality of assessments for childhood psychopathology within a regional medical center. *Psychological Services* 2019;16(4):596-604.
62. Sattler AF, Ale CM, Nguyen K, et al. Use of evidence-based assessments for childhood anxiety disorders within a regional medical system. *Psychol Serv* 2016;13(4):411-18. doi: 10.1037/ser0000091 [published Online First: 2016/11/01]
63. Higa-McMillan C, Kotte A, Jackson D, et al. Overlapping and Non-overlapping Practices in Usual and Evidence-Based Care for Youth Anxiety. *The Journal of Behavioral Health Services & Research* 2017;44(4):684-94. doi: 10.1007/s11414-016-9502-2
64. Rukundo GZ, Nalugya J, Otim P, et al. A Collaborative Approach to the Development of Multi-Disciplinary Teams and Services for Child and Adolescent Mental Health in Uganda. *Front Psychiatry* 2020;11:579417. doi: 10.3389/fpsyt.2020.579417 [published Online First: 2020/11/27]
65. Bardach NS, Doupnik SK, Rodean J, et al. ED Visits and Readmissions After Follow-up for Mental Health Hospitalization. *Pediatrics* 2020;145(6):e20192872. doi: 10.1542/peds.2019-2872
66. Mukolo A, Heflinger CA, Wallston KA. The stigma of childhood mental disorders: a conceptual framework. *J Am Acad Child Adolesc Psychiatry* 2010;49(2):92-198. doi: 10.1097/00004583-201002000-00003
67. Gulliver A, Griffiths KM, Christensen H. Perceived barriers and facilitators to mental health help-seeking in young people: a systematic review. *BMC Psychiatry* 2010;10(1):113. doi: 10.1186/1471-244X-10-113
68. Radez J, Reardon T, Creswell C, et al. Why do children and adolescents (not) seek and access professional help for their mental health problems? A systematic review of quantitative and qualitative studies. *European Child & Adolescent Psychiatry* 2020 doi: 10.1007/s00787-019-01469-4
69. Commission CQ. Review of children and young people's mental health services: Phase One supporting documentation: Summary of recent policy and literature. London: Care Quality Commission, 2017.
70. Corrigan P. How stigma interferes with mental health care. *Am Psychol* 2004;59(7):614-25. doi: 10.1037/0003-066x.59.7.614 [published Online First: 2004/10/20]
71. JD L. Mental illness-related structural stigma: the downward spiral of systemic exclusion. Alberta, Canada: Mental Health Commission of Canada, 2013.
72. DuPont-Reyes MJ, Villatoro AP, Phelan JC, et al. Adolescent views of mental illness stigma: An intersectional lens. *Am J Orthopsychiatry* 2020;90(2):201-11. doi: 10.1037/ort0000425 [published Online First: 2019/08/06]
73. Patton G, Temmerman M. Evidence and Evidence Gaps in Adolescent Health. *The Journal of adolescent health : official publication of the Society for Adolescent Medicine* 2016;59(4S):S1-S3. doi: 10.1016/j.jadohealth.2016.08.001
74. Das JK, Salam RA, Lassi ZS, et al. Interventions for Adolescent Mental Health: An Overview of Systematic Reviews. *The Journal of adolescent health : official publication of the Society for Adolescent Medicine* 2016;59(4S):S49-S60. doi: 10.1016/j.jadohealth.2016.06.020
75. Juengsiragulwit D. Opportunities and obstacles in child and adolescent mental health services in low- and middle-income countries: a review of the literature. *WHO South*

For peer review only - http://bmjopen.bmj.com/site/about/guidelines.xhtml

East Asia J Public Health 2015;4(2):110-22. doi: 10.4103/2224-3151.206680 [published Online First: 2015/07/01]
76. Sawyer SM, Ambresin AE, Bennett KE, et al. A measurement framework for quality health care for adolescents in hospital. *J Adolesc Health* 2014;55(4):484-90. doi: 10.1016/j.jadohealth.2014.01.023 [published Online First: 2014/04/05]
77. NHS. You're Welcome Quality Criteria: Making health services young people friendly. . London: NHS, 2007.
78. O'Connor AM, Légaré F, Stacey D. Risk communication in practice: the contribution of decision aids. *Bmj* 2003;327(7417):736-40. doi: 10.1136/bmj.327.7417.736 [published Online First: 2003/09/27]
79. Hawley KM, Garland AF. Working Alliance in Adolescent Outpatient Therapy: Youth, Parent and Therapist Reports and Associations with Therapy Outcomes. *Child & Youth Care Forum* 2008;37(2):59-74. doi: 10.1007/s10566-008-9050-x
80. Marker CD, Comer JS, Abramova V, et al. The reciprocal relationship between alliance and symptom improvement across the treatment of childhood anxiety. *J Clin Child Adolesc Psychol* 2013;42(1):22-33. doi: 10.1080/15374416.2012.723261 [published Online First: 2012/09/27]
81. Anstiss D, Davies A. 'Reach Out, Rise Up': The efficacy of text messaging in an intervention package for anxiety and depression severity in young people. *Child Youth Serv Rev* 2015;58:99-103. doi: https://doi.org/10.1016/j.childyouth.2015.09.011
82. Gondek D, Edbrooke-Childs J, Velikonja T, et al. Facilitators and Barriers to Person-centred Care in Child and Young People Mental Health Services: A Systematic Review. *Clin Psychol Psychother* 2017;24(4):870-86. doi: 10.1002/cpp.2052 [published Online First: 2016/12/03]
83. Weithorn LA, Campbell SB. The Competency of Children and Adolescents to Make Informed Treatment Decisions. *Child Development* 1982;53(6):1589-98. doi: 10.2307/1130087
84. Langer DA, Jensen-Doss A. Shared Decision-Making in Youth Mental Health Care: Using the Evidence to Plan Treatments Collaboratively. *J Clin Child Adolesc Psychol* 2018;47(5):821-31. doi: 10.1080/15374416.2016.1247358 [published Online First: 2016/12/03]
85. Psychiatry FoCaAPaFoGA. Good mental health services for young people. London: Royal College of Psychiatrists, 2017.
86. C L. Working at the CAMHS/Adult Interface: Good practice guidance for the provision of psychiatric services to adolescents/young adults. London: Royal College of Psychiatrists 2008.
87. Birchwood M, Singh SP. Mental health services for young people: matching the service to the need. *Br J Psychiatry Suppl* 2013;54:s1-2. doi: 10.1192/bjp.bp.112.119149 [published Online First: 2013/01/11]
88. O'Brien D, Harvey K, Howse J, et al. Barriers to managing child and adolescent mental health problems: a systematic review of primary care practitioners' perceptions. *Br J Gen Pract* 2016;66(651):e693-707. doi: 10.3399/bjgp16X687061 [published Online First: 2016/09/14]
89. Kutcher S, Davidson S, Manion I. Child and youth mental health: Integrated health care using contemporary competency-based teams. *Paediatr Child Health* 2009;14(5):315-18. doi: 10.1093/pch/14.5.315

For peer review only - http://bmjopen.bmj.com/site/about/guidelines.xhtml

90. Loades ME, Chatburn E, Higson-Sweeney N, et al. Rapid Systematic Review: The Impact of Social Isolation and Loneliness on the Mental Health of Children and Adolescents in the Context of COVID-19. *Journal of the American Academy of Child & Adolescent Psychiatry* 2020;59(11):1218-39.e3. doi: https://doi.org/10.1016/j.jaac.2020.05.009
91. Thompson EC, Thomas SA, Burke TA, et al. Suicidal thoughts and behaviors in psychiatrically hospitalized adolescents pre- and post- COVID-19: A historical chart review and examination of contextual correlates. *J Affect Disord Rep* 2021;4:100100-00. doi: 10.1016/j.jadr.2021.100100 [published Online First: 01/29]
92. O'Sullivan K, Clark S, McGrane A, et al. A Qualitative Study of Child and Adolescent Mental Health during the COVID-19 Pandemic in Ireland. *Int J Environ Res Public Health* 2021;18(3) doi: 10.3390/ijerph18031062 [published Online First: 2021/01/29]
93. World Health O. The impact of COVID-19 on mental, neurological and substance use services: results of a rapid assessment. Geneva: World Health Organization 2020:36 p.
94. de Figueiredo CS, Sandre PC, Portugal LCL, et al. COVID-19 pandemic impact on children and adolescents' mental health: Biological, environmental, and social factors. *Progress in Neuro-Psychopharmacology and Biological Psychiatry* 2021;106:110171. doi: https://doi.org/10.1016/j.pnpbp.2020.110171
95. Kilbourne AM, Beck K, Spaeth-Rublee B, et al. Measuring and improving the quality of mental health care: a global perspective. *World Psychiatry* 2018;17(1):30-38. doi: 10.1002/wps.20482
96. OECD. Making mental health count. Focus on health. . Paris: OECD, 2014.
97. WHO. Integrating mental health into primary care: A global perspective. Geneva: World Health Organization, 2008.
98. Patel V, Saxena S, Lund C, et al. The Lancet Commission on global mental health and sustainable development. *The Lancet* 2018;392(10157):1553-98. doi: 10.1016/S0140-6736(18)31612-X
99. Leslie HH, Hirschhorn LR, Marchant T, et al. Health systems thinking: A new generation of research to improve healthcare quality. *PLoS Med* 2018;15(10):e1002682-e82. doi: 10.1371/journal.pmed.1002682
100. Sawyer SM, Azzopardi PS, Wickremarathne D, et al. The age of adolescence. *The Lancet Child & Adolescent Health* 2018;2(3):223-28. doi: https://doi.org/10.1016/S2352-4642(18)30022-1
101. Olfson M, He JP, Merikangas KR. Psychotropic medication treatment of adolescents: results from the National Comorbidity Survey-Adolescent Supplement. *J Am Acad Child Adolesc Psychiatry* 2013;52(4):378-88. doi: 10.1016/j.jaac.2012.12.006 [published Online First: 2013/04/16]
102. Tsai CY, Yang HC, Islam M, et al. Psychotropic medications prescribing trends in adolescents: A nationwide population-based study in Taiwan. *Int J Qual Health Care* 2017;29(6):861-66. doi: 10.1093/intqhc/mzx123 [published Online First: 2017/10/17]
103. B L, C D, D M, et al. Principles in using psychotropic medication in children and adolescents. In: In Rey JM MA, ed. IACAPAP e-Textbook of Child and Adolescent Mental Health. Geneva: International Association for Child and Adolescent Psychiatry and Allied Professions 2019 2019.
104. Langarizadeh M, Tabatabaei MS, Tavakol K, et al. Telemental Health Care, an Effective Alternative to Conventional Mental Care: a Systematic Review. *Acta Inform Med* 2017;25(4):240-46. doi: 10.5455/aim.2017.25.240-246

For peer review only

For peer review only - http://bmjopen.bmj.com/site/about/guidelines.xhtml

105. Galagali PM, Brooks MJ. Psychological care in low-resource settings for adolescents. *Clin Child Psychol Psychiatry* 2020;25(3):698-711. doi: 10.1177/1359104520929741 [published Online First: 2020/06/23]
106. Wood SM, White K, Peebles R, et al. Outcomes of a Rapid Adolescent Telehealth Scale-Up During the COVID-19 Pandemic. *J Adolesc Health* 2020;67(2):172-78. doi: 10.1016/j.jadohealth.2020.05.025 [published Online First: 2020/07/03]
107. Skinner H, Biscope S, Poland B, et al. How Adolescents Use Technology for Health Information: Implications for Health Professionals from Focus Group Studies. *J Med Internet Res* 2003;5(4):e32. doi: 10.2196/jmir.5.4.e32 [published Online First: 18.12.2003]
108. Garrett CC, Hocking J, Chen MY, et al. Young people's views on the potential use of telemedicine consultations for sexual health: results of a national survey. *BMC Infectious Diseases* 2011;11(1):285. doi: 10.1186/1471-2334-11-285

Table 1 References

Knaak S, Patten S, Ungar T. Mental illness stigma as a quality-of-care problem. *Lancet Psychiatry* 2015;2(10):863-4.

Saraceno B, van Ommeren M, Batniji R, et al. Barriers to improvement of mental health services in low-income and middle-income countries. *Lancet* 2007;370(9593):1164-74.

McPherson A. Adolescents in primary care. *BMJ* 2005;330(7489):465-7.

Svirydzenka N, Ronzoni P, Dogra N. Meaning and barriers to quality care service provision in Child and Adolescent Mental Health Services: Qualitative study of stakeholder perspectives. *BMC Health Serv Res* 2017;17(1):151.

Box 1 References

Kruk ME, Gage AD, Arsenault C, et al. High-quality health systems in the Sustainable Development Goals era: time for a revolution. *Lancet Glob Health* 2018;6(11):e1196-e252.

WHO, OECD, WB. Delivering Quality Health Services: A Global Imperative for Universal Health Coverage. Geneva: World Health Organization 2018.

IOM. Crossing the Quality Chasm: A New Health System for the 21st Century. Washington(DC): National Academies Press(US).

Table 3 References

Davidson TM, Bunnell BE, Saunders BE, et al. Pilot evaluation of a tablet-based application to improve quality of care in child mental health treatment. Behavior Therapy 2018:No Pagination Specified.

Jager M, Reijneveld SA, Almansa J, et al. Less reduction of psychosocial problems among adolescents with unmet communication needs. European Child & Adolescent Psychiatry 2017;26(4):403-12. doi: 10.1007/s00787-016-0901-y

Ougrin D, Corrigall R, Poole J, et al. Comparison of effectiveness and cost-effectiveness of an intensive community supported discharge service versus treatment as usual for adolescents with psychiatric emergencies: A randomised controlled trial. The Lancet Psychiatry 2018;5(6):477-85.

For peer review only - http://bmjopen.bmj.com/site/about/guidelines.xhtml

Dion J, Kennedy A, Cloutier P, et al. Evaluating crisis intervention services for youth within an emergency department: A view from within. Child Care in Practice 2010;16(3):241-56.

Spenser HR, Gillies A, Maysenhoelder H. The CHAT project: Paediatricians and mental health clinicians: Working together for the sake of the children. Journal of the Canadian Academy of Child and Adolescent Psychiatry 2009;18(2):110-16.

Ayton A, Meads G. The implementation of the care programme approach in the west midlands CAMHS: The impact of organisational diversity on patient safety - an extended audit. International Journal of Clinical Leadership 2013;17(4):185-200.

Aupont O, Doerfler L, Connor DF, et al. A collaborative care model to improve access to pediatric mental health services. Administration and policy in mental health 2013;40(4):264-73. doi: 10.1007/s10488-012-0413-0 [published Online First: 2012/04/25]

Simmons MB, Elmes A, McKenzie JE, et al. Right choice, right time: Evaluation of an online decision aid for youth depression. Health Expect 2017;20(4):714-23. doi: 10.1111/hex.12510 [published Online First: 2016/10/18]

Jager M, Reijneveld SA, Metselaar J, et al. Discrepancies between adolescents' attributed relevance and experiences regarding communication are associated with poorer client participation and learning processes in psychosocial care. Patient education and counseling 2014;97(3):332-8. doi: 10.1016/j.pec.2014.08.020 [published Online First: 2014/09/17]

Stevens J, Klima J, Chisolm D, et al. A trial of telephone services to increase adolescent utilization of health care for psychosocial problems. J Adolesc Health 2009;45(6):564-70. doi: 10.1016/j.jadohealth.2009.04.003 [published Online First: 2009/11/26]

Anderson RE, Spence SH, Donovan CL, et al. Working alliance in online cognitive behavior therapy for anxiety disorders in youth: comparison with clinic delivery and its role in predicting outcome. J Med Internet Res 2012;14(3):e88. doi: 10.2196/jmir.1848 [published Online First: 2012/07/14]

Kapp C, Perlini T, Jeanneret T, et al. Identifying the determinants of perceived quality in outpatient child and adolescent mental health services from the perspectives of parents and patients. European Child & Adolescent Psychiatry 2017;26(10):1269-77. doi: 10.1007/s00787-017-0985-z

Cairns AJ, Kavanagh DJ, Dark F, et al. Goal setting improves retention in youth mental health: a cross-sectional analysis. Child & Adolescent Psychiatry & Mental Health [Electronic Resource] 2019;13:31.

Mora Ringle VA, Hickey JS, Jensen-Doss A. Patterns and predictors of compliance with utilization management guidelines supporting a state policy to improve the quality of youth mental health services. Child Youth Serv Rev 2019;96:194-203. doi: 10.1016/j.childyouth.2018.11.035 [published Online First: 11/16]

Sattler AF, Leffler JM, Harrison NL, et al. The quality of assessments for childhood psychopathology within a regional medical center. Psychological Services 2019;16(4):596-604.

Sattler AF, Ale CM, Nguyen K, et al. Use of evidence-based assessments for childhood anxiety disorders within a regional medical system. Psychol Serv 2016;13(4):411-18. doi: 10.1037/ser0000091 [published Online First: 2016/11/01]

For peer review only - http://bmjopen.bmj.com/site/about/guidelines.xhtml

Higa-McMillan C, Kotte A, Jackson D, et al. Overlapping and Non-overlapping Practices in Usual and Evidence-Based Care for Youth Anxiety. The Journal of Behavioral Health Services & Research 2017;44(4):684-94. doi: 10.1007/s11414-016-9502-2.

Bardach NS, Doupnik SK, Rodean J, et al. ED Visits and Readmissions After Follow-up for Mental Health Hospitalization. Pediatrics 2020;145(6):e20192872. doi: 10.1542/peds.2019-2872.

Irvine C. Reviewing Mental Health Services and Support for Children and Young People in Northern Ireland: A Rights-Based Approach. Child Care in Practice 2020:1-11. doi: 10.1080/13575279.2020.1809347.

Rukundo GZ, Nalugya J, Otim P, et al. A Collaborative Approach to the Development of Multi-Disciplinary Teams and Services for Child and Adolescent Mental Health in Uganda. Front Psychiatry 2020;11:579417. doi: 10.3389/fpsyt.2020.579417.

Table 4 References

Sawyer SM, Ambresin AE, Bennett KE, et al. A measurement framework for quality health care for adolescents in hospital. J Adolesc Health 2014;55(4):484-90. doi: 10.1016/j.jadohealth.2014.01.023 [published Online First: 2014/04/05].

Ambresin A-E, Bennett K, Patton GC, et al. Assessment of youth-friendly health care: a systematic review of indicators drawn from young people's perspectives. The Journal of adolescent health : official publication of the Society for Adolescent Medicine 2013;52(6):670-81. doi: 10.1016/j.jadohealth.2012.12.014.

NHS. You're Welcome Quality Criteria: Making health services young people friendly. London: NHS, 2007.

Sayal K, Amarasinghe M, Robotham S, et al. Quality standards for child and adolescent mental health in primary care. *BMC Fam Pract* 2012;13:51-51. doi: 10.1186/1471-2296-13-51

For peer review only

Figure 1. Flow diagram of studies

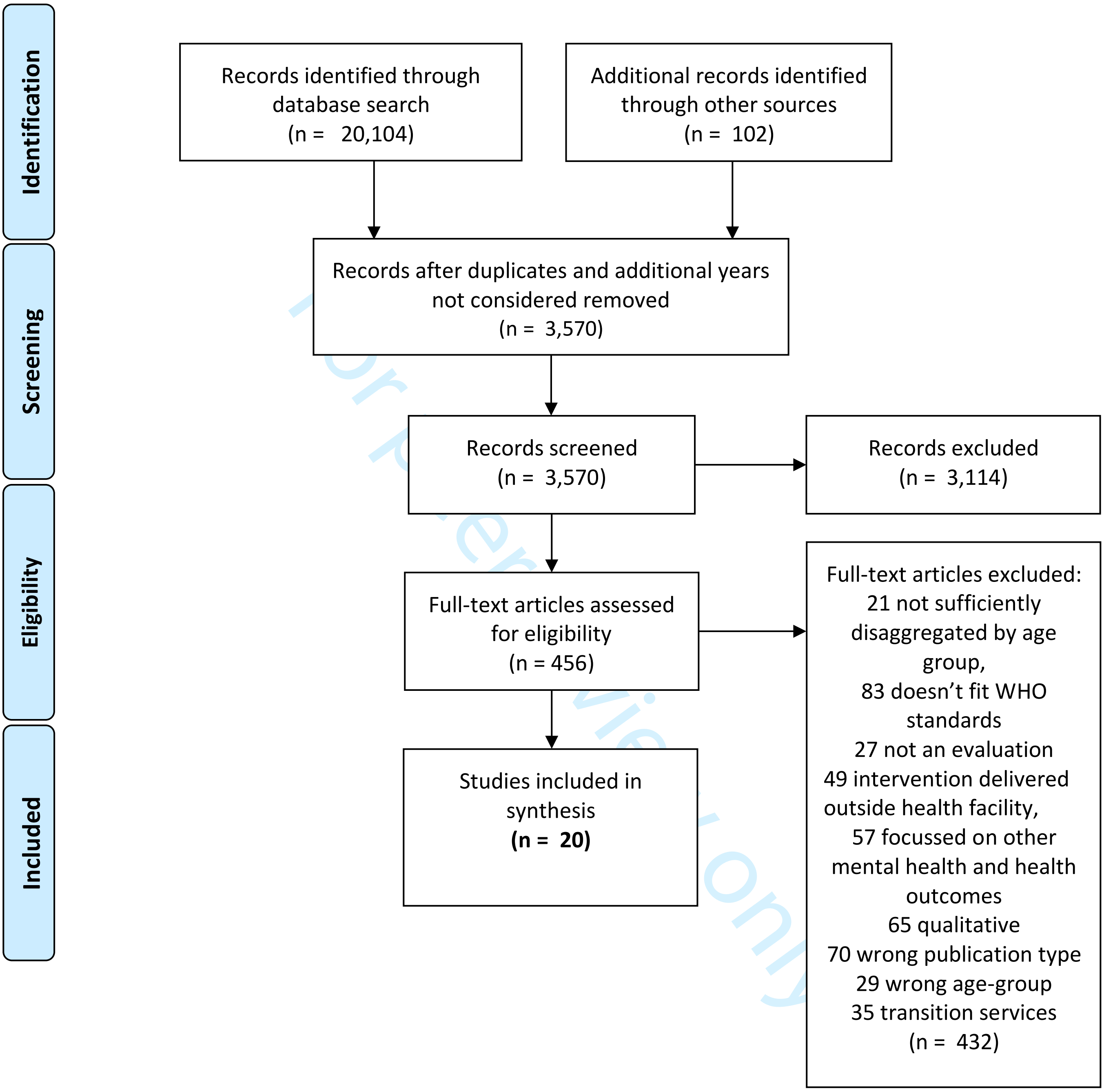

For peer review only - http://bmjopen.bmj.com/site/about/guidelines.xhtml

For peer review only

For peer review only - http://bmjopen.bmj.com/site/about/guidelines.xhtml

PROSPERO
International prospective register of systematic reviews

Evaluating quality in adolescent mental health service delivery: a systematic review

Meaghen Quinlan-Davidson, Kathryn Roberts, Delan Devakumar, Susan Sawyer, Ligia Kiss

Supplemental Material 1. PROSPERO Registration

Citation

Meaghen Quinlan-Davidson, Kathryn Roberts, Delan Devakumar, Susan Sawyer, Ligia Kiss. Evaluating quality in adolescent mental health service delivery: a systematic review. PROSPERO 2020 CRD42020161318 Available from: https://www.crd.york.ac.uk/prospero/display_record.php?ID=CRD42020161318

Review question

What is the global evidence on the aspects of quality of adolescent mental health services (based on the WHO Global standards for quality healthcare services for adolescents) including adolescent mental health literacy, provider competencies, and appropriate packages of services for adolescents with depressive, anxiety, and post-traumatic stress disorders?

Searches

-2008-2018

-Sources include (but are not limited to) PubMed, PscyINFO, MEDLINE, EMBASE, LILACS

-Reference checking of articles generated from electronic database

-Hand searching of references of identified manuscripts

-No restriction on language

Types of study to be included

Evaluations or assessments of RCTs, case-control, cohort, cross-sectional, longitudinal, mixed-methods (quantitative data only).

Condition or domain being studied

Quality in adolescent mental health services.

Participants/population

Study participants include individual patients (10-19 years of age) using mental healthcare services, or adolescents in the community asked where they would seek services for depression, anxiety, or PTSD for themselves; and providers of mental health services, including general practitioners, psychologists, and psychiatrists.

Where study participants include children and adolescents together or adolescents and youth together, the study will be included when the results are sufficiently disaggregated that they can be interpreted for at least part of the 10-19 year old age span.

Intervention(s), exposure(s)

Studies that evaluate or assess aspects of quality measures (as conforms to the WHO Global standards for quality healthcare services for adolescents) applied to existing adolescent mental health services, who have used, are currently using, or could potentially seek to use mental health services, or are exposed to interventions or strategies within established mental health services, regardless of health facility level.

Comparator(s)/control

For intervention studies only: Intervention vs. control arm (randomized trial), quasi-experimental (open trial with pre- and post-intervention design or matched control group).

Main outcome(s)

For peer review only

Studies that include quality, as conforms to the WHO WHO Global standards for quality healthcare services for adolescents, including:

-Adolescents' health literacy (The health facility implements systems to ensure that adolescents are knowledgeable about their own health, and they know where and when to obtain health services);

-Appropriate package of services (The health facility provides a package of information, counselling, diagnostic, treatment and care services that fulfils the needs of all adolescents. Services are provided in the facility and through referral linkages and outreach);

-Providers' competencies (Health care providers demonstrate the technical competence required to provide effective health services to adolescents. Both health-care providers and support staff respect, protect and fulfil adolescents' rights to information, privacy, confidentiality, non-discrimination, non-judgmental attitude and respect)

* Measures of effect

Not applicable.

Additional outcome(s)

None.

* Measures of effect

Not applicable.

Data extraction (selection and coding)

All results from the above mentioned search strategy will be exported to Endnote, an electronic reference manager; duplicates will be removed. Independently, two authors (MQD and KR) will screen the titles and abstracts of all studies identified through the search strategy that potentially meet the inclusion criteria mentioned above. The full text of these potentially eligible studies will be retrieved and independently assessed for eligibility by the two team members. The main data to be extracted will include study design, publication type, area of quality, reason (if any) for exclusion), type of service and mental health issue, purpose of study, conceptualisation of quality or quality in relation to or effect of quality on, intervention/measured used, sample, outcomes, and location. Disagreement over the eligibility of particular studies will be resolved through discussion. Discussion will be held to resolve any discrepancies, with additional authors when needed.

Risk of bias (quality) assessment

NIH Quality Assessment Tool

Strategy for data synthesis

Random effects meta-analyses will be conducted to determine intervention effects, if sufficient data exist and levels of heterogeneity are appropriate. A minimum of three studies will be required per meta-analysis. If data cannot be pooled between intervention studies, findings will be reported in narrative synthesis. Results will be organised and interpreted according to the WHO Global Standards for Quality Healthcare Services for Adolescents. This will include the number of studies that address each element of quality, as well as a summary of the results of each study. Also, results on the conceptualisation of quality or how quality was mentioned in the article will be summarised, geographic location of the studies, and a search and selection strategy will be presented. A table, summarising the characteristics of the study, will be included that provides information on the service, intervention description, study/evaluation design, target population, element of quality addressed, results, and quality assessment will be included.

Analysis of subgroups or subsets

Not applicable.

Contact details for further information

Meaghen Quinlan-Davidson
meaghen.quinlan-davidson.17@ucl.ac.uk

For peer review only

Organisational affiliation of the review
Institute for Global Health, University College London

Review team members and their organisational affiliations
Miss Meaghen Quinlan-Davidson. Institute for Global Health, University College London
Miss Kathryn Roberts. Institute for Global Health, University College London
Dr Delan Devakumar. Institute for Global Health, University College London
Dr Susan Sawyer. Centre for Adolescent Health, Royal Children's Hospital, Melbourne, Australia
Dr Ligia Kiss. Institute for Global Health, University College London

Type and method of review
Service delivery, Systematic review

Anticipated or actual start date
12 November 2019

Anticipated completion date
31 January 2020

Funding sources/sponsors
None.

Conflicts of interest

Language
English

Country
England

Stage of review
Review Ongoing

Subject index terms status
Subject indexing assigned by CRD

Subject index terms
Adolescent; Adolescent Health Services; Delivery of Health Care; Humans; Mental Health Services

Date of registration in PROSPERO
27 January 2020

Date of first submission
05 December 2019

Details of any existing review of the same topic by the same authors

Stage of review at time of this submission

For peer review only

Stage	Started	Completed
Preliminary searches	Yes	No
Piloting of the study selection process	Yes	No
Formal screening of search results against eligibility criteria	Yes	No
Data extraction	No	No
Risk of bias (quality) assessment	No	No
Data analysis	No	No

The record owner confirms that the information they have supplied for this submission is accurate and complete and they understand that deliberate provision of inaccurate information or omission of data may be construed as scientific misconduct.

The record owner confirms that they will update the status of the review when it is completed and will add publication details in due course.

Versions

27 January 2020

PROSPERO
This information has been provided by the named contact for this review. CRD has accepted this information in good faith and registered the review in PROSPERO. The registrant confirms that the information supplied for this submission is accurate and complete. CRD bears no responsibility or liability for the content of this registration record, any associated files or external websites.

For peer review only

Supplemental Material 2. Process and Output Criteria used for WHO Quality Standards to Select Studies[1]

For *adolescent mental health literacy*, this involved identifying and assessing studies using the following criteria:

	**Adolescent mental health literacy: Selection criteria for studies**
**PROCESS**	the provision of mental health education and counselling to adolescents by healthcare providers and services that are age and developmentally appropriate
	the provision of information about the availability of mental health, social, and other services to adolescents by healthcare providers and services
	outreach activities by the healthcare facility to promote mental health and increase the use of adolescent mental health services
**OUTPUT**	adolescents increased knowledge about mental health and awareness about mental health services

For *appropriate package of services*, we identified and assessed studies according to the following:

	**Appropriate package of services: Selection criteria for studies**
**PROCESS**	within healthcare services, the provision of a package of mental health information, counselling, diagnostic, treatment and care services to adolescents that meets their needs
	referral to appropriate services
**OUTPUT**	adolescents receive a package of services that meets their needs, within healthcare or referral services

For *provider competencies*, this involved identifying and assessing studies using the following criteria:

	**Provider competencies: Selection criteria for studies**
**PROCESS**	healthcare providers adhere to and implement adolescent mental health evidence-based guidelines and protocols
	healthcare providers provide friendly services to adolescents and respect their rights to information, privacy, confidentiality, non-discrimination, non-judgement attitude, and respectful care
**OUTPUT**	adolescents receive effective mental health services
	adolescents obtain mental health services that are friendly, supportive, respectful, non-discriminatory and non-judgemental manner, and know their rights in healthcare
	adolescents receive from the healthcare facility accurate, age-appropriate and clear information to facilitate informed choice

References

1. WHO. Global standards for quality health care services for adolescents. Geneva: World Health Organization, 2015.

For peer review only

Supplemental Material 3. PRISMA 2009 Checklist

Section/topic	#	Checklist item	Reported on page #
**TITLE**			
Title	1	Identify the report as a systematic review, meta-analysis, or both.	1
**ABSTRACT**			
Structured summary	2	Provide a structured summary including, as applicable: background; objectives; data sources; study eligibility criteria, participants, and interventions; study appraisal and synthesis methods; results; limitations; conclusions and implications of key findings; systematic review registration number.	2
**INTRODUCTION**			
Rationale	3	Describe the rationale for the review in the context of what is already known.	4-8
Objectives	4	Provide an explicit statement of questions being addressed with reference to participants, interventions, comparisons, outcomes, and study design (PICOS).	8
**METHODS**			
Protocol and registration	5	Indicate if a review protocol exists, if and where it can be accessed (e.g., Web address), and, if available, provide registration information including registration number.	8
Eligibility criteria	6	Specify study characteristics (e.g., PICOS, length of follow-up) and report characteristics (e.g., years considered, language, publication status) used as criteria for eligibility, giving rationale.	8-9
Information sources	7	Describe all information sources (e.g., databases with dates of coverage, contact with study authors to identify additional studies) in the search and date last searched.	9-10
Search	8	Present full electronic search strategy for at least one database, including any limits used, such that it could be repeated.	10
Study selection	9	State the process for selecting studies (i.e., screening, eligibility, included in systematic review, and, if applicable, included in the meta-analysis).	8-11
Data collection process	10	Describe method of data extraction from reports (e.g., piloted forms, independently, in duplicate) and any processes for obtaining and confirming data from investigators.	10
Data items	11	List and define all variables for which data were sought (e.g., PICOS, funding sources) and any assumptions and simplifications made.	8-10
Risk of bias in individual studies	12	Describe methods used for assessing risk of bias of individual studies (including specification of whether this was done at the study or outcome level), and how this information is to be used in any data synthesis.	10
Summary measures	13	State the principal summary measures (e.g., risk ratio, difference in means).	10

For peer review only

Supplemental Material 3. PRISMA 2009 Checklist

Synthesis of results	14	Describe the methods of handling data and combining results of studies, if done, including measures of consistency (e.g., I^2) for each meta-analysis.	10

Section/topic	#	Checklist item	Reported on page #
Risk of bias across studies	15	Specify any assessment of risk of bias that may affect the cumulative evidence (e.g., publication bias, selective reporting within studies).	11
Additional analyses	16	Describe methods of additional analyses (e.g., sensitivity or subgroup analyses, meta-regression), if done, indicating which were pre-specified.	NA
**RESULTS**			
Study selection	17	Give numbers of studies screened, assessed for eligibility, and included in the review, with reasons for exclusions at each stage, ideally with a flow diagram.	11
Study characteristics	18	For each study, present characteristics for which data were extracted (e.g., study size, PICOS, follow-up period) and provide the citations.	12-22
Risk of bias within studies	19	Present data on risk of bias of each study and, if available, any outcome level assessment (see item 12).	12-22
Results of individual studies	20	For all outcomes considered (benefits or harms), present, for each study: (a) simple summary data for each intervention group (b) effect estimates and confidence intervals, ideally with a forest plot.	12-22
Synthesis of results	21	Present results of each meta-analysis done, including confidence intervals and measures of consistency.	NA
Risk of bias across studies	22	Present results of any assessment of risk of bias across studies (see Item 15).	18
Additional analysis	23	Give results of additional analyses, if done (e.g., sensitivity or subgroup analyses, meta-regression [see Item 16]).	NA
**DISCUSSION**			
Summary of evidence	24	Summarize the main findings including the strength of evidence for each main outcome; consider their relevance to key groups (e.g., healthcare providers, users, and policy makers).	22-27
Limitations	25	Discuss limitations at study and outcome level (e.g., risk of bias), and at review-level (e.g., incomplete retrieval of identified research, reporting bias).	28
Conclusions	26	Provide a general interpretation of the results in the context of other evidence, and implications for future research.	29
**FUNDING**			
Funding	27	Describe sources of funding for the systematic review and other support (e.g., supply of data); role of funders for the systematic review.	29

For peer review only

Supplemental Material 3. PRISMA 2009 Checklist

From: Moher D, Liberati A, Tetzlaff J, Altman DG, The PRISMA Group (2009). Preferred Reporting Items for Systematic Reviews and Meta-Analyses: The PRISMA Statement. PLoS Med 6(7): e1000097. doi:10.1371/journal.pmed1000097

For more information, visit: **www.prisma-statement.org**.

For peer review only

For peer review only - http://bmjopen.bmj.com/site/about/guidelines.xhtml

Supplemental Material 4. Table of Included Studies with Additional Information

Author	Country	Service, Intervention, Study description
Davidson et al (2019)	USA	Tablet trauma-focussed cognitive behavioural therapy (TF-CBT) included 11 activities that focussed on breathing retraining, muscle relaxation, affective regulation and cognitive coping; other activities are specific to the TF-CBT model, including psychoeducation relating to traumatic events, safety education, and gradual exposure.
Jager et al (2017)	The Netherlands	Patient-centred communication through Take Care study
Ougrin et a (2018)	UK	Supported Discharge Service Versus Inpatient Care Evaluation (SITE) project comprised of intensive community-based treatment, which involved customised care plans of intensive case management, community/home treatment, day care in hospital
Dion et al (2010)	Canada	Crisis Intervention Program that investigated the capability of emergency department staff to provide emergency assessments to adolescents with mental health issues presenting to the emergency department
Spenser et al (2009)	Canada	CHAT project which aimed to increase collaboration between community paediatricians and mental health clinicians: hired and integrated paediatrician within mental health team; surveyed clinicians about perceived needs; and provided continuing education based on identified needs
Ayton et al (2013)	UK	Audit Care Programme Approach (CPA) care plans within Child and adolescent mental health services (CAMHS) and identify service user involvement in all aspects of mental healthcare<br><br>This includes involving them in a needs assessment, developing and reviewing the care plan; and sharing responsibilities between the patient, carers, and relevant professional agencies.
Aupont et al (2013)	USA	Targeted Child Psychiatric Services that implemented a care model that facilitates adolescent's access to specialised mental health services, investigating whether the adolescent remains in specialised mental health services or returns to paediatric primary care
Simmons et al (2016)	Australia	*headspace* implemented an online decision aid for adolescent and youth depression was developed. The aim of the intervention was help young people make evidence-informed decisions about their treatment, make them feel involved, adhere to treatment and reduce symptoms, and increase satisfaction
Irvine (2020)	Ireland	Cross-sectional survey of child and adolescents' experiences with mental health care, using quality indicator clusters focussing on (i) information and access; (ii) facilities and services; and (iii) quality of care.

For peer review only

Jager et al (2014)	The Netherlands	In the Take Care study, Client-centred communication (measured using the Consumer Quality Index) affects adolescents participation and learning in their mental healthcare
Stevens et al (2009)	USA	Less intensive Telephone support services (TSS) intervention focussed on services and not symptoms
Anderson et al (2012)	Australia	Body signs, Relaxation, Active helpful thoughts, Victory you're your fears, Enjoy! Reward yourself (BRAVE) for Teenagers-Online project:<br><br>Develop working alliance during online cognitive behaviour therapy (CBT) treatment for youth anxiety.
Kapp et al (2017)	Switzerland	Identifying what influenced perceived quality of care (therapeutic alliance) in CAMHS
Cairns et al (2019)	Australia	In *headspace* services, influence of goal setting frequency and quality on patient retention
Ringle et al (2019)	USA	The Resiliency and Disease Management initiative, or an evidence-based practice that includes Child and Adolescent Texas Recommended Assessment Guidelines, a comprehensive list of service packages that indicates level of service intensity and evidence-based practice (e.g., CBT)
Sattler et al (2019)	USA	Non-profit provider of medical and mental health services<br><br>evidence-based assessments
Sattler et al (2016)	USA	Non-profit provider of medical and mental health services<br><br>use of evidence-based assessment techniques, including structured interviews, rating scales, DSM-IV-TR and agreement at primary care facilities, general mental health clinics, and specialty clinics for anxiety disorders
Higa-McMilan et al 2017)	USA	Use of Practices derived from the evidence base (PDEB)
Rukundo et al (2020)	Uganda	Two-year diploma programme that focusses on basic training of health care providers (psychiatrists, medical officers, psychologists, psychiatric clinical officers, occupational therapists, psychiatric nurses, general nurses, public health officers, social workers, human resource officers) towards assessing, recognising and managing child and adolescent mental health.
Bardach et al (2020)	USA	Using the Children's Core Set of quality measures for children and adolescents that have used mental health services

Acronyms: Trauma focussed cognitive behavioural therapy (TF-CBT); randomized controlled trial (RCT); Supported Discharge Service Versus Inpatient Care Evaluation (SITE); emergency department (ED); Child and adolescent mental health services (CAMHS); Care programme approach (CPA); Telephone support service (TSS); Body signs, Relaxation, Active helpful thoughts, Victory over your fears, Enjoy! Reward yourself (BRAVE); The Choice and Partnership Approach (CAPA); Practices derived from the evidence base (PDEB); Managing and Adapting Practice (MAP); Practices derived from the evidence base (PDEB)

For peer review only
